# Genetic architecture of main effect QTL for heading date in European winter wheat

**DOI:** 10.3389/fpls.2014.00217

**Published:** 2014-05-20

**Authors:** Christine Zanke, Jie Ling, Jörg Plieske, Sonja Kollers, Erhard Ebmeyer, Viktor Korzun, Odile Argillier, Gunther Stiewe, Maike Hinze, Sebastian Beier, Martin W. Ganal, Marion S. Röder

**Affiliations:** ^1^Department of Cytogenetics and Genome Analyses, Leibniz Institute of Plant Genetics and Crop Plant Research (IPK)Gatersleben, Germany; ^2^TraitGenetics GmbHGatersleben, Germany; ^3^KWS LOCHOW GmbHBergen, Germany; ^4^Syngenta Seeds S.A.S.Orgerus, France; ^5^Syngenta Seeds GmbHBad Salzuflen, Germany

**Keywords:** genome wide associations, *Triticum aestivum* L., photoperiodism, environmental adaptation, flowering time

## Abstract

A genome-wide association study (GWAS) for heading date (HD) was performed with a panel of 358 European winter wheat (*Triticum aestivum* L.) varieties and 14 spring wheat varieties through the phenotypic evaluation of HD in field tests in eight environments. Genotyping data consisted of 770 mapped microsatellite loci and 7934 mapped SNP markers derived from the 90K iSelect wheat chip. Best linear unbiased estimations (BLUEs) were calculated across all trials and ranged from 142.5 to 159.6 days after the 1st of January with an average value of 151.4 days. Considering only associations with a −log_10_ (*P*-value) ≥ 3.0, a total of 340 SSR and 2983 SNP marker-trait associations (MTAs) were detected. After Bonferroni correction for multiple testing, a total of 72 SSR and 438 SNP marker-trait associations remained significant. Highly significant MTAs were detected for the photoperiodism gene *Ppd-D1*, which was genotyped in all varieties. Consistent associations were found on all chromosomes with the highest number of MTAs on chromosome 5B. Linear regression showed a clear dependence of the HD score BLUEs on the number of favorable alleles (decreasing HD) and unfavorable alleles (increasing HD) per variety meaning that genotypes with a higher number of favorable or a low number of unfavorable alleles showed lower HD and therefore flowered earlier. For the vernalization gene *Vrn-A2* co-locating MTAs on chromosome 5A, as well as for the photoperiodism genes *Ppd-A1* and *Ppd-B1* on chromosomes 2A and 2B were detected. After the construction of an integrated map of the SSR and SNP markers and by exploiting the synteny to sequenced species, such as rice and *Brachypodium distachyon*, we were able to demonstrate that a marker locus on wheat chromosome 5BL with homology to the rice photoperiodism gene *Hd6* played a significant role in the determination of the heading date in wheat.

## Introduction

Heading date (HD) is one of the critical traits for the adaptation of bread wheat (*Triticum aestivum* L.) to diverse climatic environments and the cultivation in various regions and cropping seasons. The adaptability of wheat to a wide range of environments has been favored by allelic diversity in genes regulating growth habit and photoperiod response. Differences in the vernalization genes (*Vrn*) determine spring and winter wheat habits. The photoperiod genes (*Ppd*) play a major role in determining the flowering time and the sensitivity to photoperiodism. Earliness *per se* (*Eps)* genes influence flowering time independently from photoperiodism.

On a molecular level, regulation networks for heading and flowering are conserved between model species, such as *Arabidopsis* (Andrés and Coupland, 2012), as well as in dicotyledonous and monocotyledonous crop plants (Jung and Müller, 2009) including the temperate cereals (Cockram et al., 2007; Trevaskis et al., 2007; Distelfeld et al., 2009).

Positional cloning identified *Ppd-H1*, the major determinant of barley photoperiod response, as a pseudo-response regulator, which is an ortholog of the *Arabidopsis* photoperiod pathway gene *CONSTANS* (Turner et al., 2005). In wheat, an orthologous gene was identified as the *Ppd-D1* gene on chromosome 2D (Beales et al., 2007). A semi-dominant mutation, *Ppd-D1a* widely used in the “green revolution,” converts wheat from a long day (LD) to a photoperiod insensitive (day neutral) plant, providing adaptation to a broad range of environments. Recently it was shown that the bolting locus B of sugar beet, distinguishing annuals from biennials, is also a pseudo-response regulator gene named *BOLTING TIME CONTROL 1* (*BvBTC1*) with similarity to the *CONSTANS* gene of Arabidopsis and *Ppd-H1* in barley (Pin et al., 2012). Another photoperiodism gene, *Ppd-B2*, which was detected when plants were exposed to a long photoperiod, was mapped on chromosome 7BS in wheat (Khlestkina et al., 2009).

Similarily, the molecular mechanisms for the requirement of vernalization have been identified (Trevaskis et al., 2007; Distelfeld et al., 2009) in wheat. Natural variation in vernalization requirement in the temperate cereals is mainly associated with allelic differences in the *VRN1*, *VRN2*, and *VRN3* vernalization genes. *VRN1* encodes a MADS-box transcription factor with high similarity to *Arabidopsis* meristem identity genes *APETALA1*, *CAULIFLOWER* and *FRUITFUL* (Yan et al., 2003; Distelfeld et al., 2009). *VRN2*, a dominant repressor of flowering, is down-regulated by vernalization. The *VRN2* region includes two similar ZCCT genes encoding proteins with a putative zinc finger and a CCT domain that have no clear homologs in *Arabidopsis* (Yan et al., 2004; Distelfeld et al., 2009). The vernalization gene *VRN3* encodes a RAF kinase inhibitor like protein with high homology to *Arabidopsis* protein *FLOWERING LOCUS T* (*FT*) (Yan et al., 2006; Distelfeld et al., 2009).

The presence of earliness *per se* genes (*Eps*) has been demonstrated by QTL-mapping studies in barley and wheat since a long time (Laurie et al., 1995; Worland, 1996). Only recently the molecular identification of two *EARLY MATURITY* genes, *eam8* and *eam10*, has been reported in barley (Faure et al., 2012; Zakhrabekova et al., 2012; Campoli et al., 2013). Earliness *per se* genes have been fine-mapped in diploid or hexaploid wheat on chromosomes 1A and 3A (Faricelli et al., 2010; Gawronski and Schnurbusch, 2012).

Several QTL and meta-QTL mapping studies showed that in wheat, besides the known major loci, a wealth of additional chromosomal regions affect the flowering time (Sourdille et al., 2000; Hanocq et al., 2004, 2007; Griffiths et al., 2009; Rousset et al., 2011). Co-location of QTLs for agronomic traits, such as post-anthesis leaf senescence, grain yield or grain protein concentration with QTL for flowering time indicated pleiotropic effects of anthesis date (Bogard et al., 2011). Also in barley a number of flowering time QTL were associated with agronomic traits (Wang et al., 2010).

While with bi-parental mapping studies only a limited number of parental lines can be investigated, genome wide association studies (GWAS) are suitable for the monitoring of a larger germplasm panel (Zhu et al., 2008). The method is based on the meiotic events which occurred during the entire development of the lines and which results in an increased genetic resolution determined by the extent of linkage disequilibrium (LD) of the species under investigation (Hamblin et al., 2011). Whole-genome association mapping was applied in wheat for ear emergence (Le Gouis et al., 2012), as well as for yield and agronomic traits (Neumann et al., 2011; Reif et al., 2011; Wang et al., 2012) and resistance to pathogens (Crossa et al., 2007; Maccaferri et al., 2010; Miedaner et al., 2011; Yu et al., 2011, 2013; Letta et al., 2013; Kollers et al., 2013a,b).

The goal of our study was to map marker-trait associations (MTAs) for HD in a panel of European winter wheat varieties and to identify markers suitable for marker assisted selection. We were interested to compare the MTAs detected with genome wide SSR (simple sequence repeat) markers to the pattern of MTAs detected by a SNP (single nucleotide polymorphism) array. Finally, we exploited the synteny of the SNP marker sequences to other grass species with complete genome sequence, such as rice and *Brachypodium distachyon*, in order to detect relationships to already described genes connected to the regulation of photoperiodism and flowering time.

## Materials and methods

### Plant material and phenotyping

The plant material, consisting of 358 European winter wheat varieties plus 14 spring wheat varieties as an outgroup, is described in more detail in Kollers et al. (2013a). Field trials were conducted in the season 2008/2009 in Andelu/France (09.AND), Seligenstadt/Germany (09.SEL) and Wohlde/Germany (09.WOH) and in the season 2009/2010 in Andelu/France (10.AND), Janville/France (10.JAN), Saultain/France (10.SAU), Seligenstadt/Germany (10.SEL) and Wohlde/Germany (10.WOH) by applying an alpha design with two replications per site. Both winter and spring varieties were sown in autumn and HD was recorded as the developmental stage at that time, by counting days from the 1st of January, when ears of approximately half of the genotypes were fully visible (Supplemental Table 1).

### Molecular data analysis, genetic mapping and analysis of synteny

For marker-trait analysis a set of 732 microsatellite markers, resulting in 770 different loci spread across all chromosomes of wheat was used. Of these 770 loci, 635 loci were mapped and 135 loci were unmapped. Since the microsatellites are multi-allelic, they amounted to 3176 alleles. More details about this data set and the description of LD and population structure are provided in Kollers et al. (2013a). For SNP-analysis a novel 90k Infinium chip (90k iSELECT) was genotyped on all 372 varieties (Cavanagh et al., 2013; Wang et al., 2014). This resulted in a total of 21742 scorable and polymorphic markers on our association panel by considering all polymorphic markers with a minor allele frequency (MAF) >0.03. Of these markers, only the 7934 mapped markers were included in the association analysis, while the unmapped markers were not used for association analysis. The SSR-markers were mapped on the ITMI-population (International Triticeae Initiative) based on recombinant inbred lines between the parents W7984 (synthetic wheat) × Opata M85 (Röder et al., 1998; Ganal and Röder, 2007), while the SNP markers were mapped on 138 lines of a newly created doubled-haploid population of the same parents (Sorrells et al., 2011; Poland et al., 2012). Map construction was performed using the software package Joinmap 4.1. Both maps have different recombination values, and currently only few common markers are available, which makes comparisons difficult. For display a reduced version of the SNP-map was used containing all relevant markers with MTAs for HD.

In order to establish the synteny of interesting MTA loci to rice, a BLAST X (cutoff: *e*-value of 10E-2) was conducted against the data base of MSU Rice Genome Annotation Project Release 7.0 (http://rice.plantbiology.msu.edu/) for all SNP markers with significant (–log_10_ (*P*-value) ≥ 3.0) MTAs for HD. For the blast search the flanking sequences of the SNP markers (101–201 bp in length) according to Wang et al. (2014) were used. The resulting 3877 syntenic relationships were filtered for chromosomal synteny as described by Salse et al. (2009) resulting in 956 syntenic relationships. For comparison to literature data in some cases the ID converter (http://rapdb.dna.affrc.go.jp/tools/converter/run?type=rap-msu;id=Os11g0157100) was used in order to compare to locus designations of the RAP-DB rice annotation project database (http://rapdb.dna.affrc.go.jp/).

For detecting the synteny to *Brachypodium distachyon* a BLAST X (cutoff: *e*-value of 10E-2) was conducted against version 1.2 of the MIPS annotation (http://mips.helmholtz-muenchen.de/plant/brachypodium/download/index.jsp) resulting in 3404 syntenic relationships. Those were filtered according to the expected chromosomal synteny (The International Brachypodium Initiative, 2010) resulting in 1575 syntenic relationships.

As candidate genes the photoperiodism gene *Ppd-D1* (Beales et al., 2007) and the vernalization genes *Vrn-B1* and *Vrn-D1* (Zhang et al., 2008) were genotyped on all varieties.

### Statistical analysis and association mapping

Each year-location combination was considered as an environment in our study. For each environment and genotype the adjusted mean of two replications was calculated as the phenotypic data using GenStat 13th edition as

y=μ+replication+genotype+block+e

with replication and genotype as fixed factors and block as random factor and block nested within replication; μ represents an overall mean and e is a residual term.

In addition, best linear unbiased estimations (BLUEs) across all eight environments were calculated using the software package GenStat 14th edition (VSN International, Hemel Hempstead, Hertfordshire, UK) as described in Kollers et al. (2013a) with

y=μ+genotype+environment+e

with genotype and environment as fixed effects; μ represents an overall mean and e is a residual term. Since the datasets for all environments were complete and balanced, the BLUEs, in fact, equaled the arithmetic means across environments.

For calculating genotype-phenotype associations a minor allele frequency (MAF) threshold of 3% (equaling 11 varieties) was applied to all markers. A mixed model for association mapping was calculated using the software package GenStat 14th edition as described in Kollers et al. (2013a) by applying a kinship matrix as relationship model.

$$\begin{array}{l} {\text{P}}_{\text{i}}=\mu +{\text{x}}_{\text{i}}\alpha +{\text{G}}_{\text{i}}+\text{e}\\ \text{with }{\text{G}}_{\text{i}}\sim \text{N}(0,2\text{K}\sigma_{\text{g}}^{2}),\text{error}\sim \text{N}(0,\sigma^{2}) \end{array}$$

x_i_ is the marker score for cultivar i, α is the marker fixed effect, μ represents an overall mean, e is a residual term and G_i_ represents the score of genotype corrected by kinship matrix (K) to structure random genotypic effects.

The Loiselle kinship matrix was calculated for 155 SSR markers, equally distributed on the genome, by using the software package SPAGeDi (Hardy and Vekemans, 2002). This kinship matrix was applied to correct for false positives for calculating MTAs with SSR as well as with SNP markers as described by Matthies et al. (2012). The threshold of Bonferroni correction for multiple testing was calculated by dividing *P* < 0.01 with the number of SSR or SNP markers used for the analysis.

Additive effects and marker effects (*r*^2^) were estimated using the software package TASSEL 3.0.

Spearman rank order correlations and ANOVA using the adjusted means of the eight environments were calculated with the software package SigmaPlot 11.0. The heritability was calculated from the variance components according to the formula: *H*^2^ = Var (genotype)/(Var (genotype) + Var (error)/no. of locations) with variance components calculated with the software package SPSS v. 19. This software was also used to conduct a trait *Post-hoc* test according to Tukey B.

## Results

### Description of phenotypic data

**Figure 1:**
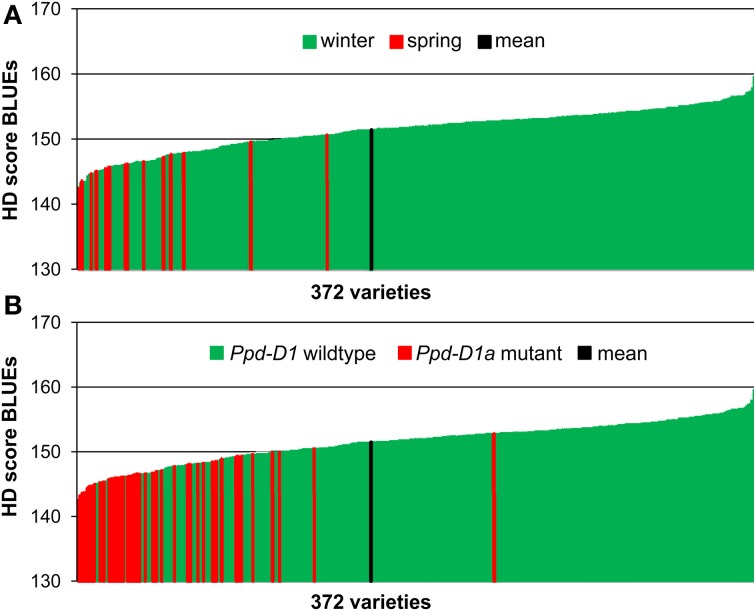
**Phenotypic distribution of HD score BLUEs in 372 wheat varieties**. The BLUEs of the HD score were calculated across eight environments. A low score indicates earlier heading date. HD sore BLUEs were arranged according to the growth habit **(A)** or to the distribution of the *Ppd-D1* wildtype or *Ppd-D1a* mutant gene **(B)**.

The phenotypic data for 358 European winter wheat varieties plus 14 spring wheat varieties were based on field evaluations in eight environments. The resulting best linear unbiased estimations for heading time across all environments ranged from 142.5 to 159.6 days after 1st of January with an average of 151.4 days (Supplemental file 1). All 14 spring varieties, which had been sown at the same time as the winter varieties were found in the early segment of HD (Figure 1A). Also all 53 varieties carrying the photoperiodism insensitive mutant of gene *Ppd-D1* on chromosome 2DS (Beales et al., 2007) were in the first half of the phenotypic distribution, with the exception of winter wheat variety “Paledor,” which was found in the second half of the phenotypic distribution (Figure 1B). The Spearman Rank Order correlation coefficients of the HD scores among the environments and with the BLUEs ranged from 0.843 to 0.973 (*P* < 0.001), indicating a high reproducibility of the ranking of varieties grown in different locations (Supplemental file 2). The analysis of variance (ANOVA) was significant for genotype as well as environment (Supplemental file 3). A Tukey *B*-test detected six different class means for the environments ranging from 144.5 to 161.5 days, which is also reflected in a broad sense heritability of *H*^2^ = 0.609 (Supplemental file 4).

### Detection of marker-trait associations (MTAs)

MTAs were calculated separately for each environment plus the resulting BLUEs by employing a mixed linear model with a kinship matrix. Two sets of genotypic data were used: First, a set of 732 microsatellite markers (SSRs) resulting in 770 loci spread across the 21 chromosomes of wheat, and secondly, a set of 7934 SNP markers coming from the 90K wheat iSELECT array. While the SNP markers represent a bi-allelic marker system, the microsatellites provide multiple alleles per locus resulting in a total number of 3176 alleles. The microsatellite data were described in former whole genome association studies (Kollers et al., 2013a,b) and provide good genomic coverage of all chromosomes. The SNP data were mapped to all chromosomes, but due to the lack of polymorphism, the chromosomes of the D-genome were less covered than those of the genomes A and B (Supplemental file 5).

**Figure 2:**
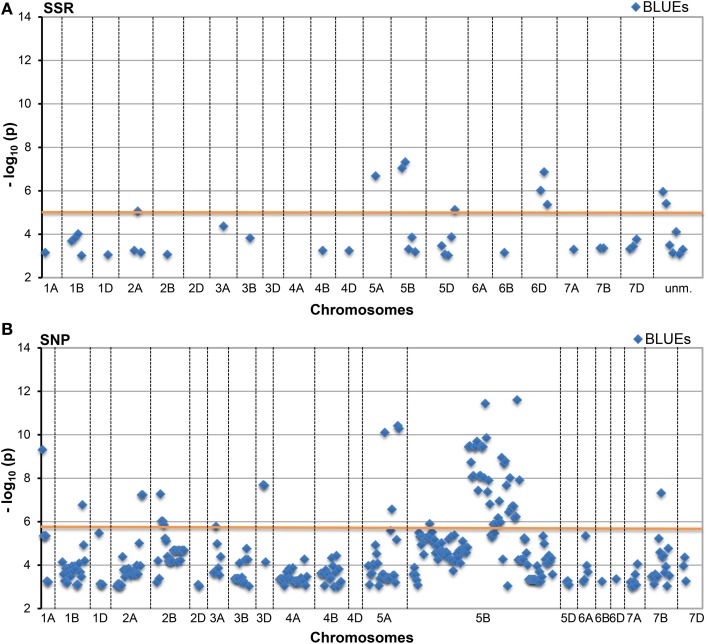
**Manhattan Plots of (A) SSR and (B) SNP marker alleles associated with HD BLUEs**. This plot presents significant alleles associations at threshold −log_10_ (*P*-value) ≥ 3.0 for BLUEs sorted according to their chromosomal location. The red line indicates the threshold −log_10_ (*P*-value) ≥ 4.82 (SSR) and ≥ 5.89 (SNP), respectively, for Bonferroni correction.

A total of 340 SSR and 2983 SNP MTAs reached a standard threshold of log_10_(*P*-value) ≥ 3.0 (corresponding to a *P*-value < 0.001). These included 42 BLUEs for the SSRs and 326 BLUEs for the SNPs. After applying a Bonferroni correction for multiple testing (with α = 0.01), a -log_10_(*P*-value) ≥ 4.82 for SSR and a −log_10_(*P*-value) ≥ 5.89 for SNP were considered as significant. After this correction, a total of 72 SSR and 438 SNP MTAs remained significant (Table 1, Figure 2, Supplemental files 5–7), which included 10 BLUEs for the SSRs and 51 BLUEs for the SNPs. A total of 79 different marker loci were involved in MTAs detection for the SSR markers and 758 marker loci for SNP markers corresponding to a −log_10_(*P*-value) ≥ 3.0 (Supplemental file 5). Since many marker loci co-segregated or were closely linked in the genetic map, marker loci with distances ≤ 1.0 cM were combined. When considering only the BLUEs with log_10_(*P*-value) ≥ 3.0, the number of combined marker loci was 30 for the SSRs and 92 for the SNPs (Supplemental file 5).

**Table 1:** **Number of MTAs per environment for the SSR marker and the SNPs on the 90K iSelect chip**.

**Environments**	**SSR**		**90K iSelect**	
	**–log_10_ (*P*-value) ≥ 3.0**	**–log_10_ (*P*-value) ≥ 4.82**	**–log_10_ (*P*-value) ≥ 3.0**	**–log_10_ (*P*-value) ≥ 5.89**
Andelu (2009)	45	9	325	60
Seligenstadt (2009)	29	5	254	43
Wohlde (2009)	30	9	337	39
Andelu (2010)	34	4	253	27
Janvielle (2010)	23	3	232	33
Saultain (2010)	44	8	290	47
Seligenstadt (2010)	52	13	583	94
Wohlde (2010)	41	11	383	44
BLUEs	42	10	326	51
Sum	340	72	2983	438

**Figure 3:**
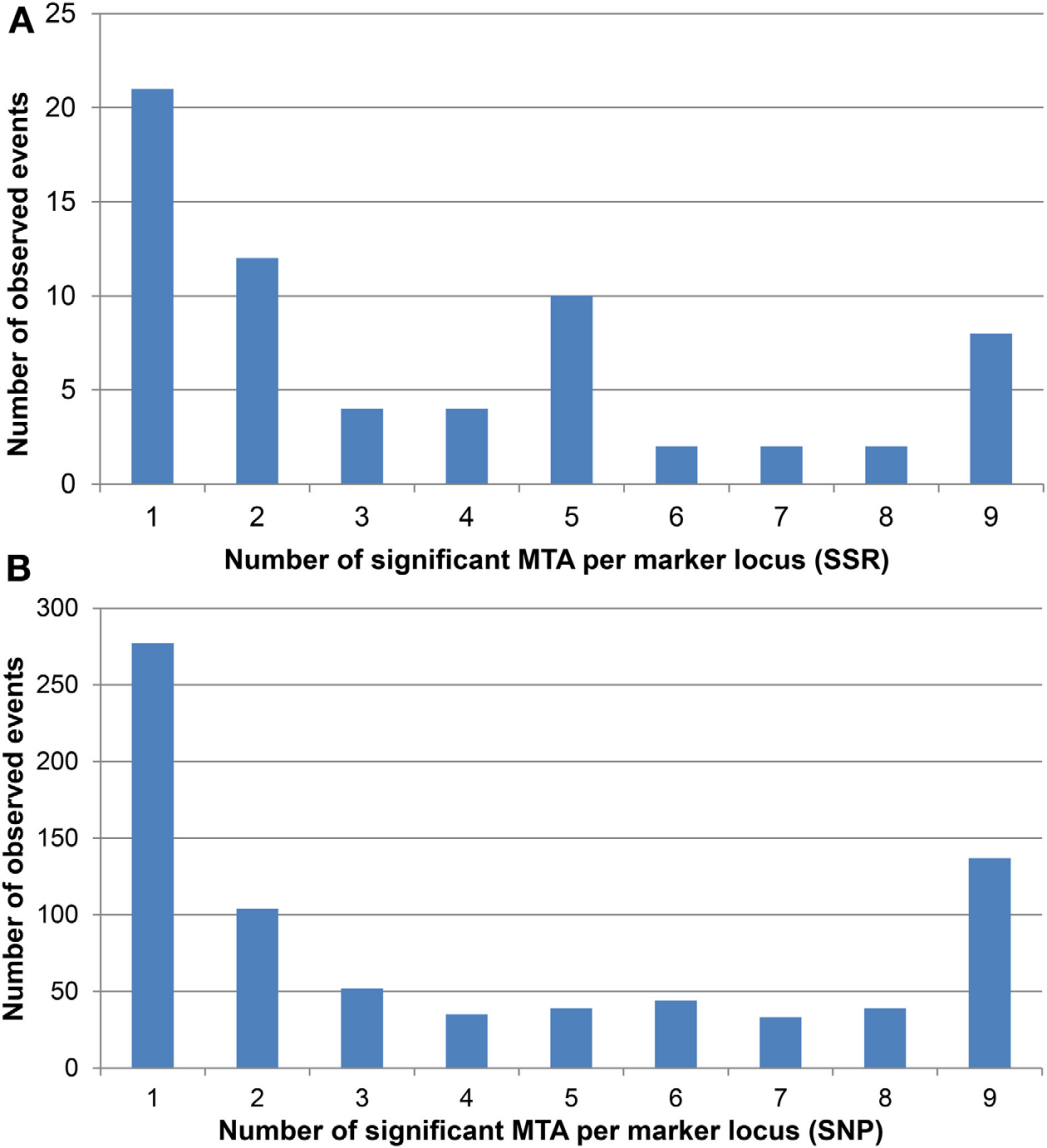
**Number of significant MTA for each (A) SSR and (B) SNP marker locus**. The significant MTA per marker locus range from one to a maximum of nine according to the eight environments plus BLUEs.

Many marker loci were significant for several environments (Figure 3, Supplemental file 8) with up to nine significant MTAs per marker locus (eight environments plus BLUEs). The number of significant MTAs varied considerably among the various chromosomes. For both marker types the highest number of significant MTAs was detected on chromosome 5B before Bonferroni correction, while after Bonferroni correction most SSR loci were significant on chromosome 6D (Supplemental file 5).

The comparison of the SSR map with the SNP map is still difficult, because the two maps were constructed on different mapping populations and contain only few common markers. Based on the mapping positions, some MTAs can be matched between SSR and SNP markers. An example is the MTA with SSR marker GWM1130 at the distal end of chromosome 1BS and the SNP marker Kukri_c38553_173. Overall, many additional marker loci were significant for the SNPs as compared to the SSR markers.

**Figure 4:**
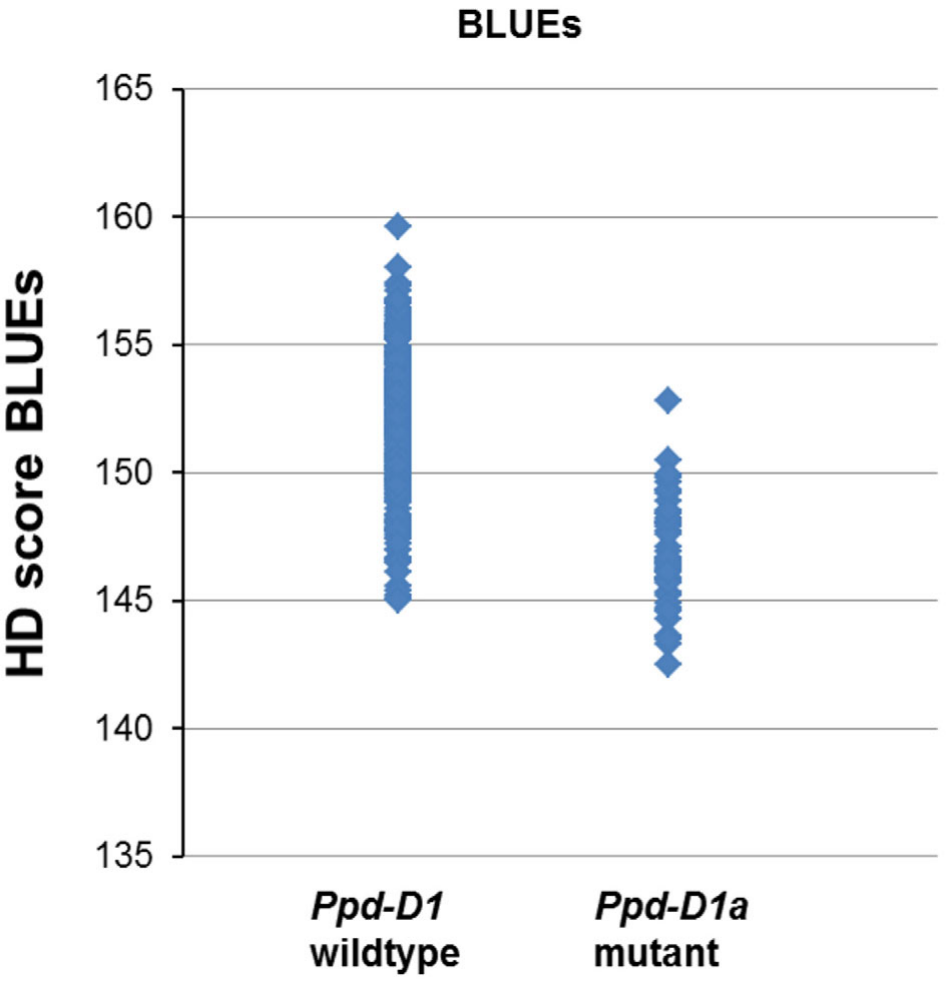
**Allelic effects for *Ppd-D1* in a population of 372 European wheat varieties**. Varieties carrying the mutant allele *Ppd-D1a* showed a decreased HD score BLUES resulting in an earlier heading.

Highly significant MTAs were detected for the photoperiod sensitivity gene *Ppd-D1* on chromosome arm 2DS. In all environments and the BLUEs the photoperiod insensitive mutant allele *Ppd-D1a* led to a decreased HD score, which means earlier heading (Figure 4, Supplemental file 9). The mutant allele of *Ppd-D1a* was detected in a total of 53 varieties including five spring varieties (Supplemental file 1). Additionally, the candidate gene markers for *Vrn-B1* and *Vrn-D1* were genotyped. While *Vrn-D1* was monomorphic for all winter varieties and a second allele detected in only two spring varieties (Supplemental file 1), *Vrn-B1* had a dominant allele for three spring varieties, but also three winter varieties (Buteo, Discus and Lona). Since both markers were below the threshold of minor allele frequency, they were not included in the regular analysis for MTAs. When they were tested for associations without setting a MAF, no significant association results were detected for *Vrn-B1* and significant MTAs in two environments were detected for *Vrn-D1* (Supplemental file 10).

### Additive effects for favorable and unfavorable alleles

In the following section, marker alleles with a negative additive effect leading to earlier heading will be referred to as “favorable alleles” and vice versa marker alleles leading to later heading as “unfavorable alleles.” We are aware, that earlier heading is not favorable in all circumstances; the designation is mainly meant to facilitate the following description of the allele effects.

**Figure 5:**
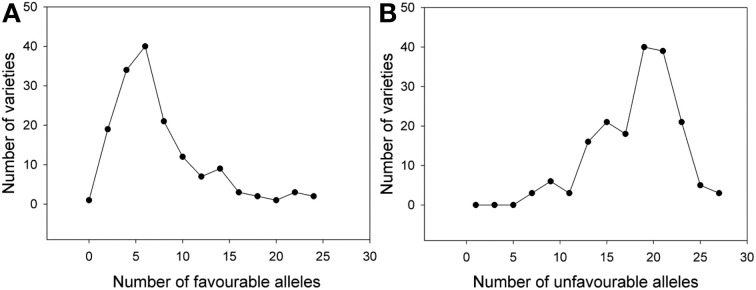
**Frequency of (A) favorable HD alleles and (B) unfavorable HD alleles from SSR markers in individual varieties**. Most of the varieties carried between zero to ten favorable alleles decreasing the heading date and between ten to 25 unfavorable alleles increasing the heading date.

**Figure 6:**
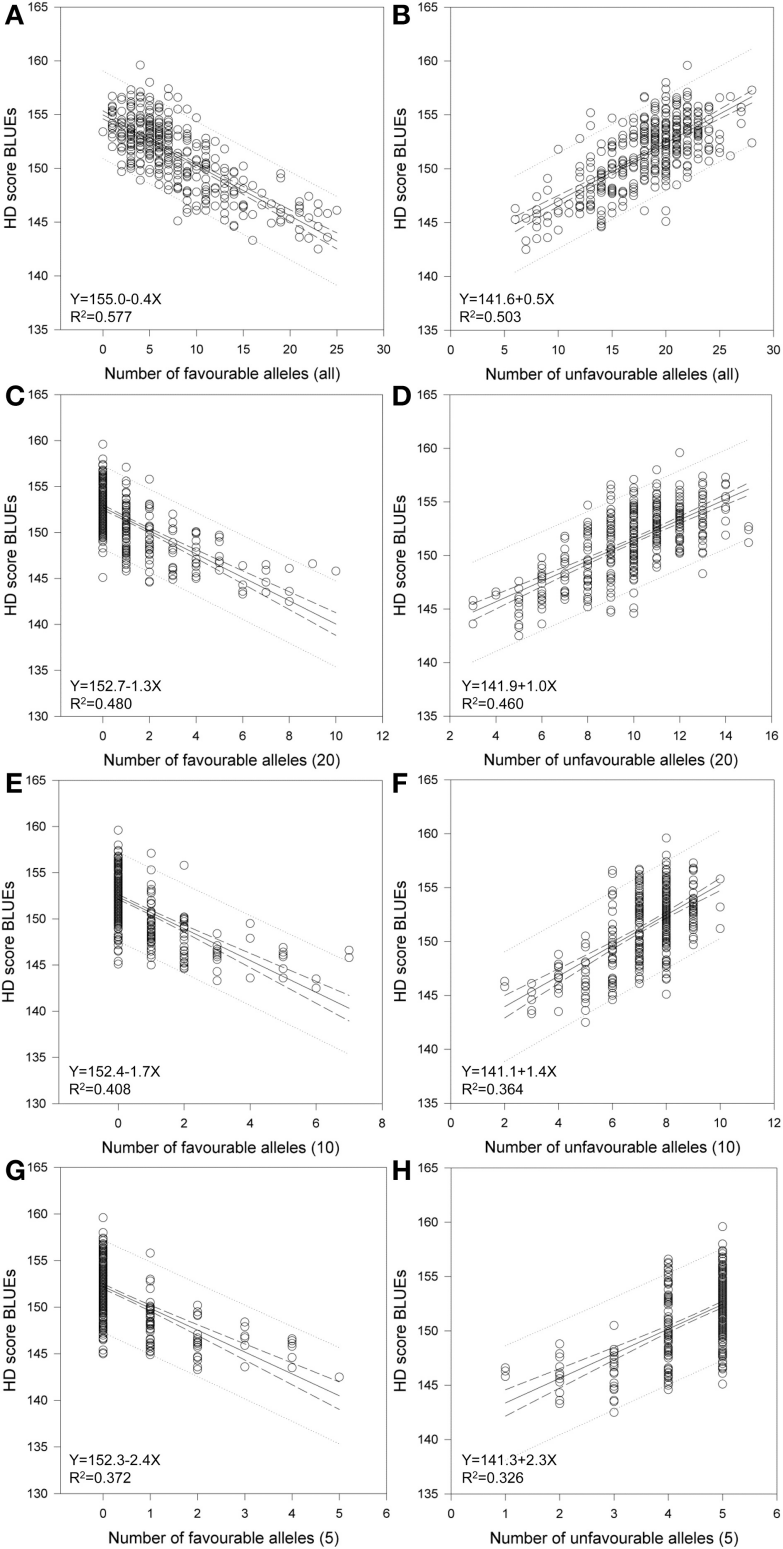
**Regression of favorable and unfavorable HD alleles**. Linear regression resulted in a relationship of HD-BLUEs score and number of favorable and number of unfavorable alleles in 372 wheat varieties. The calculations were performed for **(A)** all favorable and **(B)** all unfavorable alleles and included the SSR marker with significant association with a −log_10_ (*P*-value) ≥ 3.0. Additional calculation were done by taking the **(C)** 20 best and **(D)** 20 worst alleles, the **(E)** 10 best and **(F)** 10 worst alleles as well as the **(G)** 5 best and **(H)** 5 worst alleles, which included the candidate gene *Ppd-D1*.

Considering the SSR markers, the varieties contained between zero to 25 favorable alleles and between six to 28 unfavorable alleles (Figure 5). A significant Spearman Rank Order correlation of *R* = −0.697 (*P* = 0.00000020) existed between the HD BLUEs score and number of favorable alleles; for the HD BLUEs score and the number of unfavorable alleles the Spearman Rank correlation coefficient was *R* = 0.642 (*P* = 0.00000020). Linear regression showed a dependence of the HD BLUEs score from the number of favorable alleles with *R*^2^ = 0.577 and *Y* = 155.0 − 0.4X; for the unfavorable alleles *R*^2^ = 0.503 and *Y* = 141.6 + 0.5X was observed (Figure 6). This means that varieties with a higher number of favorable alleles and a lower number of unfavorable alleles have an earlier heading time. The regression of favorable minus unfavorable alleles against the HD BLUEs score was *Y* = 148.5 − 0.3X with *R*^2^ = 0.603 (Supplemental file 11).

We calculated the same regressions by taking the best or worst 20, 10 or 5 marker alleles into account (Figure 6, Table 2). These included the SSR markers with significant associations with a −log_10_(*P*-value) ≥ 3.0 and the candidate gene *Ppd-D1*. The selection was based on the mean additive effect as described in Supplemental file 5. Even with only five marker alleles, significant regressions with *R*^2^ = 0.372 for the favorable alleles and *R*^2^ = 0.326 for the unfavorable alleles were observed. Therefore the chosen marker alleles are good candidates for adapting the HD in breeding programs by marker assisted selection.

**Table 2:** **List of the best favorable and worst unfavorable alleles**.

**Marker alleles**	**Chromosome (linked genes)**	**Position**	**Alleles belong to the**					
			**20 best**	**10 best**	**5 best**	**20 worst**	**10 worst**	**5 worst**
GWM1130_109bp^*^	1B	0	x					
GWM1130_115bp^*^	1B	0				x	x	x
BARC0240_231bp	1B	36.1				x		
GWM3166_153bp	1B	175.7	x					
WMC0732c_295bp	1D	132.4	x					
WMC0522_200bp	2A (*Ppd-A1*)	88.3	x	x				
GWM4167_217bp	2B (*Ppd-B1*)	40				x		
BARC0160_111bp	2B	80.4				x	x	
CFD0056c_250bp	2D	20.2				x		
GWM0988_180bp	2D	84.5				x		
CFD0168_256bp	2D	160.3				x	x	x
Ppd_insensitive	2D	unm.	x	x	x			
Ppd_sensitive	2D	unm.				x	x	x
WMC0264_141bp^*^	3A^1^	131.3	x	x				
WMC0264_148bp^*^	3A^1^	131.3				x		
WMC0808_147bp	3B^1^	67.5	x					
GWM0160a_181bp	4A	186.4	x					
GWM4636_233bp	4B^1^	59.4	x					
WMC0285_293bp	4D	0	x	x				
GWM0291_176bp	5A (*Vrn-A2*)	231	x	x	x			
WMC0160b_137bp	5B^1^ (Hd6-related gene)	158.4				x	x	
WMC0783_179bp	5B	219.8				x		
WMC0215_208bp	5D	200.5	x					
GDM0063_147bp	5D	265.4				x		
WMC0161b_184bp	5D	301.3				x		
GWM4047_194bp	6B	0				x	x	
GWM0825b_122bp	6B	34.3				x	x	
GWM1391_158bp^*^	6D	0				x	x	x
GWM1391_160bp^*^	6D	0	x	x	x			
CFD0019c_313bp	6D	130.9	x	x	x			
BARC0204b_500bp	6D	194	x					
GWM0983b_130bp^*^	7B	54				x	x	x
GWM0983b_133bp^*^	7B	54	x	x				
BARC0182_118bp	7B	176				x	x	
GWM0428_145bp	7D	228.2				x		
WMC0014_267bp	7D	274.2				x		
BARC0261_170bp	Unmapped	–	x	x	x			
CFA2263_123bp	Unmapped	–	x					
WMC0327_209bp	Unmapped	–	x					
WMC0349_118	Unmapped	–	x	x				

* Markers with positive and negative additive effects,

1 *coincides with meta-QTL described by Griffiths et al. (2009)*.

### Exploitation of synteny to rice and *Brachypodium distachyon*

After conducting a BlastX to the rice genomic sequence and filtering for the synteny relationships between wheat and rice described by Salse et al. (2009) a total of 956 syntenic relationships between significant wheat SNP markers and the rice genome were established (Supplemental file 12). For *Brachypodium distachyon*, a total of 1575 syntenic relationships to the wheat markers were found after filtering for synteny according to the described chromosomal relationships (The International Brachypodium Initiative, 2010) (Supplemental file 13).

In the publication of Higgins et al. (2010) all known genes related to flowering time pathways were blasted to *Brachypodium* and rice. A comparison of our list of syntenic rice loci (Supplemental file 12) to their detected homologs gave two direct hits for the wheat marker Kukri_c10016_369 to two rice loci at LOC_Os03g10940.1 and LOC_Os03g55490.1. Both genes are coding for expressed putative protein casein kinase II subunit alpha-2, which both have homology to the rice gene *Hd6* located at LOC_Os03g55389 (Takahashi et al., 2001). Also for *Brachypodium* homologs four direct hits with the same wheat marker Kukri_c10016_369 were found (Bradi1g07750.1, Bradi1g07810.1, Bradi1g59010.1, Bradi1g70690.1), with all four genes belonging to the *Hd6* gene family. *Hd6* was cloned as a rice quantitative trait locus involved in photoperiod sensitivity and is thought to be involved in the plant phototransduction pathway. Wheat marker Kukri_c10016_369 was highly significant, even after Bonferroni correction, in all eight environments plus BLUEs and is part of a cluster of significant markers on chromosome 5B. In an analysis of LD it was shown, that LD existed between Kukri_c10016_369 and the highly significant SSR markers WMC160 and BARC232, especially the alleles discovering MTAs, WMC160_137 bp, WMC160_190 bp, BARC232_197 bp, and BARC232_232 bp, while no LD existed with *Vrn-B1* (Supplemental file 14). Therefore the MTAs discovered by those SSR alleles are not based on LD to *Vrn-B1* but most likely on LD to an *Hd6* related gene in wheat. It can be concluded, that the gene from which Kukri_c10016_369 was derived, is an *Hd6*-related gene in wheat, which itself has a significant impact on HD or is in LD with another gene affecting HD.

## Discussion

### Comparison of MTAs discovered with SSR and SNP markers

The chosen approach led to the discovery of a number of highly significant MTAs for HD in European winter wheat. In comparison to other traits, which were analyzed in the same set of varieties and molecular markers, the number of significant MTAs for HD was lower and less loci were involved. For resistance to *Fusarium* head blight a total of 794 significant MTAs [−log_10_(*P*-value) ≥ 3.0], which included 323 SSR alleles, were detected in four environments (Kollers et al., 2013a), while for resistance to *Septoria tritici* blotch 115 MTAs were significant [−log_10_(*P*-value) ≥ 3.0] involving 68 microsatellite loci in two environments (Kollers et al., 2013b). For HD, 340 MTAs detected by 79 SSR loci were significant [−log_10_ (*P*-value) ≥ 3.0] in eight environments (Table 1). In a previous genome-wide association study involving a 227-wheat core collection and 760 molecular markers, consisting of mainly DArT markers, 62 markers individually associated to earliness components corresponding to 33 chromosomal regions, were identified (Le Gouis et al., 2012). This number corresponds well to the 30 loci identified in our study by SSRs, when considering the BLUEs only and when adjacent markers were combined to unified loci (Supplemental files 5, 6B, 7B). A meta-QTL analysis of the genetic control of ear emergence in elite European winter wheat germplasm discovered 19 meta-QTL regions (Griffiths et al., 2009).

Many marker loci were detected in two or more environments (Figure 3). This observation indicates the impact of major genes in shaping the genetically determined pattern of HD in winter wheat. It is also an indicator of a high reproducibility of the ranking of varieties considering the phenotypic data, which was confirmed by the high correlations observed between the environments (Supplemental file 2), though the environments covered a range of geographical latitudes (48.2 to 54.4°N; Supplemental file 1) and various micro-climates in France and Germany.

Overall, the used number of SNP markers was higher with 7934 SNP markers compared to 770 SSR loci with a total of 3176 SSR alleles. After Bonferroni correction, 90 SSR markers remained significant as compared to 438 for the SNPs. These included 10 BLUEs for SSR and 51 BLUEs for SNPs (Supplemental files 5, 6B, 7B).

Though the overall number of SNP markers was higher than the SSR markers, there was less coverage for specific chromosomes like 4D and 6D, and many co-segregating loci resulted in a reduced number of haplotypes. Like the SSRs, the SNP markers often detected significant MTAs for HD in various environments with 137 SNP markers detecting all eight environments plus BLUEs (Figure 3B). Often SNPs, which co-segregated in the genetic map, were all involved in MTA detection, resulting in clusters of significant markers (Supplemental file 8). The prerequisite for a detailed comparison of the significant SSR and SNP loci is a highly integrated map for both marker systems, which currently is not available yet. By comparing the chromosomal locations of the SNP and SSR maps (Supplemental file 8), it becomes obvious that several novel chromosomal locations were detected by the SNPs compared to the SSRs. Examples are a cluster of significant SNP markers at the distal end of chromosome 1AL (RAC875_c21411_162, wsnp_BE444305A_Td_2_1, wsnp_RFL_Contig3542_3718200, RAC875_c12348_720) and a cluster of highly significant markers on the distal end of chromosome 3DS (Excalibur_c19658_127, Kukri_c24488_431, Kukri_rep_c94244_223).

### Candidate genes for MTAs with SSR

The presence of detailed mapping information of the SSR markers in various maps (Somers et al., 2004; Ganal and Röder, 2007; http://wheat.pw.usda.gov/GG2/index.shtml) allowed the comparison of our association results to the mapping positions of known candidate genes. MTAs most likely corresponding to the series of photoperiodism genes *Ppd* on the short arms of the homeologous group 2 chromosomes were detected for chromosome 2A (markers WMC177 and WMC522) and chromosome 2B (marker GWM4167). *Ppd-B1* was previously mapped in the interval of GWM257 and GWM148 (Mohler et al., 2004), which includes marker GWM4167 in our map. The marker for candidate gene *Ppd-D1* was the most significant marker based on the observed additive effects, however no significant SSR markers in the expected region on chromosome 2DS in the vicinity of marker GWM261 (Pestsova and Röder, 2002) were observed. One possible reason may be the existence of a 21 centiMorgan gap in the genomic region between WMC112 and BARC168. If *Ppd-D1* is located in this gap, the extent of LD may not reach the flanking markers. An LD plot showed no LD with *r*^2^ > 0.1 between the alleles of markers GWM261,WMC112 or BARC168 and the *Ppd-D1* candidate gene (Supplemental file 15). The agronomic effects described for *Ppd-D1* depended very much on the trial sites. In the UK, the 2D chromosome carrying *Ppd-D1* reduced yield about 5–10%, while in Yugoslavia the same genotypes increased yield about 30% (Worland et al., 1998). The advantages of earlier heading of *Ppd-D1* insensitive varieties in Southern European countries were attributed to an escape of heat and drought during summer. The genotyping of the candidate marker for *Ppd-D1* indeed showed that the insensitive mutant allele is mainly present in varieties originating from South France (Supplemental file 1). *Ppd-B1* (old nomenclature *Ppd2*) was described as a weaker gene for photoperiod insensitivity than *Ppd-D1* with a strong influence of the environmental conditions on the agronomic effects (Worland et al., 1998). For central European varieties, where the effects of *Ppd-D1* are too strong, *Ppd-B1* may provide a moderate gene for the adaptation to hot and dry summers. An epistatic interaction between *Ppd-B1* and *Ppd-D1* was described in a doubled haploid mapping population (Hanocq et al., 2004). We found in our list of the markers with the strongest additive effects besides the *Ppd-D1* candidate gene also GWM4167 associated with *Ppd-B1* and WMC522 associated with *Ppd-A1* (Table 2), emphasizing the presence and importance of these genes in the Central European varieties.

A series of vernalization genes determining the growth habit of wheat, has been described and functionally characterized (Trevaskis et al., 2007; Distelfeld et al., 2009). These include the series of *VRN-1* genes on homeologous chromosomes 5A, 5B, and 5D (Yan et al., 2003), the *Vrn-A2* gene on the distal end of chromosome 5AL (Yan et al., 2004), the *Vrn-B3* gene on chromosome arm 7BS (Yan et al., 2006) and the *Vrn-D4* gene in the centromeric region of chromosome 5D (Yoshida et al., 2010). In winter wheat usually all four genes *Vrn-A1*, *Vrn-B1*, *Vrn-D1*, and *Vrn-B3* are present in recessive state, while the presence of one or more dominant alleles was only detected in spring wheat varieties (Zhang et al., 2008). This assumption did not verify for *Vrn-B1* in our set of varieties, which had a dominant allele for three spring varieties, but also three winter varieties (Buteo, Discus, and Lona). No significant associations were found for this rare *Vrn-B1* allele, which indicated that the highly significant association of SSR markers WMC160 and BARC232 on chromosome 5BL was not caused by LD to *Vrn-B1*, but probably by the presence of another gene. Also on the respective chromosomal locations for *Vrn-A1* on chromosome 5A and *Vrn-B3* on chromosome 7BS no significant SSR markers were detected. The highly significant MTAs detected by marker GWM291 on the distal end of chromosome 5A in all environments and BLUEs coincided with the location of *Vrn-A2*. *Vrn-A2* has been described as floral repressor that delays flowering until plants are vernalized. Loss of function of *Vrn-A2* results in spring types (Yan et al., 2004; Trevaskis et al., 2007). Allele GWM291_176 bp was among the five best markers based on the additive effects (Table 2). The vernalization gene *Vrn-B3* is linked completely to a gene similar to Arabidopsis *FLOWERING LOCUS T* (*FT*). Transcript levels of the barley and wheat orthologs, designated as *HvFt* and *TaFT*, respectively, are significantly higher in plants for the dominant *Vrn3* alleles (early flowering) than in plants homozygous for the recessive *vrn3* alleles (late flowering) (Yan et al., 2006). It was shown that nucleotide polymorphisms on A and D copies of the wheat *FT* gene were associated with variations for HD in a collection of 239 diverse lines (Bonnin et al., 2008). Gene copy *TaFT-7D* was mapped in the region of marker GWM44 in the central region of chromosome 7D (Bonnin et al., 2008). We detected three significant markers (GWM4335, GWM3062, BARC126) located distal to GWM44, which may or may not be in LD with *TaFT-7D*.

Several of our MTAs coincided with published meta-QTL regions for HD (Hanocq et al., 2007; Griffiths et al., 2009). Besides the already described genomic regions on homeologous groups 2 and 5, the marker WMC264 on chromosome 3A detecting multiple MTAs coincided with a meta-QTL described by Griffiths et al. (2009). Two alleles of WMC264 with opposing effects are included in our table of best and worst alleles (Table 2). On chromosome 3B, QTL for HD were described for the genomic region proximal to GWM493 (Pánková et al., 2008; Griffiths et al., 2009), which may coincide with the MTAs detected by WMC808 in our study. The studies of Griffiths et al. (2009) as well as Hanocq et al. (2007) describe QTLs linked to GWM251 on chromosome 4B. Marker GWM4636, which detected multiple MTAs in our study, is the neighboring marker in our map. In the Charger × Badger population a QTL was described in the interval GWM408 to BARC140 on chromosome 5BL. This interval includes WMC160 and BARC232 which detected both highly significant MTAs in multiple environments in our study. We assume that this MTA is independent of *Vrn-B1*, since the candidate markers for *Vrn-B1* were not significant. A second QTL was described by Griffiths et al. on chromosome 5B located in the interval GWM540 to GWM544. This interval includes WMC376 in our map, which detected multiple MTAs. Markers for both QTL regions on chromosome 5B (WMC160 and WMC783) are included in our selected list of markers (Table 2). Marker WMC14 on chromosome 7DL detected both QTL in the studies of Griffiths et al. (2009) and Hanocq et al. (2007) as well as MTAs in our study. A QTL extending distal to GWM44 on chromosome 7DS in the Savannah × Rialto population (Griffiths et al., 2009) coincided with the MTAs detected by markers GWM4335, GWM3062, and BARC126 in our study. On chromosome 1BL, the QTL in the interval WMC44 to BARC80 detected in the Avalon × Cadenza population (Griffiths et al., 2009) coincided with MTAs detected by markers GWM3166 and GWM1364 in our study. The QTL detected in the region of GWM18 on chromosome 1BS (Griffiths et al., 2009) covered BARC240 showing a MTA in our study, however the highly significant GWM1130 further distal seems not to be included in the described meta-QTL region. In the association study of Le Gouis et al. (2012) marker GWM 642 detected an association for HD in non-vernalized plants. This marker is in close vicinity to WMC732 detecting multiple MTAs in our study. The detailed comparison to the other associations described by Le Gouis et al. (2012) is difficult due to the lack of common markers.

### Associations detected with SNPs and exploitation of synteny

The SNP markers on the array are mostly new and therefore no literature data on MTAs involving these markers are available. While the SSR markers are mainly based on genomic sequences, the SNPs were mostly derived from genes and can therefore be used to establish the synteny to rice and other grasses, where full genome sequences are available (International Rice Genome Sequencing Project, 2005; The International Brachypodium Initiative, 2010).

Our results indicated, that a wheat gene on chromosome 5B, which is related to the *Hd6* gene family of rice, has a major impact on heading time in wheat. Several earliness *per se* QTL on chromosome 5B were described in the Cutler × Barrie spring wheat population (Kamran et al., 2013). The earliness *per se* QTL *QFlt.dms-5B.1* inducing earlier flowering could help to elongate the grain filling duration for higher grain yield (Kamran et al., 2013). The SSR marker GWM371 linked to *QFlt.dms-5B.1* is located in some distance from the location of WMC160 and BARC232 according to Ganal and Röder (2007), indicating that *QFlt.dms-5B.1* is different from the *Hd6* related SNP marker association of marker Kukri_c10016_369.

The synteny to rice can also be used to indirectly compare the mapping of our significant wheat markers to published literature data. An example is a cluster of three highly significant wheat SNP markers on chromosome 1AL which was not discovered by SSR markers (Supplemental file 8). On chromosome 1AL the fine mapping of the earliness *per se* gene *Eps-A^m^1* was reported (Valárik et al., 2006; Lewis et al., 2008). After establishing the synteny of rice of our significant loci (LOC_Os05g45930 for wsnp_BE444305A_Td_2_1 and for wsnp_RFL_Contig3542_3718200; LOC_Os05g45900 for RAC875_c21411_162) it was possible to compare to the location of *Eps-A^m^1* established by Valárik et al. (2006) between markers *Adk1* (LOC_Os05g51560) and *Pp2c* (LOC_Os05g51510). Based on the rice syntenic loci our locus appears to be different from gene *Eps-A^m^1*. A similar example exists for chromosome 3A for which the presence of earliness *per se* locus *Eps-3A^m^* was reported (Gawronski and Schnurbusch, 2012). The syntenic locus of the significant wheat marker wsnp_ex_c8884_14841846 (LOC_Os01g64490) in our map did not match the location of the markers PAV_295_296 (LOC_Os01g740300), CAPS_zt4_zt5 (LOC_Os01g741100) and CAPS_281_282 (LOC_Os01g741400) reported to be linked to *Eps-3A^m^* (Gawronski and Schnurbusch, 2012).

In barley, the circadian clock gene *early maturity 8* (*eam8*) was identified as an ortholog of the *Arabidopsis thaliana* circadian clock regulator *early flowering* (*elf3*) (Faure et al., 2012; Zakhrabekova et al., 2012). The reported syntenic region in rice, ranging from LOC_Os5g51560 to LOC_Os05g51650 did not include any significant markers in our list, for which synteny to rice could be established. For the barley *early maturity 10* (*eam10*) gene the *Hvlux1* gene, an ortholog to the *Arabidopsis* circadian gene *LUX ARRHYTHMO*, was proposed as a candidate (Campoli et al., 2013) with orthologs in rice (LOC_Os01g74020) and *Brachypodium* (Bradi2g62070). For none of these orthologous sites candidates were found in our wheat association panel.

## Conclusions

Genome wide associations for HD in European winter wheat were established for SSR as well as SNP markers. It could be shown that a number of known regulatory photoperiodism genes, such as *Ppd-A1*, *Ppd-B1*, *Ppd-D1* and the vernalization gene *Vrn-A2* have a major impact in shaping the genetic architecture of HD. The distribution of MTAs in multiple environments led however to the conclusion, that many more major genetic loci are involved. We were able to demonstrate the significance of an *Hd6* related gene marker on chromosome 5BL, which indicated the importance of the *Hd6* related gene for HD in wheat.

The dependence of the number of favorable alleles of SSR markers in a variety in relation to the HD-BLUEs indicated the strong genetic component in HD. By considering only five markers, it was possible to obtain a regression with *R*^2^ = 0.372. Therefore, the described list of markers (Table 2) could be used for the stacking of alleles by marker assisted breeding and for the development of well adapted varieties for specific environments and geographical locations.

### Conflict of interest statement

Sonja Kollers, Viktor Korzun, and Erhard Ebmeyer are employed by the company KWS LOCHOW GMBH, Odile Argillier, Gunther Stiewe, and Maike Hinze are employed by Syngenta Seeds GmbH and Martin W. Ganal, Jörg Plieske are employed by the company TraitGenetics GmbH. The companies have commercial interest in the results for application in variety development and for the provision of genotyping services. This does not alter the authors' adherence to all Frontiers policies on sharing data and materials. The authors declare that the research was conducted in the absence of any commercial or financial relationships that could be construed as a potential conflict of interest.
