# Supplementary material for: Genetic architecture of main effect QTL for heading date in European winter wheat
Source: Front Plant Sci. 2014 May 20;5:217. doi: 10.3389/fpls.2014.00217 (PMC4033046; doi:10.3389/fpls.2014.00217)
Supplement: Supplementary file 2 [file DataSheet2.DOCX]

**Table S2: Spearman rank order correlation of HD scores in 372 varieties among eight environments and the BLUEs. ***** P<0.001

|  | 2009.SEL | 2009.WOH | 2010.AND | 2010.JAN | 2010.SAU | 2010.SEL | 2010.WOH | BLUEs |
| --- | --- | --- | --- | --- | --- | --- | --- | --- |
| 2009.AND | 0.901*** | 0.916*** | 0.926*** | 0.929*** | 0.925*** | 0.852*** | 0.903*** | 0.960*** |
| 2009.SEL |  | 0.942*** | 0.881*** | 0.903*** | 0.897*** | 0.910*** | 0.900*** | 0.952*** |
| 2009.WOH |  |  | 0.924*** | 0.931*** | 0.939*** | 0.898*** | 0.926*** | 0.973*** |
| 2010.AND |  |  |  | 0.935*** | 0.956*** | 0.843*** | 0.906*** | 0.964*** |
| 2010.JAN |  |  |  |  | 0.939*** | 0.871*** | 0.898*** | 0.968*** |
| 2010.SAU |  |  |  |  |  | 0.861*** | 0.923*** | 0.971*** |
| 2010.SEL |  |  |  |  |  |  | 0.881*** | 0.915*** |
| 2010.WOH |  |  |  |  |  |  |  | 0.950*** |
