# Supplementary material for: Genetic architecture of main effect QTL for heading date in European winter wheat
Source: Front Plant Sci. 2014 May 20;5:217. doi: 10.3389/fpls.2014.00217 (PMC4033046; doi:10.3389/fpls.2014.00217)
Supplement: Supplementary file 3 [file DataSheet3.DOCX]

**Table S3: Analysis of variance (ANOVA) of HD score in 372 varieties in eight environments.**

|  | DF | SS | MS | F | P |
| --- | --- | --- | --- | --- | --- |
| Genotype | 371 | 29824 | 80 | 59.5 | < 0.001******* |
| Environment | 7 | 78333 | 11191 | 8276.4 | < 0.001******* |
| Residual | 2597 | 3511 | 1 |  |  |
| Total | 2975 | 111668 | 37 |  |  |

DF = Degrees of freedom

SS = Sum of Squares

MS = Mean of Squares

******* P<0.001
