# Supplementary material for: Genetic architecture of main effect QTL for heading date in European winter wheat
Source: Front Plant Sci. 2014 May 20;5:217. doi: 10.3389/fpls.2014.00217 (PMC4033046; doi:10.3389/fpls.2014.00217)
Supplement: Supplementary file 4 [file DataSheet4.DOCX]

Supplemental File 4: Estimation of variance components and broad sense heritability and estimation of differences between groups (= environments) using ANOVA and a Tukey B test.

| **Variance Estimates** | | | | | | | |  | | | | | |  | | |  | | |  |  |  |
| --- | --- | --- | --- | --- | --- | --- | --- | --- | --- | --- | --- | --- | --- | --- | --- | --- | --- | --- | --- | --- | --- | --- |
| Component | | | Estimate | | | | |  | | | | | |  | | |  | | |  |  |  |
| Var(genotype) | | | 6,120 | | | | |  | | | | | |  | | |  | | |  |  |  |
| Var(Error) | | | 31,430 | | | | | **0,609** | | | | | | **H^2^** | | |  | | |  |  |  |
| Dependent Variable: trait Method: Minimum Norm Quadratic Unbiased Estimation (Weight = 1 for Random Effects and Residual) | | | | | | | |  | | | | | |  | | |  | | |  |  |  |
|  | | | | |  | | |  | | | | | |  | | |  | | |  |  |  |
|  | | | | |  | | |  | | | | | |  | | |  | | |  |  |  |
| **ANOVA** | | | | | | | | | | | | | | | | | | |  | | |  |
| trait | | | | | | | | | | | | | | | | | | |  | | |  |
|  | | Sum of Squares | | | | | df | | | | Mean Square | | F | | | Sig. | | |  | | |  |
| Between Groups | | 78333,125 | | | | | 7 | | | | 11190,446 | | 996,332 | | | ,000 | | |  | | |  |
| Within Groups | | 33335,532 | | | | | 2968 | | | | 11,232 | |  | | |  | | |  | | |  |
| Total | | 111668,657 | | | | | 2975 | | | |  | |  | | |  | | |  | | |  |
|  | |  | | | | |  | | | |  | |  | | |  | | |  | | |  |
| **Trait Posthoc test** | | | | | | | | | | | | | | | | | |  |  |  |  |  |
| Tukey B^a^ | | | | | | | | | | | | | | | | | |  |  |  |  |  |
| env no | N | | | Subset for alpha = 0.05 | | | | | | | | | | | | | |  |  |  |  |  |
|  |  |  |  | 1 | | 2 | | | 3 | 4 | | 5 | | | 6 | | |  |  |  |  |  |
| 09.WOH.HD | 372 | | | 144,483333 | |  | | |  |  | |  | | |  | | |  |  |  |  |  |
| 10.AND.HD | 372 | | |  | | 148,070430 | | |  |  | |  | | |  | | |  |  |  |  |  |
| 09.AND.HD | 372 | | |  | | 148,184140 | | |  |  | |  | | |  | | |  |  |  |  |  |
| 10.JAN.HD | 372 | | |  | |  | | | 149,756989 |  | |  | | |  | | |  |  |  |  |  |
| 09.SEL.HD | 372 | | |  | |  | | | 149,965054 |  | |  | | |  | | |  |  |  |  |  |
| 10.SAU.HD | 372 | | |  | |  | | |  | 152,256183 | |  | | |  | | |  |  |  |  |  |
| 10.SEL.HD | 372 | | |  | |  | | |  |  | | 157,209946 | | |  | | |  |  |  |  |  |
| 10.WOH.HD | 372 | | |  | |  | | |  |  | |  | | | 161,502688 | | |  |  |  |  |  |
| Means for groups in homogeneous subsets are displayed. | | | | | | | | | | | | | | | | | |  |  |  |  |  |
| a. Uses Harmonic Mean Sample Size = 372,000. | | | | | | | | | | | | | | | | | |  |  |  |  |  |
