# Supplementary material for: Genetic architecture of main effect QTL for heading date in European winter wheat
Source: Front Plant Sci. 2014 May 20;5:217. doi: 10.3389/fpls.2014.00217 (PMC4033046; doi:10.3389/fpls.2014.00217)
Supplement: Supplementary file 8 [file DataSheet8.PDF]

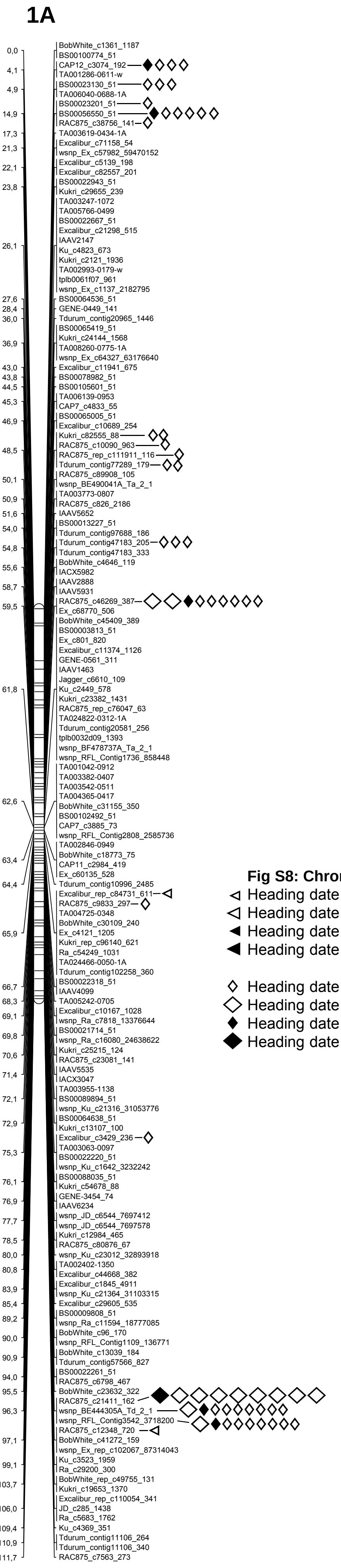

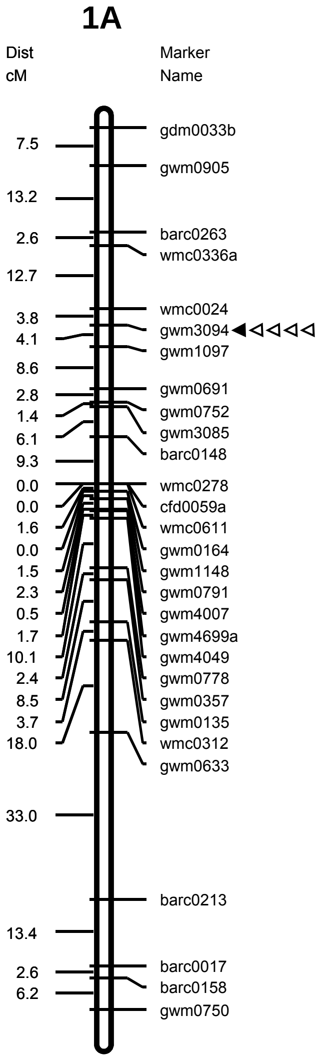

Fig S8: Chromosomal location of marker trait associations.

◁ Heading date decreasing effect, single environment, -log10(p)-value ≥ 3.0
◁ Heading date decreasing effect, single environment, -log10(p)-value ≥ 4.82 (SSR) ≥ 5.89 (SNP)
◀ Heading date decreasing effect, BLUES-value, -log10(p)-value ≥ 3.0
◀ Heading date decreasing effect, BLUES-value, -log10(p)-value ≥ 4.82 (SSR) ≥ 5.89 (SNP)

◇ Heading date increasing effect, single environment, -log10(p)-value ≥ 3.0
◇ Heading date increasing effect, single environment, -log10(p)-value ≥ 4.82 (SSR) ≥ 5.89 (SNP)
◆ Heading date increasing effect, BLUES-value, -log10(p)-value ≥ 3.0
◆ Heading date increasing effect, BLUES-value, -log10(p)-value ≥ 4.82 (SSR) ≥ 5.89 (SNP)

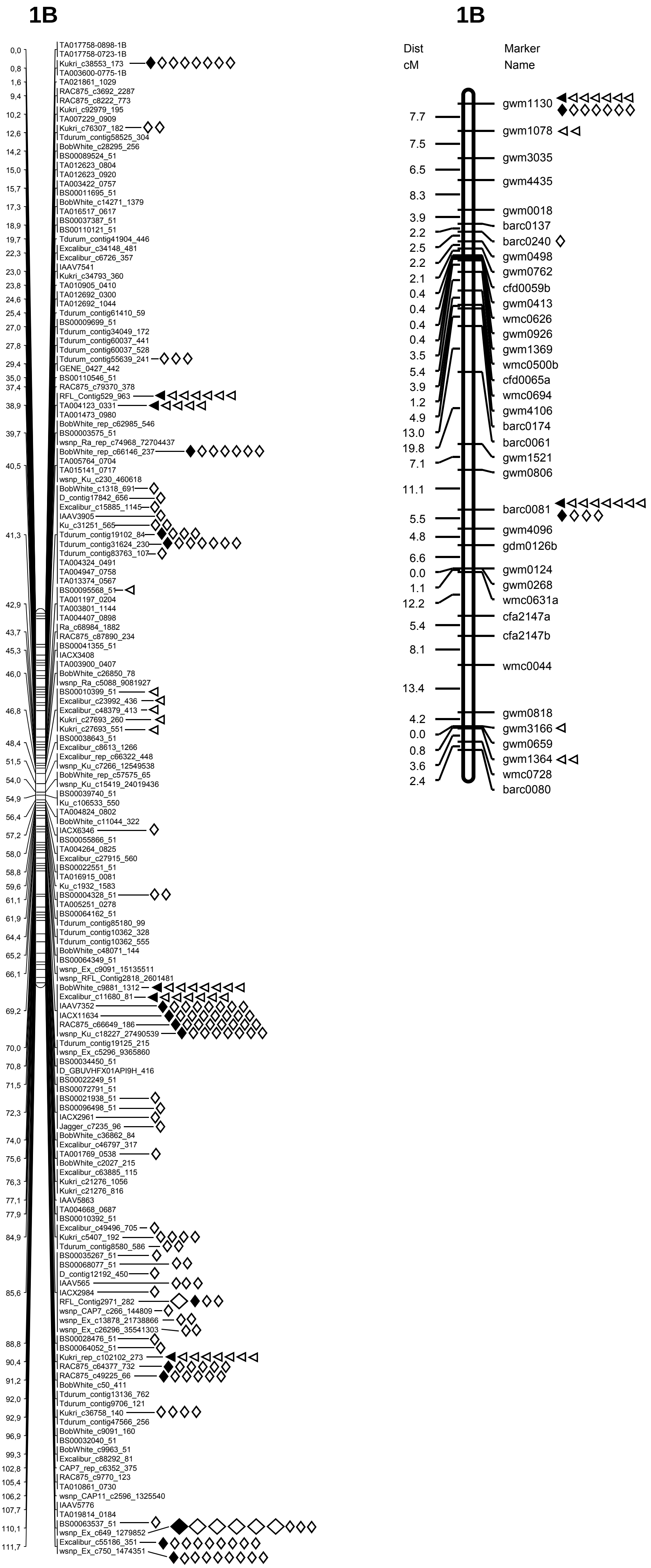
1B
0,0
0,8
1,6
9,4
10,2
12,6
14,2
15,0
15,7
17,3
18,9
19,7
22,3
23,0
23,8
24,6
25,4
27,0
27,8
29,4
35,0
37,4
38,9
39,7
40,5
41,3
42,9
43,7
45,3
46,0
46,8
48,4
51,5
54,0
54,9
56,4
57,2
58,0
58,8
59,6
61,1
61,9
64,4
65,2
66,1
69,2
70,0
70,8
71,5
72,3
74,0
75,6
76,3
77,1
77,9
84,9
85,6
88,8
90,4
91,2
92,0
92,9
96,9
99,3
102,8
105,4
106,2
107,7
110,1
111,7
TA017758-0898-1B
TA017758-0723-1B
Kukri_c38553_173
TA003600-0775-1B
TA021861_1029
RAC875_c3692_2287
RAC875_c8222_773
Kukri_c92979_195
TA007229_0909
Kukri_c76307_182
Tdurum_contig58525_304
BobWhite_c28295_256
BS00089524_51
TA012623_0804
TA012623_0920
TA003422_0757
BS00011695_51
BobWhite_c14271_1379
TA016517_0617
BS00037387_51
BS00110121_51
Tdurum_contig41904_446
Excalibur_c34148_481
Excalibur_c6726_357
IAAV7541
Kukri_c34793_360
TA010905_0410
TA012692_0300
TA012692_1044
Tdurum_contig61410_59
BS00009699_51
Tdurum_contig34049_172
Tdurum_contig60037_441
Tdurum_contig60037_528
Tdurum_contig55639_241
GENE_0427_442
BS00110546_51
RAC875_c79370_378
RFL_Contig529_963
TA004123_0331
TA001473_0980
BobWhite_rep_c62985_546
BS00003575_51
wsnp_Ra_rep_c74968_72704437
BobWhite_rep_c66146_237
TA005764_0704
TA015141_0717
wsnp_Ku_c230_460618
BobWhite_c1318_691
D_contig17842_656
Excalibur_c15885_1145
IAAV3905
Ku_c31251_565
Tdurum_contig19102_84
Tdurum_contig31624_230
Tdurum_contig83763_107
TA004324_0491
TA004947_0758
TA013374_0567
BS00095568_51
TA001197_0204
TA003801_1144
TA004407_0898
Ra_c68984_1882
RAC875_c87890_234
BS00041355_51
IACX3408
TA003900_0407
BobWhite_c26850_78
wsnp_Ra_c5088_9081927
BS00010399_51
Excalibur_c23992_436
Excalibur_c48379_413
Kukri_c27693_260
Kukri_c27693_551
BS00038643_51
Excalibur_c8613_1266
Excalibur_rep_c66322_448
wsnp_Ku_c7266_12549538
BobWhite_rep_c57575_65
wsnp_Ku_c15419_24019436
BS00039740_51
Ku_c106533_550
TA004824_0802
BobWhite_c11044_322
IACX6346
BS00055866_51
TA004264_0825
Excalibur_c27915_560
BS00022551_51
TA016915_0081
Ku_c1932_1583
BS00004328_51
TA005251_0278
BS00064162_51
Tdurum_contig85180_99
Tdurum_contig10362_328
Tdurum_contig10362_555
BobWhite_c48071_144
BS00064349_51
wsnp_Ex_c9091_15135511
wsnp_RFL_Contig2818_2601481
BobWhite_c9881_1312
Excalibur_c11680_81
IAAV7352
IACX11634
RAC875_c66649_186
wsnp_Ku_c18227_27490539
Tdurum_contig19125_215
wsnp_Ex_c5296_9365860
BS00034450_51
D_GBUVHFX01API9H_416
BS00022249_51
BS00072791_51
BS00021938_51
BS00096498_51
IACX2961
Jagger_c7235_96
BobWhite_c36862_84
Excalibur_c46797_317
TA001769_0538
BobWhite_c2027_215
Excalibur_c63885_115
Kukri_c21276_1056
Kukri_c21276_816
IAAV5863
TA004668_0687
BS00010392_51
Excalibur_c49496_705
Kukri_c5407_192
Tdurum_contig8580_586
BS00035267_51
BS00068077_51
D_contig12192_450
IAAV565
IACX2984
RFL_Contig2971_282
wsnp_CAP7_c266_144809
wsnp_Ex_c13878_21738866
wsnp_Ex_c26296_35541303
BS00028476_51
BS00064052_51
Kukri_rep_c102102_273
RAC875_c64377_732
RAC875_c49225_66
BobWhite_c50_411
Tdurum_contig13136_762
Tdurum_contig9706_121
Kukri_c36758_140
Tdurum_contig47566_256
BobWhite_c9091_160
BS00032040_51
BobWhite_c9963_51
Excalibur_c88292_81
CAP7_rep_c6352_375
RAC875_c9770_123
TA010861_0730
wsnp_CAP11_c2596_1325540
IAAV5776
TA019814_0184
BS00063537_51
wsnp_Ex_c649_1279852
Excalibur_c55186_351
wsnp_Ex_c750_1474351
1B
Dist
cM
Marker
Name
7.7
7.5
6.5
8.3
3.9
2.2
2.5
2.2
2.1
0.4
0.4
0.4
0.4
3.5
5.4
3.9
1.2
4.9
13.0
19.8
7.1
11.1
5.5
4.8
6.6
0.0
1.1
12.2
5.4
8.1
13.4
4.2
0.0
0.8
3.6
2.4
gwm1130
gwm1078
gwm3035
gwm4435
gwm0018
barc0137
barc0240
gwm0498
gwm0762
cfd0059b
gwm0413
wmc0626
gwm0926
gwm1369
wmc0500b
cfd0065a
wmc0694
gwm4106
barc0174
barc0061
gwm1521
gwm0806
barc0081
gwm4096
gdm0126b
gwm0124
gwm0268
wmc0631a
cfa2147a
cfa2147b
wmc0044
gwm0818
gwm3166
gwm0659
gwm1364
wmc0728
barc0080

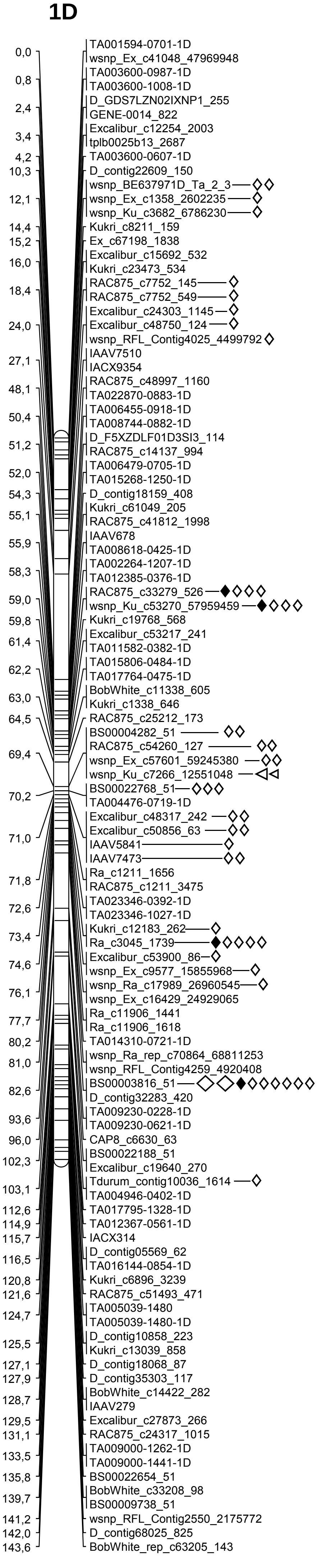
1D
0,0
0,8
2,4
3,4
4,2
10,3
12,1
14,4
15,2
16,0
18,4
24,0
27,1
48,1
50,4
51,2
52,0
54,3
55,1
55,9
58,3
59,0
59,8
61,4
62,2
63,0
64,5
69,4
70,2
71,0
71,8
72,6
73,4
74,6
76,1
77,7
80,2
81,0
82,6
93,6
96,0
102,3
103,1
112,6
114,9
115,7
116,5
120,8
121,6
124,7
125,5
127,1
127,9
128,7
129,5
131,1
133,5
135,8
139,7
141,2
142,0
143,6
TA001594-0701-1D
wsnp_Ex_c41048_47969948
TA003600-0987-1D
TA003600-1008-1D
D_GDS7LZN02IXNP1_255
GENE-0014_822
Excalibur_c12254_2003
tplb0025b13_2687
TA003600-0607-1D
D_contig22609_150
wsnp_BE637971D_Ta_2_3
wsnp_Ex_c1358_2602235
wsnp_Ku_c3682_6786230
Kukri_c8211_159
Ex_c67198_1838
Excalibur_c15692_532
Kukri_c23473_534
RAC875_c7752_145
RAC875_c7752_549
Excalibur_c24303_1145
Excalibur_c48750_124
wsnp_RFL_Contig4025_4499792
IAAV7510
IACX9354
RAC875_c48997_1160
TA022870-0883-1D
TA006455-0918-1D
TA008744-0882-1D
D_F5XZDLF01D3SI3_114
RAC875_c14137_994
TA006479-0705-1D
TA015268-1250-1D
D_contig18159_408
Kukri_c61049_205
RAC875_c41812_1998
IAAV678
TA008618-0425-1D
TA002264-1207-1D
TA012385-0376-1D
RAC875_c33279_526
wsnp_Ku_c53270_57959459
Kukri_c19768_568
Excalibur_c53217_241
TA011582-0382-1D
TA015806-0484-1D
TA017764-0475-1D
BobWhite_c11338_605
Kukri_c1338_646
RAC875_c25212_173
BS00004282_51
RAC875_c54260_127
wsnp_Ex_c57601_59245380
wsnp_Ku_c7266_12551048
BS00022768_51
TA004476-0719-1D
Excalibur_c48317_242
Excalibur_c50856_63
IAAV5841
IAAV7473
Ra_c1211_1656
RAC875_c1211_3475
TA023346-0392-1D
TA023346-1027-1D
Kukri_c12183_262
Ra_c3045_1739
Excalibur_c53900_86
wsnp_Ex_c9577_15855968
wsnp_Ra_c17989_26960545
wsnp_Ex_c16429_24929065
Ra_c11906_1441
Ra_c11906_1618
TA014310-0721-1D
wsnp_Ra_rep_c70864_68811253
wsnp_RFL_Contig4259_4920408
BS00003816_51
D_contig32283_420
TA009230-0228-1D
TA009230-0621-1D
CAP8_c6630_63
BS00022188_51
Excalibur_c19640_270
Tdurum_contig10036_1614
TA004946-0402-1D
TA017795-1328-1D
TA012367-0561-1D
IACX314
D_contig05569_62
TA016144-0854-1D
Kukri_c6896_3239
RAC875_c51493_471
TA005039-1480
TA005039-1480-1D
D_contig10858_223
Kukri_c13039_858
D_contig18068_87
D_contig35303_117
BobWhite_c14422_282
IAAV279
Excalibur_c27873_266
RAC875_c24317_1015
TA009000-1262-1D
TA009000-1441-1D
BS00022654_51
BobWhite_c33208_98
BS00009738_51
wsnp_RFL_Contig2550_2175772
D_contig68025_825
BobWhite_rep_c63205_143

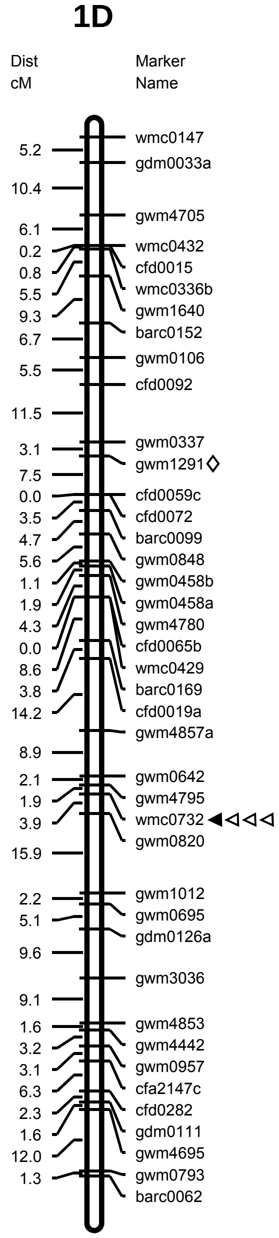
1D
Dist
cM
Marker
Name
5.2
10.4
6.1
0.2
0.8
5.5
9.3
6.7
5.5
11.5
3.1
7.5
0.0
3.5
4.7
5.6
1.1
1.9
4.3
0.0
8.6
3.8
14.2
8.9
2.1
1.9
3.9
15.9
2.2
5.1
9.6
9.1
1.6
3.2
3.1
6.3
2.3
1.6
12.0
1.3
wmc0147
gdm0033a
gwm4705
wmc0432
cfd0015
wmc0336b
gwm1640
barc0152
gwm0106
cfd0092
gwm0337
gwm1291
cfd0059c
cfd0072
barc0099
gwm0848
gwm0458b
gwm0458a
gwm4780
cfd0065b
wmc0429
barc0169
cfd0019a
gwm4857a
gwm0642
gwm4795
wmc0732
gwm0820
gwm1012
gwm0695
gdm0126a
gwm3036
gwm4853
gwm4442
gwm0957
cfa2147c
cfd0282
gdm0111
gwm4695
gwm0793
barc0062

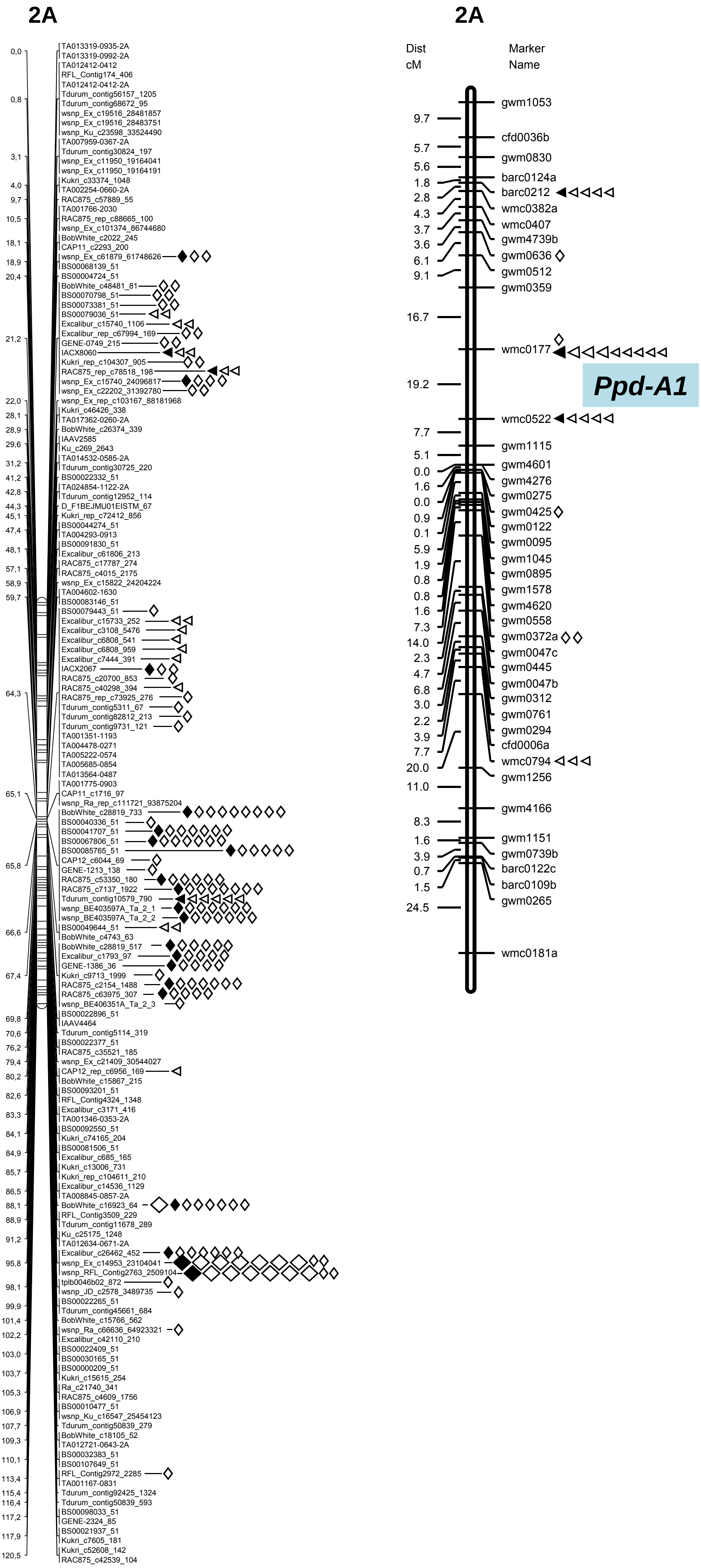
2A
0,0
0,8
3,1
4,0
9,7
10,5
18,1
18,9
20,4
21,2
22,0
28,1
28,9
29,6
31,2
41,2
42,8
44,3
45,1
47,4
48,1
57,1
58,9
59,7
64,3
65,1
65,8
66,6
67,4
69,8
70,6
76,2
79,4
80,2
82,6
83,3
84,1
84,9
85,7
86,5
88,1
88,9
91,2
95,8
98,1
99,9
101,4
102,2
103,0
103,7
105,3
106,9
107,7
109,3
110,1
113,4
115,4
116,4
117,2
117,9
120,5
TA013319-0935-2A
TA013319-0992-2A
TA012412-0412
RFL_Contig174_406
TA012412-0412-2A
Tdurum_contig56157_1205
Tdurum_contig68672_95
wsnp_Ex_c19516_28481857
wsnp_Ex_c19516_28483751
wsnp_Ku_c23598_33524490
TA007959-0367-2A
Tdurum_contig30824_197
wsnp_Ex_c11950_19164041
wsnp_Ex_c11950_19164191
Kukri_c33374_1048
TA002254-0660-2A
RAC875_c57889_55
TA001766-2030
RAC875_rep_c88665_100
wsnp_Ex_c101374_86744680
BobWhite_c2022_245
CAP11_c2293_200
wsnp_Ex_c61879_61748626
BS00068139_51
BS00004724_51
BobWhite_c48481_81
BS00070798_51
BS00073381_51
BS00079036_51
Excalibur_c15740_1106
Excalibur_rep_c67994_169
GENE-0749_215
IACX8060
Kukri_rep_c104307_905
RAC875_rep_c78518_198
wsnp_Ex_c15740_24096817
wsnp_Ex_c22202_31392780
wsnp_Ex_rep_c103167_88181968
Kukri_c46426_338
TA017362-0260-2A
BobWhite_c26374_339
IAAV2585
Ku_c269_2643
TA014532-0585-2A
Tdurum_contig30725_220
BS00022332_51
TA024854-1122-2A
Tdurum_contig12952_114
D_F1BEJMU01EISTM_67
Kukri_rep_c72412_856
BS00044274_51
TA004293-0913
BS00091830_51
Excalibur_c61806_213
RAC875_c17787_274
RAC875_c4015_2175
wsnp_Ex_c15822_24204224
TA004602-1630
BS00083146_51
BS00079443_51
Excalibur_c15733_252
Excalibur_c3108_5476
Excalibur_c6808_541
Excalibur_c6808_959
Excalibur_c7444_391
IACX2067
RAC875_c20700_853
RAC875_c40298_394
RAC875_rep_c73925_276
Tdurum_contig5311_67
Tdurum_contig82812_213
Tdurum_contig9731_121
TA001351-1193
TA004478-0271
TA005222-0574
TA005685-0854
TA013564-0487
TA001775-0903
CAP11_c1716_97
wsnp_Ra_rep_c111721_93875204
BobWhite_c28819_733
BS00040336_51
BS00041707_51
BS00067806_51
BS00085765_51
CAP12_c6044_69
GENE-1213_138
RAC875_c53350_180
RAC875_c7137_1922
Tdurum_contig10579_790
wsnp_BE403597A_Ta_2_1
wsnp_BE403597A_Ta_2_2
BS00049644_51
BobWhite_c4743_63
BobWhite_c28819_517
Excalibur_c1793_97
GENE-1386_36
Kukri_c9713_1999
RAC875_c2154_1488
RAC875_c63975_307
wsnp_BE406351A_Ta_2_3
BS00022896_51
IAAV4464
Tdurum_contig5114_319
BS00022377_51
RAC875_c35521_185
wsnp_Ex_c21409_30544027
CAP12_rep_c6956_169
BobWhite_c15867_215
BS00093201_51
RFL_Contig4324_1348
Excalibur_c3171_416
TA001346-0353-2A
BS00092550_51
Kukri_c74165_204
BS00081506_51
Excalibur_c685_165
Kukri_c13006_731
Kukri_rep_c104611_210
Excalibur_c14536_1129
TA008845-0857-2A
BobWhite_c16923_64
RFL_Contig3509_229
Tdurum_contig11678_289
Ku_c25175_1248
TA012634-0671-2A
Excalibur_c26462_452
wsnp_Ex_c14953_23104041
wsnp_RFL_Contig2763_2509104
tplb0046b02_872
wsnp_JD_c2578_3489735
BS00022265_51
Tdurum_contig45661_684
BobWhite_c15766_562
wsnp_Ra_c66636_64923321
Excalibur_c42110_210
BS00022409_51
BS00030165_51
BS00000209_51
Kukri_c15615_254
Ra_c21740_341
RAC875_c4609_1756
BS00010477_51
wsnp_Ku_c16547_25454123
Tdurum_contig50839_279
BobWhite_c18105_52
TA012721-0643-2A
BS00032383_51
BS00107649_51
RFL_Contig2972_2285
TA001167-0831
Tdurum_contig92425_1324
Tdurum_contig50839_593
BS00098033_51
GENE-2324_85
BS00021937_51
Kukri_c7605_181
Kukri_c52608_142
RAC875_c42539_104
2A
Dist
cM
Marker
Name
9.7
5.7
5.6
1.8
2.8
4.3
3.7
3.6
6.1
9.1
16.7
19.2
7.7
5.1
0.0
1.6
0.0
0.9
0.1
5.9
1.9
0.8
0.8
1.6
7.3
14.0
2.3
4.7
6.8
3.0
2.2
3.9
7.7
20.0
11.0
8.3
1.6
3.9
0.7
1.5
24.5
gwm1053
cfd0036b
gwm0830
barc0124a
barc0212
wmc0382a
wmc0407
gwm4739b
gwm0636
gwm0512
gwm0359
wmc0177
Ppd-A1
wmc0522
gwm1115
gwm4601
gwm4276
gwm0275
gwm0425
gwm0122
gwm0095
gwm1045
gwm0895
gwm1578
gwm4620
gwm0558
gwm0372a
gwm0047c
gwm0445
gwm0047b
gwm0312
gwm0761
gwm0294
cfd0006a
wmc0794
gwm1256
gwm4166
gwm1151
gwm0739b
barc0122c
barc0109b
gwm0265
wmc0181a

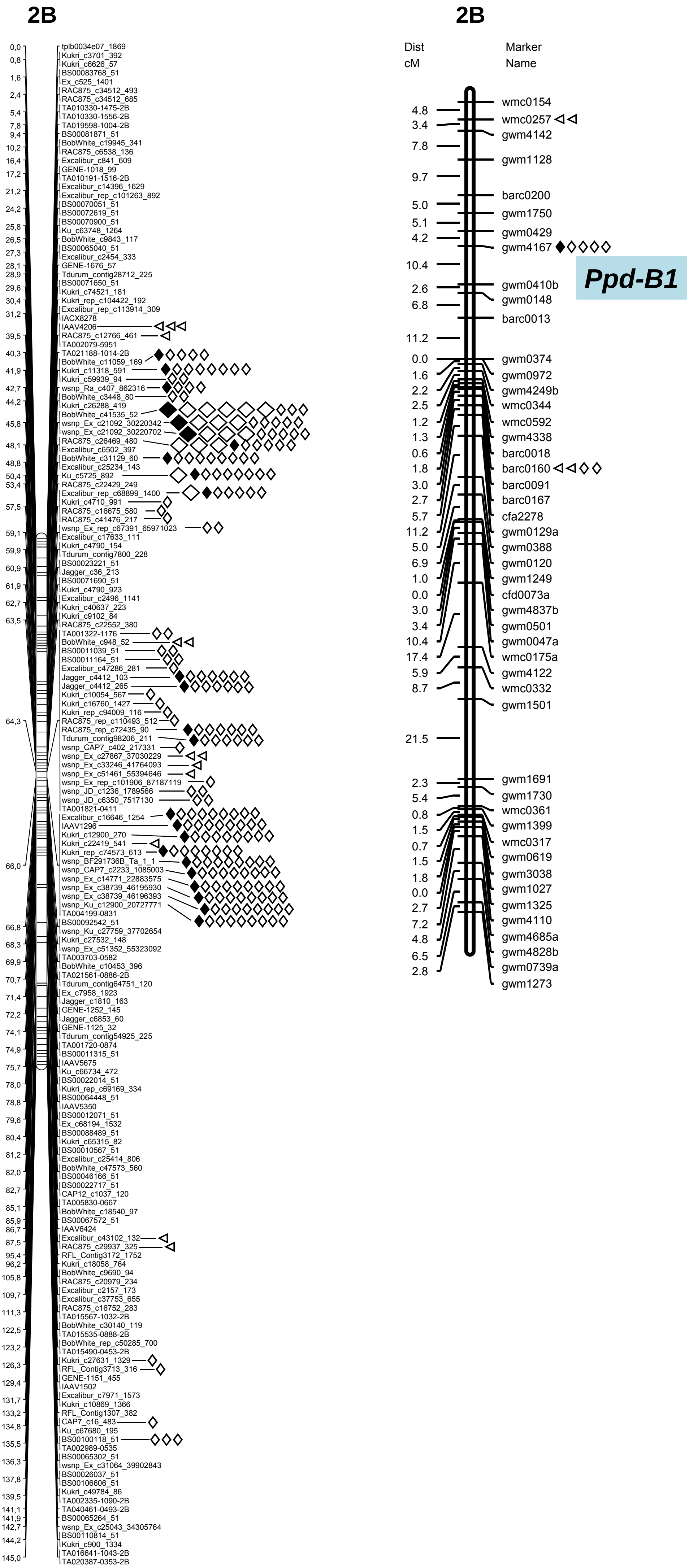
2B
2B
Dist
cM
Marker
Name
Ppd-B1

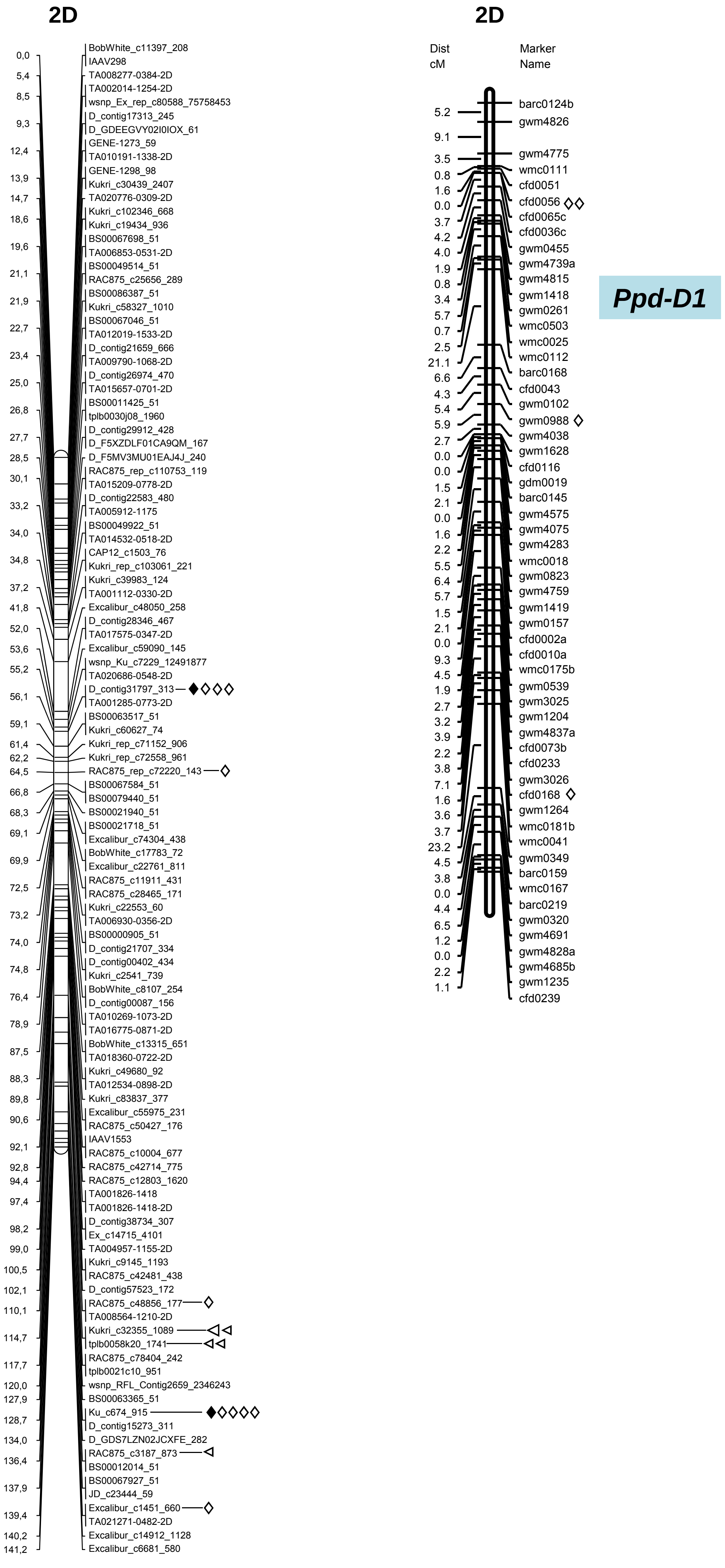
2D
0,0
5,4
8,5
9,3
12,4
13,9
14,7
18,6
19,6
21,1
21,9
22,7
23,4
25,0
26,8
27,7
28,5
30,1
33,2
34,0
34,8
37,2
41,8
52,0
53,6
55,2
56,1
59,1
61,4
62,2
64,5
66,8
68,3
69,1
69,9
72,5
73,2
74,0
74,8
76,4
78,9
87,5
88,3
89,8
90,6
92,1
92,8
94,4
97,4
98,2
99,0
100,5
102,1
110,1
114,7
117,7
120,0
127,9
128,7
134,0
136,4
137,9
139,4
140,2
141,2
BobWhite_c11397_208
IAAV298
TA008277-0384-2D
TA002014-1254-2D
wsnp_Ex_rep_c80588_75758453
D_contig17313_245
D_GDEEGVY02I0IOX_61
GENE-1273_59
TA010191-1338-2D
GENE-1298_98
Kukri_c30439_2407
TA020776-0309-2D
Kukri_c102346_668
Kukri_c19434_936
BS00067698_51
TA006853-0531-2D
BS00049514_51
RAC875_c25656_289
BS00086387_51
Kukri_c58327_1010
BS00067046_51
TA012019-1533-2D
D_contig21659_666
TA009790-1068-2D
D_contig26974_470
TA015657-0701-2D
BS00011425_51
tplb0030j08_1960
D_contig29912_428
D_F5XZDLF01CA9QM_167
D_F5MV3MU01EAJ4J_240
RAC875_rep_c110753_119
TA015209-0778-2D
D_contig22583_480
TA005912-1175
BS00049922_51
TA014532-0518-2D
CAP12_c1503_76
Kukri_rep_c103061_221
Kukri_c39983_124
TA001112-0330-2D
Excalibur_c48050_258
D_contig28346_467
TA017575-0347-2D
Excalibur_c59090_145
wsnp_Ku_c7229_12491877
TA020686-0548-2D
D_contig31797_313
TA001285-0773-2D
BS00063517_51
Kukri_c60627_74
Kukri_rep_c71152_906
Kukri_rep_c72558_961
RAC875_rep_c72220_143
BS00067584_51
BS00079440_51
BS00021940_51
BS00021718_51
Excalibur_c74304_438
BobWhite_c17783_72
Excalibur_c22761_811
RAC875_c11911_431
RAC875_c28465_171
Kukri_c22553_60
TA006930-0356-2D
BS00000905_51
D_contig21707_334
D_contig00402_434
Kukri_c2541_739
BobWhite_c8107_254
D_contig00087_156
TA010269-1073-2D
TA016775-0871-2D
BobWhite_c13315_651
TA018360-0722-2D
Kukri_c49680_92
TA012534-0898-2D
Kukri_c83837_377
Excalibur_c55975_231
RAC875_c50427_176
IAAV1553
RAC875_c10004_677
RAC875_c42714_775
RAC875_c12803_1620
TA001826-1418
TA001826-1418-2D
D_contig38734_307
Ex_c14715_4101
TA004957-1155-2D
Kukri_c9145_1193
RAC875_c42481_438
D_contig57523_172
RAC875_c48856_177
TA008564-1210-2D
Kukri_c32355_1089
tplb0058k20_1741
RAC875_c78404_242
tplb0021c10_951
wsnp_RFL_Contig2659_2346243
BS00063365_51
Ku_c674_915
D_contig15273_311
D_GDS7LZN02JCXFE_282
RAC875_c3187_873
BS00012014_51
BS00067927_51
JD_c23444_59
Excalibur_c1451_660
TA021271-0482-2D
Excalibur_c14912_1128
Excalibur_c6681_580
2D
Dist cM
Marker Name
5.2
9.1
3.5
0.8
1.6
0.0
3.7
4.2
4.0
1.9
0.8
3.4
5.7
0.7
2.5
21.1
6.6
4.3
5.4
5.9
2.7
0.0
0.0
1.5
2.1
0.0
1.6
2.2
5.5
6.4
5.7
1.5
2.1
0.0
9.3
4.5
1.9
2.7
3.2
3.9
2.2
3.8
7.1
1.6
3.6
3.7
23.2
4.5
3.8
0.0
4.4
6.5
1.2
0.0
2.2
1.1
barc0124b
gwm4826
gwm4775
wmc0111
cfd0051
cfd0056
cfd0065c
cfd0036c
gwm0455
gwm4739a
gwm4815
gwm1418
gwm0261
wmc0503
wmc0025
wmc0112
barc0168
cfd0043
gwm0102
gwm0988
gwm4038
gwm1628
cfd0116
gdm0019
barc0145
gwm4575
gwm4075
gwm4283
wmc0018
gwm0823
gwm4759
gwm1419
gwm0157
cfd0002a
cfd0010a
wmc0175b
gwm0539
gwm3025
gwm1204
gwm4837a
cfd0073b
cfd0233
gwm3026
cfd0168
gwm1264
wmc0181b
wmc0041
gwm0349
barc0159
wmc0167
barc0219
gwm0320
gwm4691
gwm4828a
gwm4685b
gwm1235
cfd0239
Ppd-D1

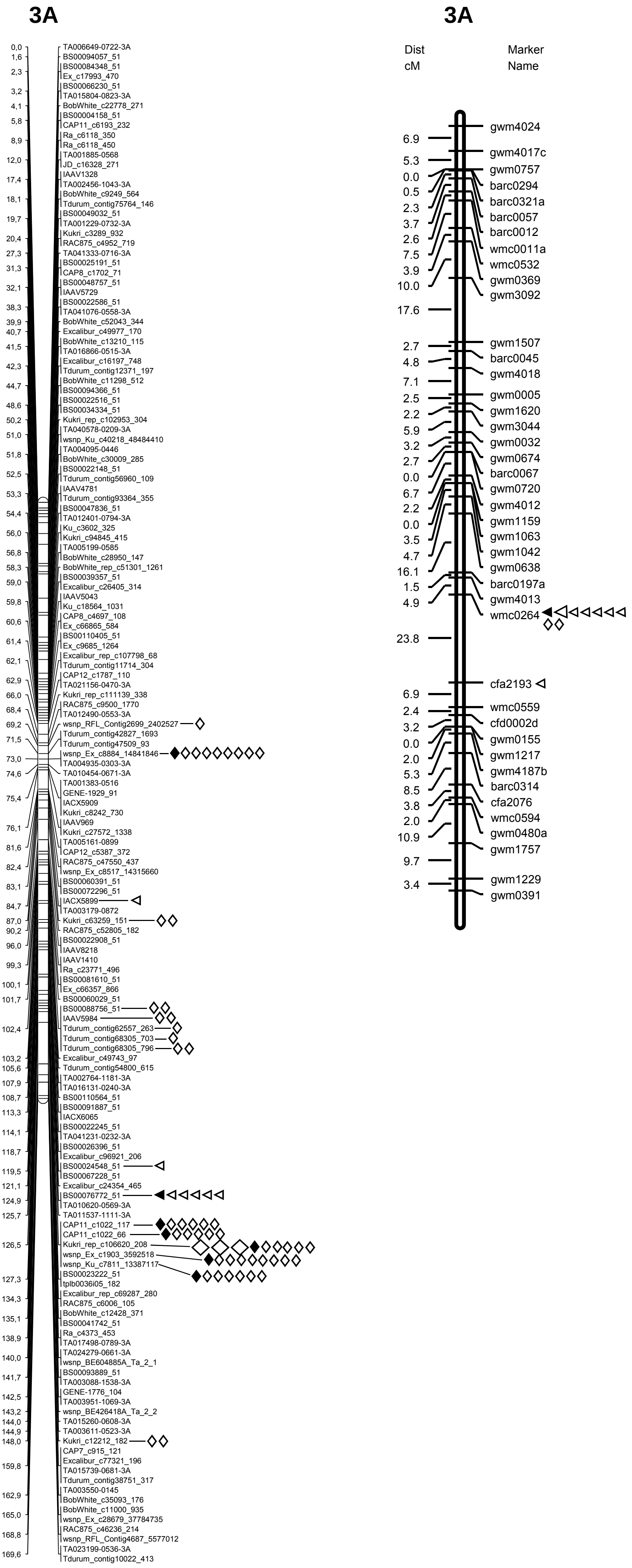
3A
0,0
1,6
2,3
3,2
4,1
5,8
8,9
12,0
17,4
18,1
19,7
20,4
27,3
31,3
32,1
38,3
39,9
40,7
41,5
42,3
44,7
48,6
50,2
51,0
51,8
52,5
53,3
54,4
56,0
56,8
58,3
59,0
59,8
60,6
61,4
62,1
62,9
66,0
68,4
69,2
71,5
73,0
74,6
75,4
76,1
81,6
82,4
83,1
84,7
87,0
90,2
96,0
99,3
100,1
101,7
102,4
103,2
105,6
107,9
108,7
113,3
114,1
118,7
119,5
121,1
124,9
125,7
126,5
127,3
134,3
135,1
138,9
140,0
141,7
142,5
143,2
144,0
144,9
148,0
159,8
162,9
165,0
168,8
169,6
TA006649-0722-3A
BS00094057_51
BS00084348_51
Ex_c17993_470
BS00066230_51
TA015804-0823-3A
BobWhite_c22778_271
BS00004158_51
CAP11_c6193_232
Ra_c6118_350
Ra_c6118_450
TA001885-0568
JD_c16328_271
IAAV1328
TA002456-1043-3A
BobWhite_c9249_564
Tdurum_contig75764_146
BS00049032_51
TA001229-0732-3A
Kukri_c3289_932
RAC875_c4952_719
TA041333-0716-3A
BS00025191_51
CAP8_c1702_71
BS00048757_51
IAAV5729
BS00022586_51
TA041076-0558-3A
BobWhite_c52043_344
Excalibur_c49977_170
BobWhite_c13210_115
TA016866-0515-3A
Excalibur_c16197_748
Tdurum_contig12371_197
BobWhite_c11298_512
BS00094366_51
BS00022516_51
BS00034334_51
Kukri_rep_c102953_304
TA040578-0209-3A
wsnp_Ku_c40218_48484410
TA004095-0446
BobWhite_c30009_285
BS00022148_51
Tdurum_contig56960_109
IAAV4781
Tdurum_contig93364_355
BS00047836_51
TA012401-0794-3A
Ku_c3602_325
Kukri_c94845_415
TA005199-0585
BobWhite_c28950_147
BobWhite_rep_c51301_1261
BS00039357_51
Excalibur_c26405_314
IAAV5043
Ku_c18564_1031
CAP8_c4697_108
Ex_c66865_584
BS00110405_51
Ex_c9685_1264
Excalibur_rep_c107798_68
Tdurum_contig11714_304
CAP12_c1787_110
TA021156-0470-3A
Kukri_rep_c111139_338
RAC875_c9500_1770
TA012490-0553-3A
wsnp_RFL_Contig2699_2402527
Tdurum_contig42827_1693
Tdurum_contig47509_93
wsnp_Ex_c8884_14841846
TA004935-0303-3A
TA010454-0671-3A
TA001383-0516
GENE-1929_91
IACX5909
Kukri_c8242_730
IAAV969
Kukri_c27572_1338
TA005161-0899
CAP12_c5387_372
RAC875_c47550_437
wsnp_Ex_c8517_14315660
BS00060391_51
BS00072296_51
IACX5899
TA003179-0872
Kukri_c63259_151
RAC875_c52805_182
BS00022908_51
IAAV8218
IAAV1410
Ra_c23771_496
BS00081610_51
Ex_c66357_866
BS00060029_51
BS00088756_51
IAAV5984
Tdurum_contig62557_263
Tdurum_contig68305_703
Tdurum_contig68305_796
Excalibur_c49743_97
Tdurum_contig54800_615
TA002764-1181-3A
TA016131-0240-3A
BS00110564_51
BS00091887_51
IACX6065
BS00022245_51
TA041231-0232-3A
BS00026396_51
Excalibur_c96921_206
BS00024548_51
BS00067228_51
Excalibur_c24354_465
BS00076772_51
TA010620-0569-3A
TA011537-1111-3A
CAP11_c1022_117
CAP11_c1022_66
Kukri_rep_c106620_208
wsnp_Ex_c1903_3592518
wsnp_Ku_c7811_13387117
BS00023222_51
tplb0036i05_182
Excalibur_rep_c69287_280
RAC875_c6006_105
BobWhite_c12428_371
BS00041742_51
Ra_c4373_453
TA017498-0789-3A
TA024279-0661-3A
wsnp_BE604885A_Ta_2_1
BS00093889_51
TA003088-1538-3A
GENE-1776_104
TA003951-1069-3A
wsnp_BE426418A_Ta_2_2
TA015260-0608-3A
TA003611-0523-3A
Kukri_c12212_182
CAP7_c915_121
Excalibur_c77321_196
TA015739-0681-3A
Tdurum_contig38751_317
TA003550-0145
BobWhite_c35093_176
BobWhite_c11000_935
wsnp_Ex_c28679_37784735
RAC875_c46236_214
wsnp_RFL_Contig4687_5577012
TA023199-0536-3A
Tdurum_contig10022_413
3A
Dist
cM
Marker
Name
gwm4024
6.9
gwm4017c
5.3
0.0
gwm0757
0.5
barc0294
2.3
barc0321a
3.7
barc0057
2.6
barc0012
7.5
wmc0011a
3.9
wmc0532
10.0
gwm0369
gwm3092
17.6
2.7
gwm1507
4.8
barc0045
7.1
gwm4018
2.5
gwm0005
2.2
gwm1620
5.9
gwm3044
3.2
gwm0032
2.7
gwm0674
0.0
barc0067
6.7
gwm0720
2.2
gwm4012
0.0
gwm1159
3.5
gwm1063
4.7
gwm1042
16.1
gwm0638
1.5
barc0197a
4.9
gwm4013
wmc0264
23.8
cfa2193
6.9
2.4
wmc0559
3.2
cfd0002d
0.0
gwm0155
2.0
gwm1217
5.3
gwm4187b
8.5
barc0314
3.8
cfa2076
2.0
wmc0594
10.9
gwm0480a
gwm1757
9.7
3.4
gwm1229
gwm0391

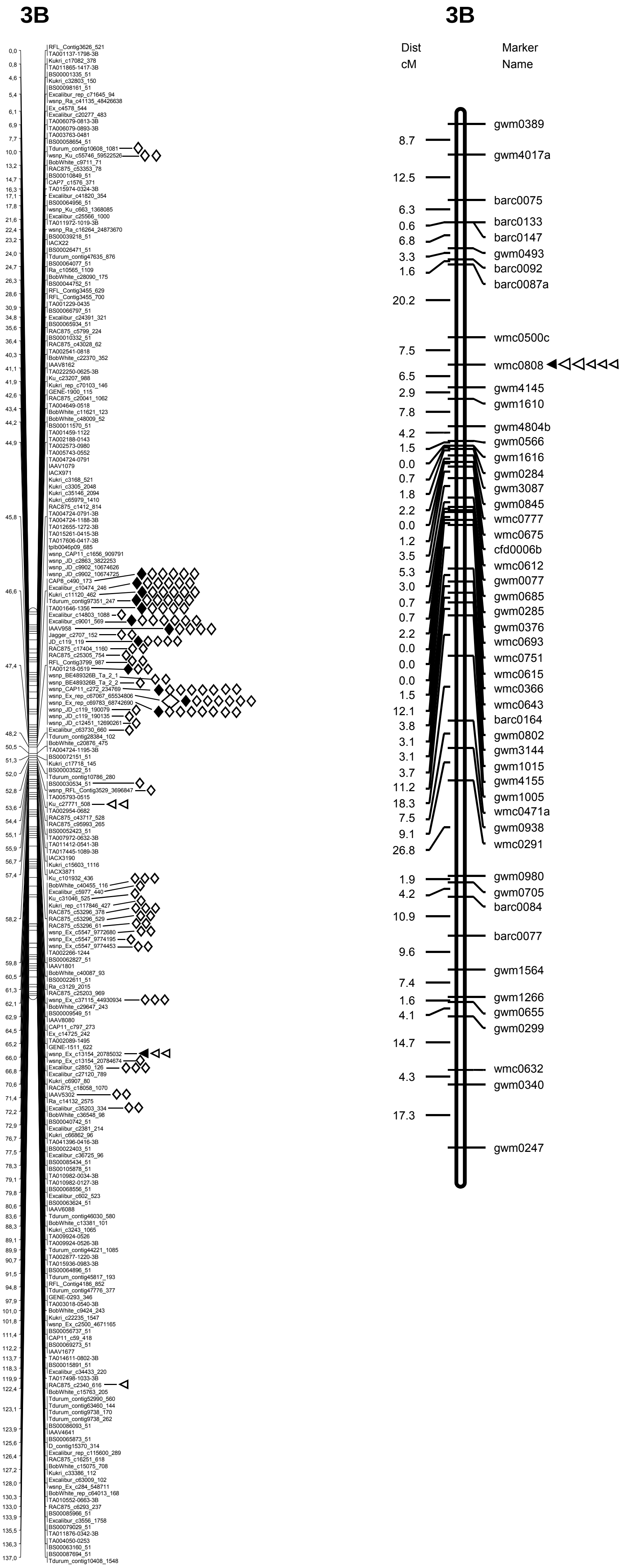
3B
3B
Dist cM
Marker Name
gwm0389
gwm4017a
barc0075
barc0133
barc0147
gwm0493
barc0092
barc0087a
wmc0500c
wmc0808
gwm4145
gwm1610
gwm4804b
gwm0566
gwm1616
gwm0284
gwm3087
gwm0845
wmc0777
wmc0675
cfd0006b
wmc0612
gwm0077
gwm0685
gwm0285
gwm0376
wmc0693
wmc0751
wmc0615
wmc0366
wmc0643
barc0164
gwm0802
gwm3144
gwm1015
gwm4155
gwm1005
wmc0471a
gwm0938
wmc0291
gwm0980
gwm0705
barc0084
barc0077
gwm1564
gwm1266
gwm0655
gwm0299
wmc0632
gwm0340
gwm0247

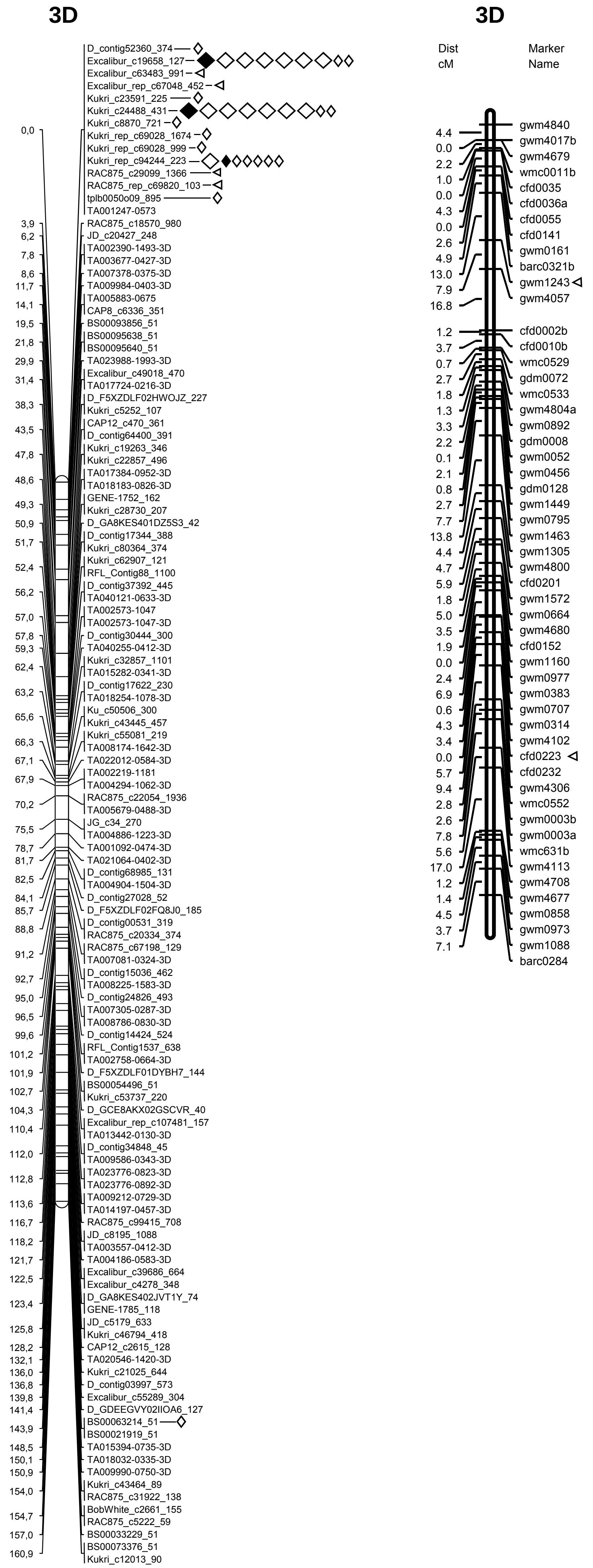
3D
D_contig52360_374
Excalibur_c19658_127
Excalibur_c63483_991
Excalibur_rep_c67048_452
Kukri_c23591_225
Kukri_c24488_431
Kukri_c8870_721
Kukri_rep_c69028_1674
Kukri_rep_c69028_999
Kukri_rep_c94244_223
RAC875_c29099_1366
RAC875_rep_c69820_103
tplb0050o09_895
TA001247-0573
RAC875_c18570_980
JD_c20427_248
TA002390-1493-3D
TA003677-0427-3D
TA007378-0375-3D
TA009984-0403-3D
TA005883-0675
CAP8_c6336_351
BS00093856_51
BS00095638_51
BS00095640_51
TA023988-1993-3D
Excalibur_c49018_470
TA017724-0216-3D
D_F5XZDLF02HWOJZ_227
Kukri_c5252_107
CAP12_c470_361
D_contig64400_391
Kukri_c19263_346
Kukri_c22857_496
TA017384-0952-3D
TA018183-0826-3D
GENE-1752_162
Kukri_c28730_207
D_GA8KES401DZ5S3_42
D_contig17344_388
Kukri_c80364_374
Kukri_c62907_121
RFL_Contig88_1100
D_contig37392_445
TA040121-0633-3D
TA002573-1047
TA002573-1047-3D
D_contig30444_300
TA040255-0412-3D
Kukri_c32857_1101
TA015282-0341-3D
D_contig17622_230
TA018254-1078-3D
Ku_c50506_300
Kukri_c43445_457
Kukri_c55081_219
TA008174-1642-3D
TA022012-0584-3D
TA002219-1181
TA004294-1062-3D
RAC875_c22054_1936
TA005679-0488-3D
JG_c34_270
TA004886-1223-3D
TA001092-0474-3D
TA021064-0402-3D
D_contig68985_131
TA004904-1504-3D
D_contig27028_52
D_F5XZDLF02FQ8J0_185
D_contig00531_319
RAC875_c20334_374
RAC875_c67198_129
TA007081-0324-3D
D_contig15036_462
TA008225-1583-3D
D_contig24826_493
TA007305-0287-3D
TA008786-0830-3D
D_contig14424_524
RFL_Contig1537_638
TA002758-0664-3D
D_F5XZDLF01DYBH7_144
BS00054496_51
Kukri_c53737_220
D_GCE8AKX02GSCVR_40
Excalibur_rep_c107481_157
TA013442-0130-3D
D_contig34848_45
TA009586-0343-3D
TA023776-0823-3D
TA023776-0892-3D
TA009212-0729-3D
TA014197-0457-3D
RAC875_c99415_708
JD_c8195_1088
TA003557-0412-3D
TA004186-0583-3D
Excalibur_c39686_664
Excalibur_c4278_348
D_GA8KES402JVT1Y_74
GENE-1785_118
JD_c5179_633
Kukri_c46794_418
CAP12_c2615_128
TA020546-1420-3D
Kukri_c21025_644
D_contig03997_573
Excalibur_c55289_304
D_GDEEGVY02IIOA6_127
BS00063214_51
BS00021919_51
TA015394-0735-3D
TA018032-0335-3D
TA009990-0750-3D
Kukri_c43464_89
RAC875_c31922_138
BobWhite_c2661_155
RAC875_c5222_59
BS00033229_51
BS00073376_51
Kukri_c12013_90
0,0
3,9
6,2
7,8
8,6
11,7
14,1
19,5
21,8
29,9
31,4
38,3
43,5
47,8
48,6
49,3
50,9
51,7
52,4
56,2
57,0
57,8
59,3
62,4
63,2
65,6
66,3
67,1
67,9
70,2
75,5
78,7
81,7
82,5
84,1
85,7
88,8
91,2
92,7
95,0
96,5
99,6
101,2
101,9
102,7
104,3
110,4
112,0
112,8
113,6
116,7
118,2
121,7
122,5
123,4
125,8
128,2
132,1
136,0
136,8
139,8
141,4
143,9
148,5
150,1
150,9
154,0
154,7
157,0
160,9
3D
Dist cM
Marker Name
4.4 gwm4840
0.0 gwm4017b
2.2 gwm4679
1.0 wmc0011b
0.0 cfd0035
4.3 cfd0036a
0.0 cfd0055
2.6 cfd0141
4.9 gwm0161
13.0 barc0321b
7.9 gwm1243
16.8 gwm4057
1.2 cfd0002b
3.7 cfd0010b
0.7 wmc0529
2.7 gdm0072
1.8 wmc0533
1.3 gwm4804a
3.3 gwm0892
2.2 gdm0008
0.1 gwm0052
2.1 gwm0456
0.8 gdm0128
2.7 gwm1449
7.7 gwm0795
13.8 gwm1463
4.4 gwm1305
4.7 gwm4800
5.9 cfd0201
1.8 gwm1572
5.0 gwm0664
3.5 gwm4680
1.9 cfd0152
0.0 gwm1160
2.4 gwm0977
6.9 gwm0383
0.6 gwm0707
4.3 gwm0314
0.0 gwm4102
5.7 cfd0223
9.4 cfd0232
2.8 gwm4306
2.6 wmc0552
7.8 gwm0003b
5.6 gwm0003a
17.0 wmc631b
1.2 gwm4113
1.4 gwm4708
4.5 gwm4677
3.7 gwm0858
7.1 gwm0973
gwm1088
barc0284

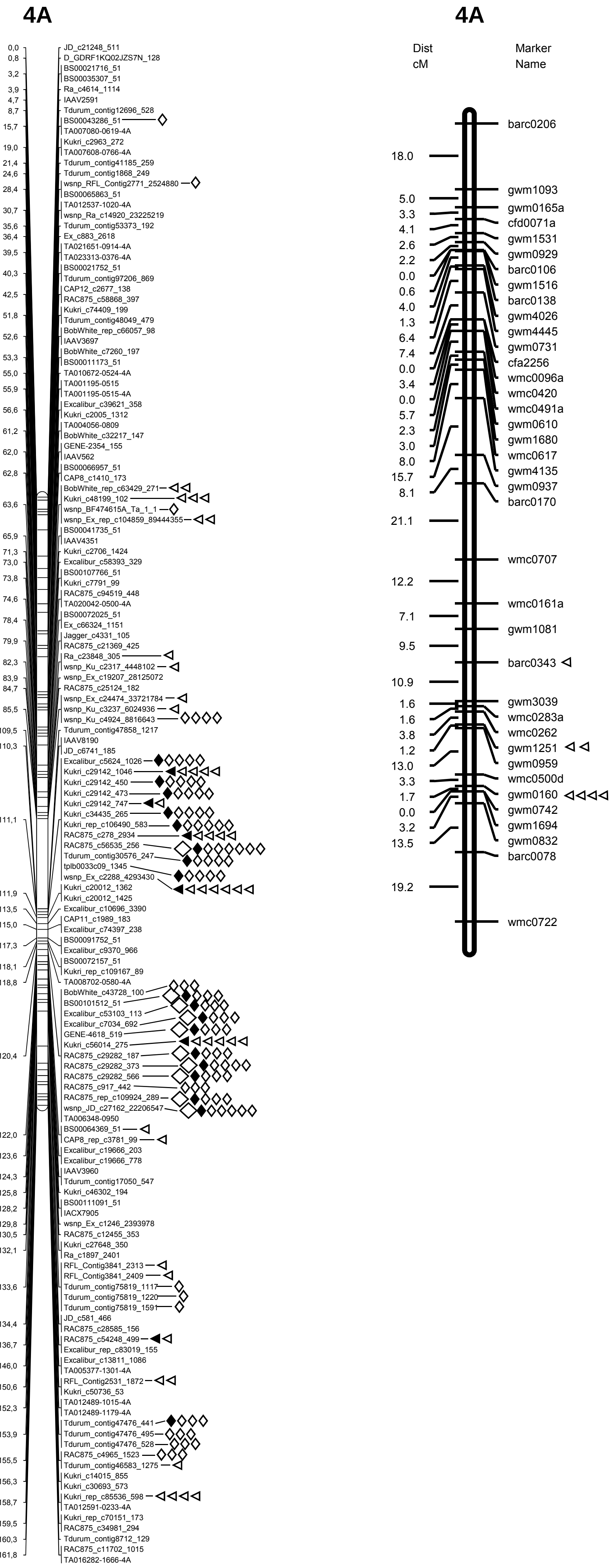
4A
0,0
0,8
3,2
3,9
4,7
8,7
15,7
19,0
21,4
24,6
28,4
30,7
35,6
36,4
39,5
40,3
42,5
51,8
52,6
53,3
55,0
55,9
56,6
61,2
62,0
62,8
63,6
65,9
71,3
73,0
73,8
74,6
78,4
79,9
82,3
83,9
84,7
85,5
109,5
110,3
111,1
111,9
113,5
115,0
117,3
118,1
118,8
120,4
122,0
123,6
124,3
125,8
128,2
129,8
130,5
132,1
133,6
134,4
136,7
146,0
150,6
152,3
153,9
155,5
156,3
158,7
159,5
160,3
161,8
JD_c21248_511
D_GDRF1KQ02JZS7N_128
BS00021716_51
BS00035307_51
Ra_c4614_1114
IAAV2591
Tdurum_contig12696_528
BS00043286_51
TA007080-0619-4A
Kukri_c2963_272
TA007608-0766-4A
Tdurum_contig41185_259
Tdurum_contig1868_249
wsnp_RFL_Contig2771_2524880
BS00065863_51
TA012537-1020-4A
wsnp_Ra_c14920_23225219
Tdurum_contig53373_192
Ex_c883_2618
TA021651-0914-4A
TA023313-0376-4A
BS00021752_51
Tdurum_contig97206_869
CAP12_c2677_138
RAC875_c58868_397
Kukri_c74409_199
Tdurum_contig48049_479
BobWhite_rep_c66057_98
IAAV3697
BobWhite_c7260_197
BS00011173_51
TA010672-0524-4A
TA001195-0515
TA001195-0515-4A
Excalibur_c39621_358
Kukri_c2005_1312
TA004056-0809
BobWhite_c32217_147
GENE-2354_155
IAAV562
BS00066957_51
CAP8_c1410_173
BobWhite_rep_c63429_271
Kukri_c48199_102
wsnp_BF474615A_Ta_1_1
wsnp_Ex_rep_c104859_89444355
BS00041735_51
IAAV4351
Kukri_c2706_1424
Excalibur_c58393_329
BS00107766_51
Kukri_c7791_99
RAC875_c94519_448
TA020042-0500-4A
BS00072025_51
Ex_c66324_1151
Jagger_c4331_105
RAC875_c21369_425
Ra_c23848_305
wsnp_Ku_c2317_4448102
wsnp_Ex_c19207_28125072
RAC875_c25124_182
wsnp_Ex_c24474_33721784
wsnp_Ku_c3237_6024936
wsnp_Ku_c4924_8816643
Tdurum_contig47858_1217
IAAV8190
JD_c6741_185
Excalibur_c5624_1026
Kukri_c29142_1046
Kukri_c29142_450
Kukri_c29142_473
Kukri_c29142_747
Kukri_c34435_265
Kukri_rep_c106490_583
RAC875_c278_2934
RAC875_c56535_256
Tdurum_contig30576_247
tplb0033c09_1345
wsnp_Ex_c2288_4293430
Kukri_c20012_1362
Kukri_c20012_1425
Excalibur_c10696_3390
CAP11_c1989_183
Excalibur_c74397_238
BS00091752_51
Excalibur_c9370_966
BS00072157_51
Kukri_rep_c109167_89
TA008702-0580-4A
BobWhite_c43728_100
BS00101512_51
Excalibur_c53103_113
Excalibur_c7034_692
GENE-4618_519
Kukri_c56014_275
RAC875_c29282_187
RAC875_c29282_373
RAC875_c29282_566
RAC875_c917_442
RAC875_rep_c109924_289
wsnp_JD_c27162_22206547
TA006348-0950
BS00064369_51
CAP8_rep_c3781_99
Excalibur_c19666_203
Excalibur_c19666_778
IAAV3960
Tdurum_contig17050_547
Kukri_c46302_194
BS00111091_51
IACX7905
wsnp_Ex_c1246_2393978
RAC875_c12455_353
Kukri_c27648_350
Ra_c1897_2401
RFL_Contig3841_2313
RFL_Contig3841_2409
Tdurum_contig75819_1117
Tdurum_contig75819_1220
Tdurum_contig75819_1591
JD_c581_466
RAC875_c28585_156
RAC875_c54248_499
Excalibur_rep_c83019_155
Excalibur_c13811_1086
TA005377-1301-4A
RFL_Contig2531_1872
Kukri_c50736_53
TA012489-1015-4A
TA012489-1179-4A
Tdurum_contig47476_441
Tdurum_contig47476_495
Tdurum_contig47476_528
RAC875_c4965_1523
Tdurum_contig46583_1275
Kukri_c14015_855
Kukri_c30693_573
Kukri_rep_c85536_598
TA012591-0233-4A
Kukri_rep_c70151_173
RAC875_c34981_294
Tdurum_contig8712_129
RAC875_c11702_1015
TA016282-1666-4A
4A
Dist
cM
Marker
Name
barc0206
18.0
gwm1093
5.0
gwm0165a
3.3
cfd0071a
4.1
gwm1531
2.6
gwm0929
2.2
barc0106
0.0
gwm1516
0.6
barc0138
4.0
gwm4026
1.3
gwm4445
6.4
gwm0731
7.4
cfa2256
0.0
wmc0096a
3.4
wmc0420
0.0
wmc0491a
5.7
gwm0610
2.3
gwm1680
3.0
wmc0617
8.0
gwm4135
15.7
gwm0937
8.1
barc0170
21.1
wmc0707
12.2
wmc0161a
7.1
gwm1081
9.5
barc0343
10.9
gwm3039
1.6
wmc0283a
1.6
wmc0262
3.8
gwm1251
1.2
gwm0959
13.0
wmc0500d
3.3
gwm0160
1.7
gwm0742
0.0
gwm1694
3.2
gwm0832
13.5
barc0078
19.2
wmc0722

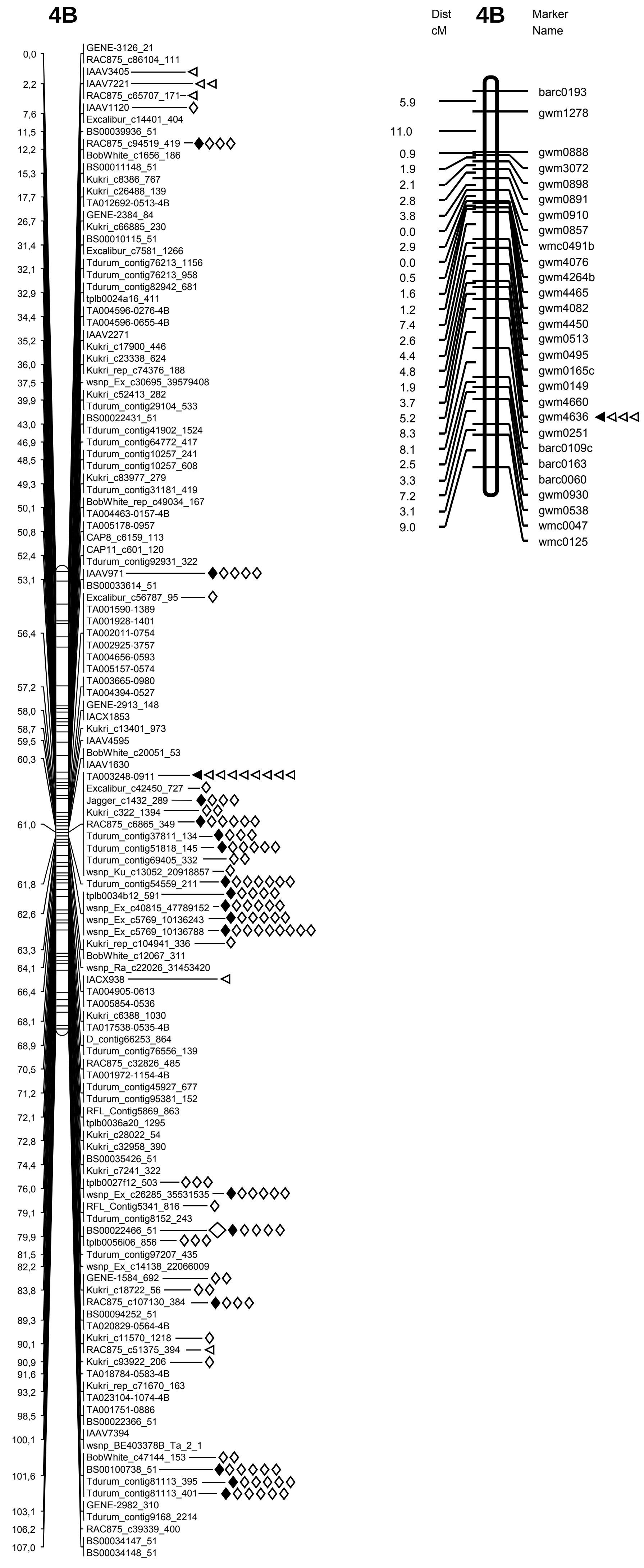
4B
0,0
2,2
7,6
11,5
12,2
15,3
17,7
26,7
31,4
32,1
32,9
34,4
35,2
36,0
37,5
39,9
43,0
46,9
48,5
49,3
50,1
50,8
52,4
53,1
56,4
57,2
58,0
58,7
59,5
60,3
61,0
61,8
62,6
63,3
64,1
66,4
68,1
68,9
70,5
71,2
72,1
72,8
74,4
76,0
79,1
79,9
81,5
82,2
83,8
89,3
90,1
90,9
91,6
93,2
98,5
100,1
101,6
103,1
106,2
107,0
GENE-3126_21
RAC875_c86104_111
IAAV3405
IAAV7221
RAC875_c65707_171
IAAV1120
Excalibur_c14401_404
BS00039936_51
RAC875_c94519_419
BobWhite_c1656_186
BS00011148_51
Kukri_c8386_767
Kukri_c26488_139
TA012692-0513-4B
GENE-2384_84
Kukri_c66885_230
BS00010115_51
Excalibur_c7581_1266
Tdurum_contig76213_1156
Tdurum_contig76213_958
Tdurum_contig82942_681
tplb0024a16_411
TA004596-0276-4B
TA004596-0655-4B
IAAV2271
Kukri_c17900_446
Kukri_c23338_624
Kukri_rep_c74376_188
wsnp_Ex_c30695_39579408
Kukri_c52413_282
Tdurum_contig29104_533
BS00022431_51
Tdurum_contig41902_1524
Tdurum_contig64772_417
Tdurum_contig10257_241
Tdurum_contig10257_608
Kukri_c83977_279
Tdurum_contig31181_419
BobWhite_rep_c49034_167
TA004463-0157-4B
TA005178-0957
CAP8_c6159_113
CAP11_c601_120
Tdurum_contig92931_322
IAAV971
BS00033614_51
Excalibur_c56787_95
TA001590-1389
TA001928-1401
TA002011-0754
TA002925-3757
TA004656-0593
TA005157-0574
TA003665-0980
TA004394-0527
GENE-2913_148
IACX1853
Kukri_c13401_973
IAAV4595
BobWhite_c20051_53
IAAV1630
TA003248-0911
Excalibur_c42450_727
Jagger_c1432_289
Kukri_c322_1394
RAC875_c6865_349
Tdurum_contig37811_134
Tdurum_contig51818_145
Tdurum_contig69405_332
wsnp_Ku_c13052_20918857
Tdurum_contig54559_211
tplb0034b12_591
wsnp_Ex_c40815_47789152
wsnp_Ex_c5769_10136243
wsnp_Ex_c5769_10136788
Kukri_rep_c104941_336
BobWhite_c12067_311
wsnp_Ra_c22026_31453420
IACX938
TA004905-0613
TA005854-0536
Kukri_c6388_1030
TA017538-0535-4B
D_contig66253_864
Tdurum_contig76556_139
RAC875_c32826_485
TA001972-1154-4B
Tdurum_contig45927_677
Tdurum_contig95381_152
RFL_Contig5869_863
tplb0036a20_1295
Kukri_c28022_54
Kukri_c32958_390
BS00035426_51
Kukri_c7241_322
tplb0027f12_503
wsnp_Ex_c26285_35531535
RFL_Contig5341_816
Tdurum_contig8152_243
BS00022466_51
tplb0056i06_856
Tdurum_contig97207_435
wsnp_Ex_c14138_22066009
GENE-1584_692
Kukri_c18722_56
RAC875_c107130_384
BS00094252_51
TA020829-0564-4B
Kukri_c11570_1218
RAC875_c51375_394
Kukri_c93922_206
TA018784-0583-4B
Kukri_rep_c71670_163
TA023104-1074-4B
TA001751-0886
BS00022366_51
IAAV7394
wsnp_BE403378B_Ta_2_1
BobWhite_c47144_153
BS00100738_51
Tdurum_contig81113_395
Tdurum_contig81113_401
GENE-2982_310
Tdurum_contig9168_2214
RAC875_c39339_400
BS00034147_51
BS00034148_51
Dist cM
4B
Marker Name
5.9
11.0
0.9
1.9
2.1
2.8
3.8
0.0
2.9
0.0
0.5
1.6
1.2
7.4
2.6
4.4
4.8
1.9
3.7
5.2
8.3
8.1
2.5
3.3
7.2
3.1
9.0
barc0193
gwm1278
gwm0888
gwm3072
gwm0898
gwm0891
gwm0910
gwm0857
wmc0491b
gwm4076
gwm4264b
gwm4465
gwm4082
gwm4450
gwm0513
gwm0495
gwm0165c
gwm0149
gwm4660
gwm4636
gwm0251
barc0109c
barc0163
barc0060
gwm0930
gwm0538
wmc0047
wmc0125

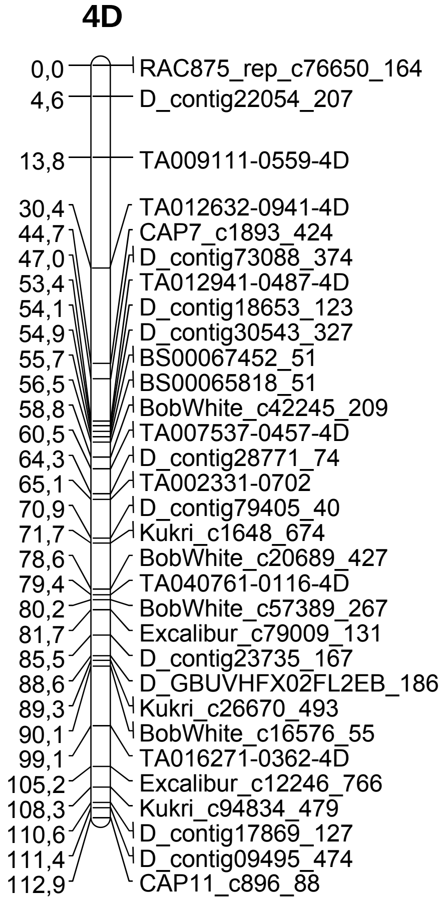
4D
0,0 RAC875_rep_c76650_164
4,6 D_contig22054_207
13,8 TA009111-0559-4D
30,4 TA012632-0941-4D
44,7 CAP7_c1893_424
47,0 D_contig73088_374
53,4 TA012941-0487-4D
54,1 D_contig18653_123
54,9 D_contig30543_327
55,7 BS00067452_51
56,5 BS00065818_51
58,8 BobWhite_c42245_209
60,5 TA007537-0457-4D
64,3 D_contig28771_74
65,1 TA002331-0702
70,9 D_contig79405_40
71,7 Kukri_c1648_674
78,6 BobWhite_c20689_427
79,4 TA040761-0116-4D
80,2 BobWhite_c57389_267
81,7 Excalibur_c79009_131
85,5 D_contig23735_167
88,6 D_GBUVHFX02FL2EB_186
89,3 Kukri_c26670_493
90,1 BobWhite_c16576_55
99,1 TA016271-0362-4D
105,2 Excalibur_c12246_766
108,3 Kukri_c94834_479
110,6 D_contig17869_127
111,4 D_contig09495_474
112,9 CAP11_c896_88

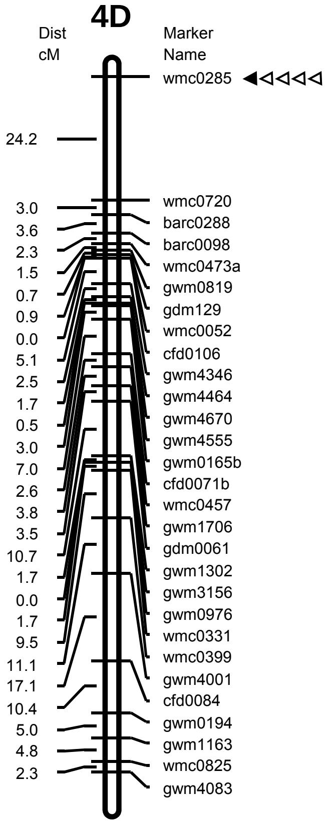
4D
Dist cM
Marker Name
wmc0285
24.2
3.0 wmc0720
3.6 barc0288
2.3 barc0098
1.5 wmc0473a
0.7 gwm0819
0.9 gdm129
0.0 wmc0052
5.1 cfd0106
2.5 gwm4346
1.7 gwm4464
0.5 gwm4670
3.0 gwm4555
7.0 gwm0165b
2.6 cfd0071b
3.8 wmc0457
3.5 gwm1706
10.7 gdm0061
1.7 gwm1302
0.0 gwm3156
1.7 gwm0976
9.5 wmc0331
11.1 wmc0399
17.1 gwm4001
10.4 cfd0084
5.0 gwm0194
4.8 gwm1163
2.3 wmc0825
gwm4083

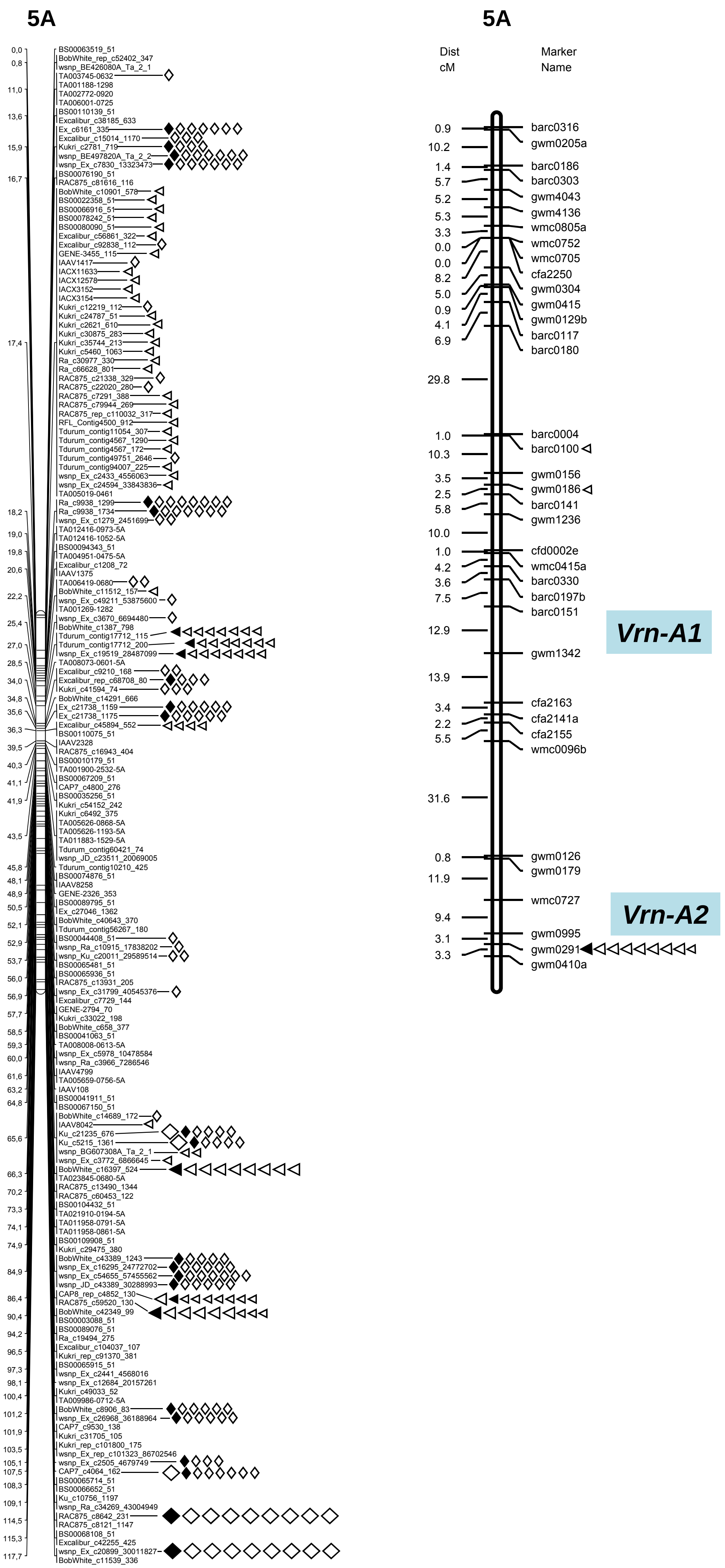
5A
0,0
0,8
11,0
13,6
15,9
16,7
17,4
18,2
19,0
19,8
20,6
22,2
25,4
27,0
28,5
34,0
34,8
35,6
36,3
39,5
40,3
41,1
41,9
43,5
45,8
48,1
48,9
50,5
52,1
52,9
53,7
56,0
56,9
57,7
58,5
59,3
60,0
61,6
63,2
64,8
65,6
66,3
70,2
73,3
74,1
74,9
84,9
86,4
90,4
94,2
96,5
97,3
98,1
100,4
101,2
101,9
103,5
105,1
107,5
108,3
109,1
114,5
115,3
117,7
BS00063519_51
BobWhite_rep_c52402_347
wsnp_BE426080A_Ta_2_1
TA003745-0632
TA001188-1298
TA002772-0920
TA006001-0725
BS00110139_51
Excalibur_c38185_633
Ex_c6161_335
Excalibur_c15014_1170
Kukri_c2781_719
wsnp_BE497820A_Ta_2_2
wsnp_Ex_c7830_13323473
BS00076190_51
RAC875_c81616_116
BobWhite_c10901_578
BS00022358_51
BS00066916_51
BS00078242_51
BS00080090_51
Excalibur_c56861_322
Excalibur_c92838_112
GENE-3455_115
IAAV1417
IACX11633
IACX12578
IACX3152
IACX3154
Kukri_c12219_112
Kukri_c24787_51
Kukri_c2621_610
Kukri_c30875_283
Kukri_c35744_213
Kukri_c5460_1063
Ra_c30977_330
Ra_c66628_801
RAC875_c21338_329
RAC875_c22020_280
RAC875_c7291_388
RAC875_c79944_269
RAC875_rep_c110032_317
RFL_Contig4500_912
Tdurum_contig11054_307
Tdurum_contig4567_1290
Tdurum_contig4567_172
Tdurum_contig49751_2646
Tdurum_contig94007_225
wsnp_Ex_c2433_4556063
wsnp_Ex_c24594_33843836
TA005019-0461
Ra_c9938_1299
Ra_c9938_1734
wsnp_Ex_c1279_2451699
TA012416-0973-5A
TA012416-1052-5A
BS00094343_51
TA004951-0475-5A
Excalibur_c1208_72
IAAV1375
TA006419-0680
BobWhite_c11512_157
wsnp_Ex_c49211_53875600
TA001269-1282
wsnp_Ex_c3670_6694480
BobWhite_c1387_798
Tdurum_contig17712_115
Tdurum_contig17712_200
wsnp_Ex_c19519_28487099
TA008073-0601-5A
Excalibur_c9210_168
Excalibur_rep_c68708_80
Kukri_c41594_74
BobWhite_c14291_666
Ex_c21738_1159
Ex_c21738_1175
Excalibur_c45894_552
BS00110075_51
IAAV2328
RAC875_c16943_404
BS00010179_51
TA001900-2532-5A
BS00067209_51
CAP7_c4800_276
BS00035256_51
Kukri_c54152_242
Kukri_c6492_375
TA005626-0868-5A
TA005626-1193-5A
TA011883-1529-5A
Tdurum_contig60421_74
wsnp_JD_c23511_20069005
Tdurum_contig10210_425
BS00074876_51
IAAV8258
GENE-2326_353
BS00089795_51
Ex_c27046_1362
BobWhite_c40643_370
Tdurum_contig56267_180
BS00044408_51
wsnp_Ra_c10915_17838202
wsnp_Ku_c20011_29589514
BS00065481_51
BS00065936_51
RAC875_c13931_205
wsnp_Ex_c31799_40545376
Excalibur_c7729_144
GENE-2794_70
Kukri_c33022_198
BobWhite_c658_377
BS00041063_51
TA008008-0613-5A
wsnp_Ex_c5978_10478584
wsnp_Ra_c3966_7286546
IAAV4799
TA005659-0756-5A
IAAV108
BS00041911_51
BS00067150_51
BobWhite_c14689_172
IAAV8042
Ku_c21235_676
Ku_c5215_1361
wsnp_BG607308A_Ta_2_1
wsnp_Ex_c3772_6866645
BobWhite_c16397_524
TA023845-0680-5A
RAC875_c13490_1344
RAC875_c60453_122
BS00104432_51
TA021910-0194-5A
TA011958-0791-5A
TA011958-0861-5A
BS00109908_51
Kukri_c29475_380
BobWhite_c43389_1243
wsnp_Ex_c16295_24772702
wsnp_Ex_c54655_57455562
wsnp_JD_c43389_30288993
CAP8_rep_c4852_130
RAC875_c59520_130
BobWhite_c42349_99
BS00003088_51
BS00089076_51
Ra_c19494_275
Excalibur_c104037_107
Kukri_rep_c91370_381
BS00065915_51
wsnp_Ex_c2441_4568016
wsnp_Ex_c12684_20157261
Kukri_c49033_52
TA009986-0712-5A
BobWhite_c8906_83
wsnp_Ex_c26968_36188964
CAP7_c9530_138
Kukri_c31705_105
Kukri_rep_c101800_175
wsnp_Ex_rep_c101323_86702546
wsnp_Ex_c2505_4679749
CAP7_c4064_162
BS00065714_51
BS00066652_51
Ku_c10756_1197
wsnp_Ra_c34269_43004949
RAC875_c8642_231
RAC875_c8121_1147
BS00068108_51
Excalibur_c42255_425
wsnp_Ex_c20899_30011827
BobWhite_c11539_336
5A
Dist cM
Marker Name
0.9
10.2
1.4
5.7
5.2
5.3
3.3
0.0
0.0
8.2
5.0
0.9
4.1
6.9
29.8
1.0
10.3
3.5
2.5
5.8
10.0
1.0
4.2
3.6
7.5
12.9
13.9
3.4
2.2
5.5
31.6
0.8
11.9
9.4
3.1
3.3
barc0316
gwm0205a
barc0186
barc0303
gwm4043
gwm4136
wmc0805a
wmc0752
wmc0705
cfa2250
gwm0304
gwm0415
gwm0129b
barc0117
barc0180
barc0004
barc0100
gwm0156
gwm0186
barc0141
gwm1236
cfd0002e
wmc0415a
barc0330
barc0197b
barc0151
Vrn-A1
gwm1342
cfa2163
cfa2141a
cfa2155
wmc0096b
gwm0126
gwm0179
wmc0727
Vrn-A2
gwm0995
gwm0291
gwm0410a

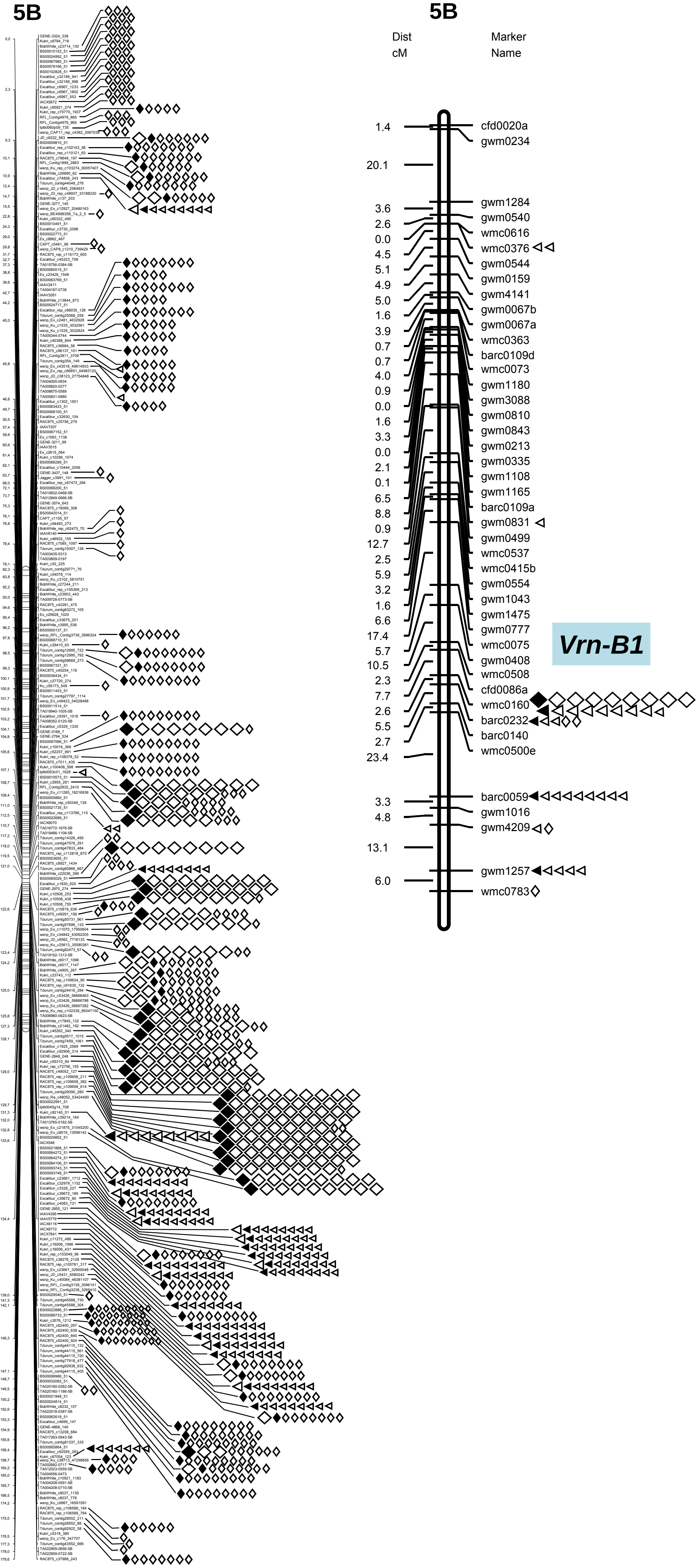

5B
5B
Dist cM
Marker Name
1.4 cfd0020a
gwm0234
20.1
gwm1284
3.6 gwm0540
2.6 wmc0616
0.0 wmc0376
4.5 gwm0544
5.1 gwm0159
4.9 gwm4141
5.0 gwm0067b
1.6 gwm0067a
3.9 wmc0363
0.7 barc0109d
0.7 wmc0073
4.0 gwm1180
0.9 gwm3088
0.0 gwm0810
1.6 gwm0843
3.3 gwm0213
0.0 gwm0335
2.1 gwm1108
0.1 gwm1165
6.5 barc0109a
8.8 gwm0831
0.9 gwm0499
12.7 wmc0537
2.5 wmc0415b
5.9 gwm0554
3.2 gwm1043
1.6 gwm1475
6.6 gwm0777
17.4 wmc0075
5.7 gwm0408
10.5 wmc0508
2.3 cfd0086a
7.7 wmc0160
2.6 barc0232
5.5 barc0140
2.7 wmc0500e
23.4
barc0059
3.3 gwm1016
4.8 gwm4209
13.1
gwm1257
6.0 wmc0783
Vrn-B1

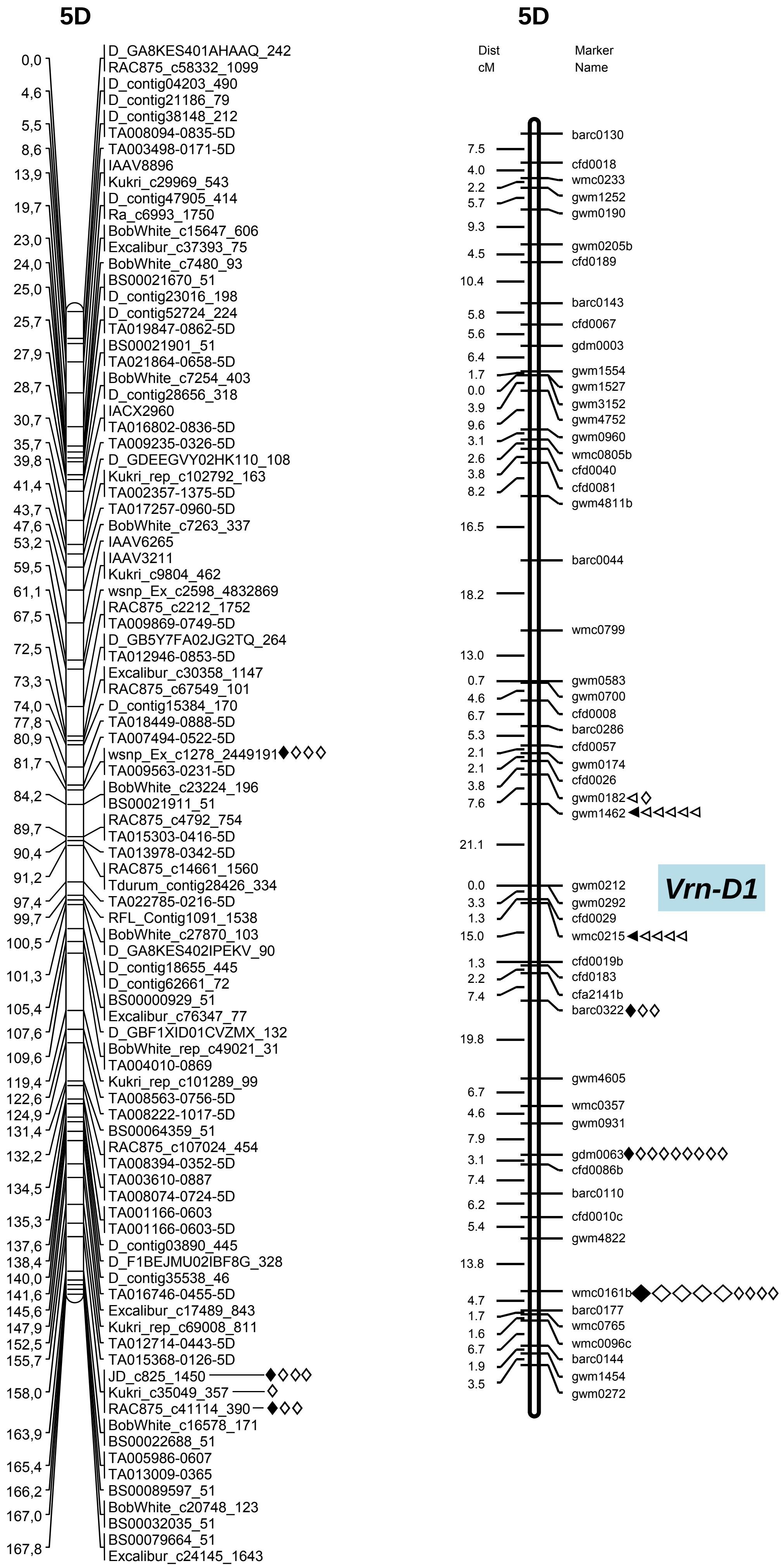
5D
0,0
4,6
5,5
8,6
13,9
19,7
23,0
24,0
25,0
25,7
27,9
28,7
30,7
35,7
39,8
41,4
43,7
47,6
53,2
59,5
61,1
67,5
72,5
73,3
74,0
77,8
80,9
81,7
84,2
89,7
90,4
91,2
97,4
99,7
100,5
101,3
105,4
107,6
109,6
119,4
122,6
124,9
131,4
132,2
134,5
135,3
137,6
138,4
140,0
141,6
145,6
147,9
152,5
155,7
158,0
163,9
165,4
166,2
167,0
167,8
D_GA8KES401AHAAQ_242
RAC875_c58332_1099
D_contig04203_490
D_contig21186_79
D_contig38148_212
TA008094-0835-5D
TA003498-0171-5D
IAAV8896
Kukri_c29969_543
D_contig47905_414
Ra_c6993_1750
BobWhite_c15647_606
Excalibur_c37393_75
BobWhite_c7480_93
BS00021670_51
D_contig23016_198
D_contig52724_224
TA019847-0862-5D
BS00021901_51
TA021864-0658-5D
BobWhite_c7254_403
D_contig28656_318
IACX2960
TA016802-0836-5D
TA009235-0326-5D
D_GDEEGVY02HK110_108
Kukri_rep_c102792_163
TA002357-1375-5D
TA017257-0960-5D
BobWhite_c7263_337
IAAV6265
IAAV3211
Kukri_c9804_462
wsnp_Ex_c2598_4832869
RAC875_c2212_1752
TA009869-0749-5D
D_GB5Y7FA02JG2TQ_264
TA012946-0853-5D
Excalibur_c30358_1147
RAC875_c67549_101
D_contig15384_170
TA018449-0888-5D
TA007494-0522-5D
wsnp_Ex_c1278_2449191
TA009563-0231-5D
BobWhite_c23224_196
BS00021911_51
RAC875_c4792_754
TA015303-0416-5D
TA013978-0342-5D
RAC875_c14661_1560
Tdurum_contig28426_334
TA022785-0216-5D
RFL_Contig1091_1538
BobWhite_c27870_103
D_GA8KES402IPEKV_90
D_contig18655_445
D_contig62661_72
BS00000929_51
Excalibur_c76347_77
D_GBF1XID01CVZMX_132
BobWhite_rep_c49021_31
TA004010-0869
Kukri_rep_c101289_99
TA008563-0756-5D
TA008222-1017-5D
BS00064359_51
RAC875_c107024_454
TA008394-0352-5D
TA003610-0887
TA008074-0724-5D
TA001166-0603
TA001166-0603-5D
D_contig03890_445
D_F1BEJMU02IBF8G_328
D_contig35538_46
TA016746-0455-5D
Excalibur_c17489_843
Kukri_rep_c69008_811
TA012714-0443-5D
TA015368-0126-5D
JD_c825_1450
Kukri_c35049_357
RAC875_c41114_390
BobWhite_c16578_171
BS00022688_51
TA005986-0607
TA013009-0365
BS00089597_51
BobWhite_c20748_123
BS00032035_51
BS00079664_51
Excalibur_c24145_1643
5D
Dist
cM
Marker
Name
barc0130
7.5
4.0
cfd0018
2.2
wmc0233
5.7
gwm1252
9.3
gwm0190
4.5
gwm0205b
10.4
cfd0189
5.8
barc0143
5.6
cfd0067
6.4
gdm0003
1.7
gwm1554
0.0
gwm1527
3.9
gwm3152
9.6
gwm4752
3.1
gwm0960
2.6
wmc0805b
3.8
cfd0040
8.2
cfd0081
gwm4811b
16.5
barc0044
18.2
wmc0799
13.0
0.7
gwm0583
4.6
gwm0700
6.7
cfd0008
5.3
barc0286
2.1
cfd0057
2.1
gwm0174
3.8
cfd0026
7.6
gwm0182
gwm1462
21.1
Vrn-D1
0.0
gwm0212
3.3
gwm0292
1.3
cfd0029
15.0
wmc0215
1.3
cfd0019b
2.2
cfd0183
7.4
cfa2141b
barc0322
19.8
gwm4605
6.7
wmc0357
4.6
gwm0931
7.9
gdm0063
3.1
cfd0086b
7.4
barc0110
6.2
cfd0010c
5.4
gwm4822
13.8
wmc0161b
4.7
barc0177
1.7
wmc0765
1.6
wmc0096c
6.7
barc0144
1.9
gwm1454
3.5
gwm0272

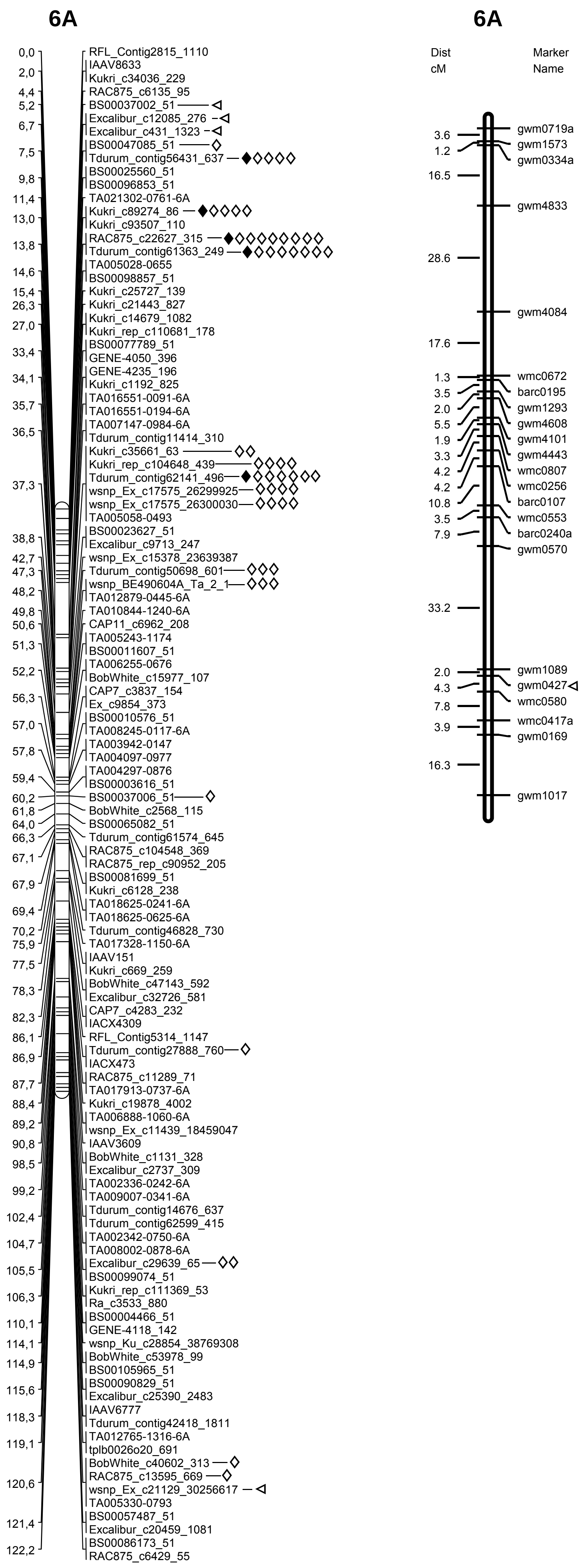

6A
0,0
2,0
4,4
5,2
6,7
7,5
9,8
11,4
13,0
13,8
14,6
15,4
26,3
27,0
33,4
34,1
35,7
36,5
37,3
38,8
42,7
47,3
48,2
49,8
50,6
51,3
52,2
56,3
57,0
57,8
59,4
60,2
61,8
64,0
66,3
67,1
67,9
69,4
70,2
75,9
77,5
78,3
82,3
86,1
86,9
87,7
88,4
89,2
90,8
98,5
99,2
102,4
104,7
105,5
106,3
110,1
114,1
114,9
115,6
118,3
119,1
120,6
121,4
122,2
RFL_Contig2815_1110
IAAV8633
Kukri_c34036_229
RAC875_c6135_95
BS00037002_51
Excalibur_c12085_276
Excalibur_c431_1323
BS00047085_51
Tdurum_contig56431_637
BS00025560_51
BS00096853_51
TA021302-0761-6A
Kukri_c89274_86
Kukri_c93507_110
RAC875_c22627_315
Tdurum_contig61363_249
TA005028-0655
BS00098857_51
Kukri_c25727_139
Kukri_c21443_827
Kukri_c14679_1082
Kukri_rep_c110681_178
BS00077789_51
GENE-4050_396
GENE-4235_196
Kukri_c1192_825
TA016551-0091-6A
TA016551-0194-6A
TA007147-0984-6A
Tdurum_contig11414_310
Kukri_c35661_63
Kukri_rep_c104648_439
Tdurum_contig62141_496
wsnp_Ex_c17575_26299925
wsnp_Ex_c17575_26300030
TA005058-0493
BS00023627_51
Excalibur_c9713_247
wsnp_Ex_c15378_23639387
Tdurum_contig50698_601
wsnp_BE490604A_Ta_2_1
TA012879-0445-6A
TA010844-1240-6A
CAP11_c6962_208
TA005243-1174
BS00011607_51
TA006255-0676
BobWhite_c15977_107
CAP7_c3837_154
Ex_c9854_373
BS00010576_51
TA008245-0117-6A
TA003942-0147
TA004097-0977
TA004297-0876
BS00003616_51
BS00037006_51
BobWhite_c2568_115
BS00065082_51
Tdurum_contig61574_645
RAC875_c104548_369
RAC875_rep_c90952_205
BS00081699_51
Kukri_c6128_238
TA018625-0241-6A
TA018625-0625-6A
Tdurum_contig46828_730
TA017328-1150-6A
IAAV151
Kukri_c669_259
BobWhite_c47143_592
Excalibur_c32726_581
CAP7_c4283_232
IACX4309
RFL_Contig5314_1147
Tdurum_contig27888_760
IACX473
RAC875_c11289_71
TA017913-0737-6A
Kukri_c19878_4002
TA006888-1060-6A
wsnp_Ex_c11439_18459047
IAAV3609
BobWhite_c1131_328
Excalibur_c2737_309
TA002336-0242-6A
TA009007-0341-6A
Tdurum_contig14676_637
Tdurum_contig62599_415
TA002342-0750-6A
TA008002-0878-6A
Excalibur_c29639_65
BS00099074_51
Kukri_rep_c111369_53
Ra_c3533_880
BS00004466_51
GENE-4118_142
wsnp_Ku_c28854_38769308
BobWhite_c53978_99
BS00105965_51
BS00090829_51
Excalibur_c25390_2483
IAAV6777
Tdurum_contig42418_1811
TA012765-1316-6A
tplb0026o20_691
BobWhite_c40602_313
RAC875_c13595_669
wsnp_Ex_c21129_30256617
TA005330-0793
BS00057487_51
Excalibur_c20459_1081
BS00086173_51
RAC875_c6429_55
6A
Dist
cM
Marker
Name
3.6
1.2
16.5
28.6
17.6
1.3
3.5
2.0
5.5
1.9
3.3
4.2
4.2
10.8
3.5
7.9
33.2
2.0
4.3
7.8
3.9
16.3
gwm0719a
gwm1573
gwm0334a
gwm4833
gwm4084
wmc0672
barc0195
gwm1293
gwm4608
gwm4101
gwm4443
wmc0807
wmc0256
barc0107
wmc0553
barc0240a
gwm0570
gwm1089
gwm0427
wmc0580
wmc0417a
gwm0169
gwm1017

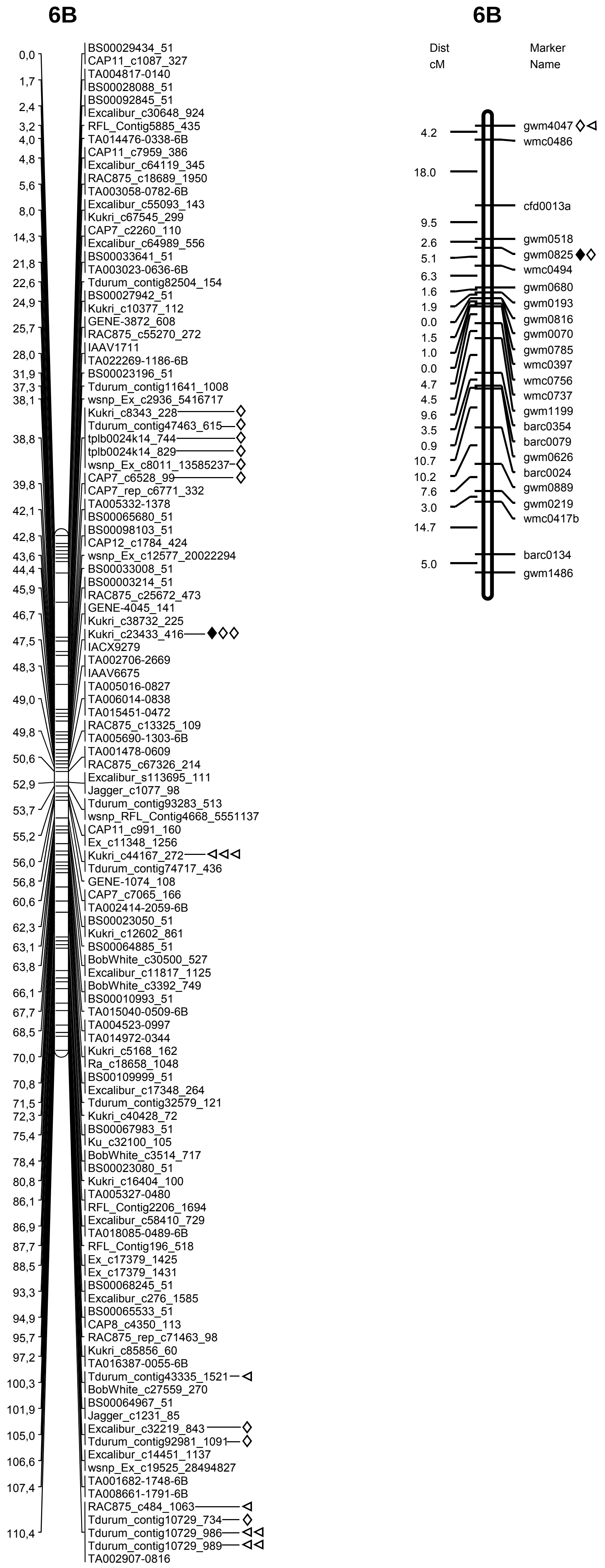

6B
0,0
1,7
2,4
3,2
4,0
4,8
5,6
8,0
14,3
21,8
22,6
24,9
25,7
28,0
31,9
37,3
38,1
38,8
39,8
42,1
42,8
43,6
44,4
45,9
46,7
47,5
48,3
49,0
49,8
50,6
52,9
53,7
55,2
56,0
56,8
60,6
62,3
63,1
63,8
66,1
67,7
68,5
70,0
70,8
71,5
72,3
75,4
78,4
80,8
86,1
86,9
87,7
88,5
93,3
94,9
95,7
97,2
100,3
101,9
105,0
106,6
107,4
110,4
BS00029434_51
CAP11_c1087_327
TA004817-0140
BS00028088_51
BS00092845_51
Excalibur_c30648_924
RFL_Contig5885_435
TA014476-0338-6B
CAP11_c7959_386
Excalibur_c64119_345
RAC875_c18689_1950
TA003058-0782-6B
Excalibur_c55093_143
Kukri_c67545_299
CAP7_c2260_110
Excalibur_c64989_556
BS00033641_51
TA003023-0636-6B
Tdurum_contig82504_154
BS00027942_51
Kukri_c10377_112
GENE-3872_608
RAC875_c55270_272
IAAV1711
TA022269-1186-6B
BS00023196_51
Tdurum_contig11641_1008
wsnp_Ex_c2936_5416717
Kukri_c8343_228
Tdurum_contig47463_615
tplb0024k14_744
tplb0024k14_829
wsnp_Ex_c8011_13585237
CAP7_c6528_99
CAP7_rep_c6771_332
TA005332-1378
BS00065680_51
BS00098103_51
CAP12_c1784_424
wsnp_Ex_c12577_20022294
BS00033008_51
BS00003214_51
RAC875_c25672_473
GENE-4045_141
Kukri_c38732_225
Kukri_c23433_416
IACX9279
TA002706-2669
IAAV6675
TA005016-0827
TA006014-0838
TA015451-0472
RAC875_c13325_109
TA005690-1303-6B
TA001478-0609
RAC875_c67326_214
Excalibur_s113695_111
Jagger_c1077_98
Tdurum_contig93283_513
wsnp_RFL_Contig4668_5551137
CAP11_c991_160
Ex_c11348_1256
Kukri_c44167_272
Tdurum_contig74717_436
GENE-1074_108
CAP7_c7065_166
TA002414-2059-6B
BS00023050_51
Kukri_c12602_861
BS00064885_51
BobWhite_c30500_527
Excalibur_c11817_1125
BobWhite_c3392_749
BS00010993_51
TA015040-0509-6B
TA004523-0997
TA014972-0344
Kukri_c5168_162
Ra_c18658_1048
BS00109999_51
Excalibur_c17348_264
Tdurum_contig32579_121
Kukri_c40428_72
BS00067983_51
Ku_c32100_105
BobWhite_c3514_717
BS00023080_51
Kukri_c16404_100
TA005327-0480
RFL_Contig2206_1694
Excalibur_c58410_729
TA018085-0489-6B
RFL_Contig196_518
Ex_c17379_1425
Ex_c17379_1431
BS00068245_51
Excalibur_c276_1585
BS00065533_51
CAP8_c4350_113
RAC875_rep_c71463_98
Kukri_c85856_60
TA016387-0055-6B
Tdurum_contig43335_1521
BobWhite_c27559_270
BS00064967_51
Jagger_c1231_85
Excalibur_c32219_843
Tdurum_contig92981_1091
Excalibur_c14451_1137
wsnp_Ex_c19525_28494827
TA001682-1748-6B
TA008661-1791-6B
RAC875_c484_1063
Tdurum_contig10729_734
Tdurum_contig10729_986
Tdurum_contig10729_989
TA002907-0816
6B
Dist
cM
Marker
Name
4.2
18.0
9.5
2.6
5.1
6.3
1.6
1.9
0.0
1.5
1.0
0.0
4.7
4.5
9.6
3.5
0.9
10.7
10.2
7.6
3.0
14.7
5.0
gwm4047
wmc0486
cfd0013a
gwm0518
gwm0825
wmc0494
gwm0680
gwm0193
gwm0816
gwm0070
gwm0785
wmc0397
wmc0756
wmc0737
gwm1199
barc0354
barc0079
gwm0626
barc0024
gwm0889
gwm0219
wmc0417b
barc0134
gwm1486

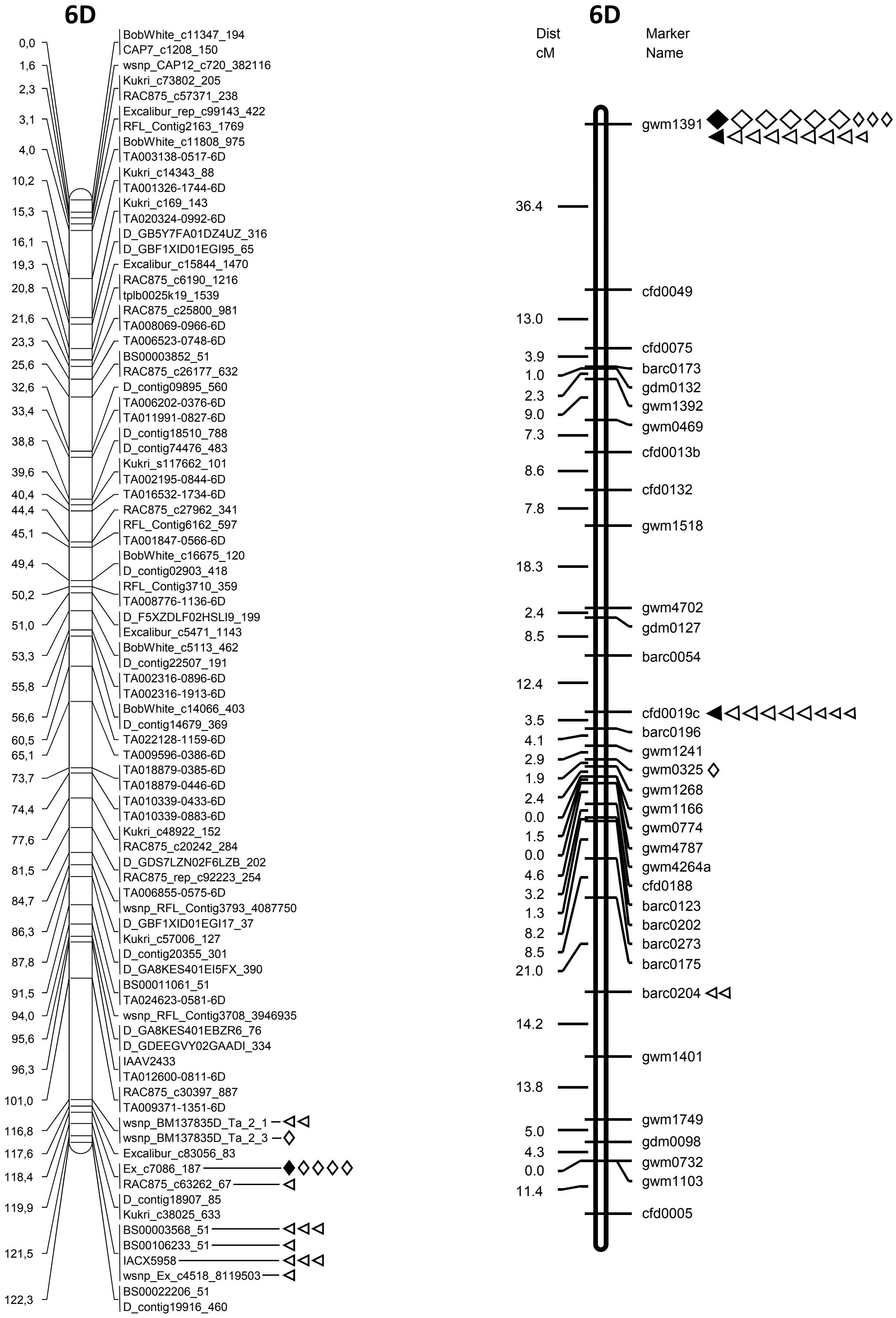

6D
0,0
1,6
2,3
3,1
4,0
10,2
15,3
16,1
19,3
20,8
21,6
23,3
25,6
32,6
33,4
38,8
39,6
40,4
44,4
45,1
49,4
50,2
51,0
53,3
55,8
56,6
60,5
65,1
73,7
74,4
77,6
81,5
84,7
86,3
87,8
91,5
94,0
95,6
96,3
101,0
116,8
117,6
118,4
119,9
121,5
122,3
BobWhite_c11347_194
CAP7_c1208_150
wsnp_CAP12_c720_382116
Kukri_c73802_205
RAC875_c57371_238
Excalibur_rep_c99143_422
RFL_Contig2163_1769
BobWhite_c11808_975
TA003138-0517-6D
Kukri_c14343_88
TA001326-1744-6D
Kukri_c169_143
TA020324-0992-6D
D_GB5Y7FA01DZ4UZ_316
D_GBF1XID01EGI95_65
Excalibur_c15844_1470
RAC875_c6190_1216
tplb0025k19_1539
RAC875_c25800_981
TA008069-0966-6D
TA006523-0748-6D
BS00003852_51
RAC875_c26177_632
D_contig09895_560
TA006202-0376-6D
TA011991-0827-6D
D_contig18510_788
D_contig74476_483
Kukri_s117662_101
TA002195-0844-6D
TA016532-1734-6D
RAC875_c27962_341
RFL_Contig6162_597
TA001847-0566-6D
BobWhite_c16675_120
D_contig02903_418
RFL_Contig3710_359
TA008776-1136-6D
D_F5XZDLF02HSLI9_199
Excalibur_c5471_1143
BobWhite_c5113_462
D_contig22507_191
TA002316-0896-6D
TA002316-1913-6D
BobWhite_c14066_403
D_contig14679_369
TA022128-1159-6D
TA009596-0386-6D
TA018879-0385-6D
TA018879-0446-6D
TA010339-0433-6D
TA010339-0883-6D
Kukri_c48922_152
RAC875_c20242_284
D_GDS7LZN02F6LZB_202
RAC875_rep_c92223_254
TA006855-0575-6D
wsnp_RFL_Contig3793_4087750
D_GBF1XID01EGI17_37
Kukri_c57006_127
D_contig20355_301
D_GA8KES401EI5FX_390
BS00011061_51
TA024623-0581-6D
wsnp_RFL_Contig3708_3946935
D_GA8KES401EBZR6_76
D_GDEEGVY02GAADI_334
IAAV2433
TA012600-0811-6D
RAC875_c30397_887
TA009371-1351-6D
wsnp_BM137835D_Ta_2_1
wsnp_BM137835D_Ta_2_3
Excalibur_c83056_83
Ex_c7086_187
RAC875_c63262_67
D_contig18907_85
Kukri_c38025_633
BS00003568_51
BS00106233_51
IACX5958
wsnp_Ex_c4518_8119503
BS00022206_51
D_contig19916_460
6D
Dist cM
Marker Name
36.4
13.0
3.9
1.0
2.3
9.0
7.3
8.6
7.8
18.3
2.4
8.5
12.4
3.5
4.1
2.9
1.9
2.4
0.0
1.5
0.0
4.6
3.2
1.3
8.2
8.5
21.0
14.2
13.8
5.0
4.3
0.0
11.4
gwm1391
cfd0049
cfd0075
barc0173
gdm0132
gwm1392
gwm0469
cfd0013b
cfd0132
gwm1518
gwm4702
gdm0127
barc0054
cfd0019c
barc0196
gwm1241
gwm0325
gwm1268
gwm1166
gwm0774
gwm4787
gwm4264a
cfd0188
barc0123
barc0202
barc0273
barc0175
barc0204
gwm1401
gwm1749
gdm0098
gwm0732
gwm1103
cfd0005

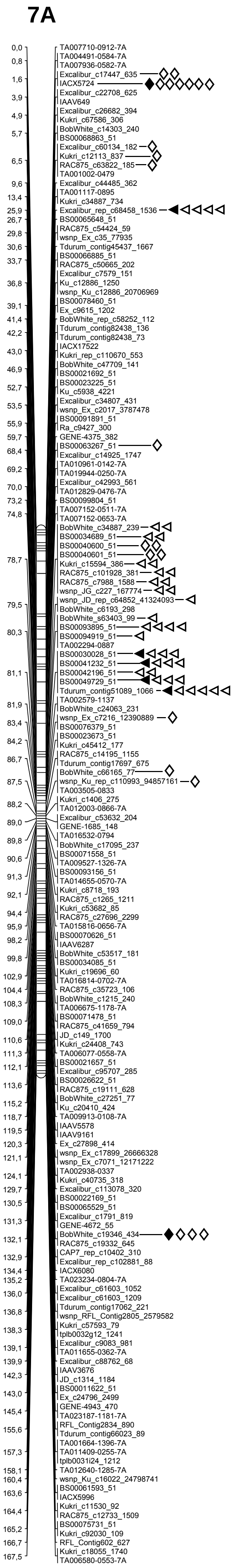
7A
0,0
0,8
1,6
3,9
4,9
5,7
6,5
9,6
13,4
25,9
26,7
29,8
30,6
33,7
36,8
39,1
41,4
42,2
43,0
46,9
52,7
53,5
55,9
59,7
68,4
69,2
70,0
73,2
74,8
78,7
79,5
80,3
81,1
81,9
83,4
84,2
86,7
87,5
88,2
89,0
89,8
90,6
91,3
92,1
94,4
95,9
98,2
99,8
102,9
104,4
108,3
109,0
110,6
111,3
112,1
113,6
115,2
118,7
119,5
120,3
121,1
124,1
129,7
130,5
131,3
132,1
132,9
134,4
135,2
136,0
136,8
138,3
139,1
139,9
142,3
143,0
145,4
155,6
157,3
158,1
160,4
163,6
164,4
165,2
166,7
167,5
TA007710-0912-7A
TA004491-0584-7A
TA007936-0582-7A
Excalibur_c17447_635
IACX5724
Excalibur_c22708_625
IAAV649
Excalibur_c26682_394
Kukri_c67586_306
BobWhite_c14303_240
BS00068863_51
Excalibur_c60134_182
Kukri_c12113_837
RAC875_c63822_185
TA001002-0479
Excalibur_c44485_362
TA001117-0895
Kukri_c34887_734
Excalibur_rep_c68458_1536
BS00065648_51
RAC875_c54424_59
wsnp_Ex_c35_77935
Tdurum_contig45437_1667
BS00066885_51
RAC875_c50665_202
Excalibur_c7579_151
Ku_c12886_1250
wsnp_Ku_c12886_20706969
BS00078460_51
Ex_c9615_1202
BobWhite_rep_c58252_112
Tdurum_contig82438_136
Tdurum_contig82438_73
IACX17522
Kukri_rep_c110670_553
BobWhite_c47709_141
BS00021692_51
BS00023225_51
Ku_c5938_4221
Excalibur_c34807_431
wsnp_Ex_c2017_3787478
BS00091891_51
Ra_c9427_300
GENE-4375_382
BS00063267_51
Excalibur_c14925_1747
TA010961-0142-7A
TA019944-0250-7A
Excalibur_c42993_561
TA012829-0476-7A
BS00099804_51
TA007152-0511-7A
TA007152-0653-7A
BobWhite_c34887_239
BS00034689_51
BS00040600_51
BS00040601_51
Kukri_c15594_386
RAC875_c101928_381
RAC875_c7988_1588
wsnp_JG_c227_167774
wsnp_JD_rep_c64852_41324093
BobWhite_c6193_298
BobWhite_s63403_99
BS00093895_51
BS00094919_51
TA002294-0887
BS00030028_51
BS00041232_51
BS00042196_51
BS00049729_51
Tdurum_contig51089_1066
TA002579-1137
BobWhite_c24063_231
wsnp_Ex_c7216_12390889
BS00076379_51
BS00023673_51
Kukri_c45412_177
RAC875_c14195_1155
Tdurum_contig17697_675
BobWhite_c66165_77
wsnp_Ku_rep_c110993_94857161
TA003505-0833
Kukri_c1406_275
TA012003-0866-7A
Excalibur_c53632_204
GENE-1685_148
TA016532-0794
BobWhite_c17095_237
BS00071558_51
TA009527-1326-7A
BS00093156_51
TA014655-0570-7A
Kukri_c8718_193
RAC875_c1265_1211
Kukri_c53682_85
RAC875_c27696_2299
TA015816-0656-7A
BS00070626_51
IAAV6287
BobWhite_c53517_181
BS00034085_51
Kukri_c19696_60
TA016814-0702-7A
RAC875_c35723_106
BobWhite_c1215_240
TA006675-1178-7A
BS00071478_51
RAC875_c41659_794
JD_c149_1700
Kukri_c24408_743
TA006077-0558-7A
BS00021657_51
Excalibur_c95707_285
BS00026622_51
RAC875_c19111_628
BobWhite_c27251_77
Ku_c20410_424
TA009913-0108-7A
IAAV5578
IAAV9161
Ex_c27898_414
wsnp_Ex_c17899_26666328
wsnp_Ex_c7071_12171222
TA002938-0337
Kukri_c40735_318
Excalibur_c113078_320
BS00022169_51
BS00065529_51
Excalibur_c1791_819
GENE-4672_55
BobWhite_c19346_434
RAC875_c19332_645
CAP7_rep_c10402_310
Excalibur_rep_c102881_88
IACX6080
TA023234-0804-7A
Excalibur_c61603_1052
Excalibur_c61603_1209
Tdurum_contig17062_221
wsnp_RFL_Contig2805_2579582
Kukri_c57593_79
tplb0032g12_1241
Excalibur_c9083_981
TA011655-0362-7A
Excalibur_c88762_68
IAAV3676
JD_c1314_1184
BS00011622_51
Ex_c24796_2499
GENE-4943_470
TA023187-1181-7A
RFL_Contig2834_890
Tdurum_contig66023_89
TA001664-1396-7A
TA011409-0255-7A
tplb0031i24_1212
TA012640-1285-7A
wsnp_Ku_c16022_24798741
BS00061593_51
IACX5996
Kukri_c11530_92
RAC875_c12733_1509
BS00075731_51
Kukri_c92030_109
RFL_Contig602_627
Kukri_c18055_1740
TA006580-0553-7A

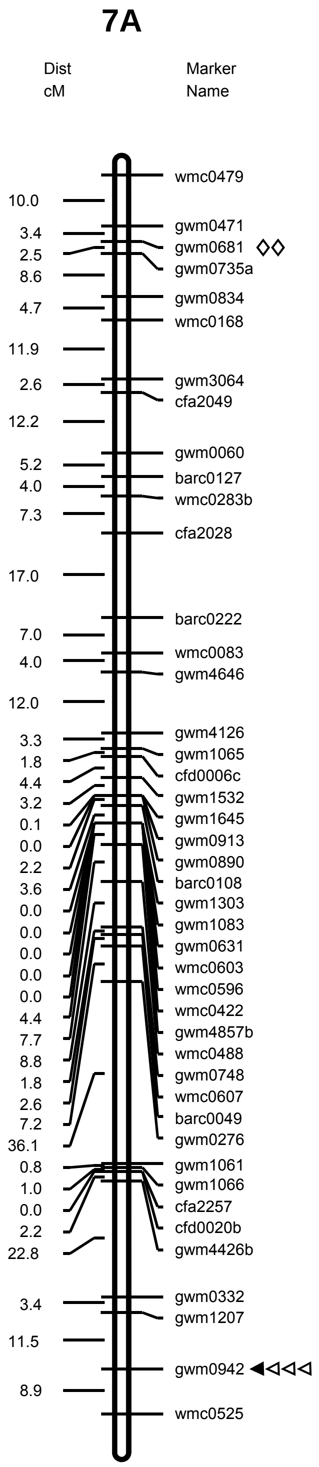
7A
Dist
cM
Marker
Name
10.0
3.4
2.5
8.6
4.7
11.9
2.6
12.2
5.2
4.0
7.3
17.0
7.0
4.0
12.0
3.3
1.8
4.4
3.2
0.1
0.0
2.2
3.6
0.0
0.0
0.0
0.0
0.0
4.4
7.7
8.8
1.8
2.6
7.2
36.1
0.8
1.0
0.0
2.2
22.8
3.4
11.5
8.9
wmc0479
gwm0471
gwm0681
gwm0735a
gwm0834
wmc0168
gwm3064
cfa2049
gwm0060
barc0127
wmc0283b
cfa2028
barc0222
wmc0083
gwm4646
gwm4126
gwm1065
cfd0006c
gwm1532
gwm1645
gwm0913
gwm0890
barc0108
gwm1303
gwm1083
gwm0631
wmc0603
wmc0596
wmc0422
gwm4857b
wmc0488
gwm0748
wmc0607
barc0049
gwm0276
gwm1061
gwm1066
cfa2257
cfd0020b
gwm4426b
gwm0332
gwm1207
gwm0942
wmc0525

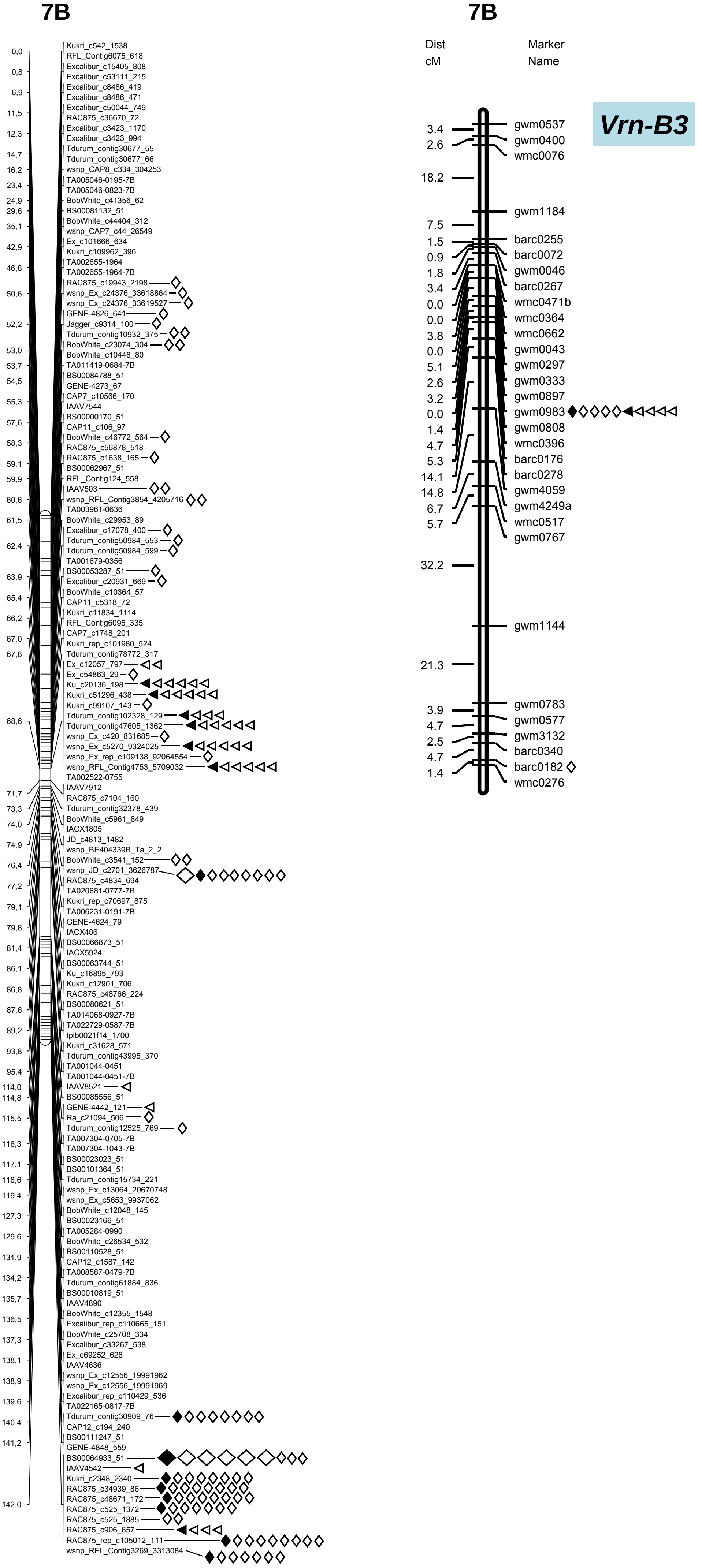

7B
0,0
0,8
6,9
11,5
12,3
14,7
16,2
23,4
24,9
29,6
35,1
42,9
46,8
50,6
52,2
53,0
53,7
54,5
55,3
57,6
58,3
59,1
59,9
60,6
61,5
62,4
63,9
65,4
66,2
67,0
67,8
68,6
71,7
73,3
74,0
74,9
76,4
77,2
79,1
79,8
81,4
86,1
86,8
87,6
89,2
93,8
95,4
114,0
114,8
115,5
116,3
117,1
118,6
119,4
127,3
129,6
131,9
134,2
135,7
136,5
137,3
138,1
138,9
139,6
140,4
141,2
142,0
Kukri_c542_1538
RFL_Contig6075_618
Excalibur_c15405_808
Excalibur_c53111_215
Excalibur_c8486_419
Excalibur_c8486_471
Excalibur_c50044_749
RAC875_c36670_72
Excalibur_c3423_1170
Excalibur_c3423_994
Tdurum_contig30677_55
Tdurum_contig30677_66
wsnp_CAP8_c334_304253
TA005046-0195-7B
TA005046-0823-7B
BobWhite_c41356_62
BS00081132_51
BobWhite_c44404_312
wsnp_CAP7_c44_26549
Ex_c101666_634
Kukri_c109962_396
TA002655-1964
TA002655-1964-7B
RAC875_c19943_2198
wsnp_Ex_c24376_33618864
wsnp_Ex_c24376_33619527
GENE-4826_641
Jagger_c9314_100
Tdurum_contig10932_375
BobWhite_c23074_304
BobWhite_c10448_80
TA011419-0684-7B
BS00084788_51
GENE-4273_67
CAP7_c10566_170
IAAV7544
BS00000170_51
CAP11_c106_97
BobWhite_c46772_564
RAC875_c56878_518
RAC875_c1638_165
BS00062967_51
RFL_Contig124_558
IAAV503
wsnp_RFL_Contig3854_4205716
TA003961-0636
BobWhite_c29953_89
Excalibur_c17078_400
Tdurum_contig50984_553
Tdurum_contig50984_599
TA001679-0356
BS00053287_51
Excalibur_c20931_669
BobWhite_c10364_57
CAP11_c5318_72
Kukri_c11834_1114
RFL_Contig6095_335
CAP7_c1748_201
Kukri_rep_c101980_524
Tdurum_contig78772_317
Ex_c12057_797
Ex_c54863_29
Ku_c20136_198
Kukri_c51296_438
Kukri_c99107_143
Tdurum_contig102328_129
Tdurum_contig47605_1362
wsnp_Ex_c420_831685
wsnp_Ex_c5270_9324025
wsnp_Ex_rep_c109138_92064554
wsnp_RFL_Contig4753_5709032
TA002522-0755
IAAV7912
RAC875_c7104_160
Tdurum_contig32378_439
BobWhite_c5961_849
IACX1805
JD_c4813_1482
wsnp_BE404339B_Ta_2_2
BobWhite_c3541_152
wsnp_JD_c2701_3626787
RAC875_c4834_694
TA020681-0777-7B
Kukri_rep_c70697_875
TA006231-0191-7B
GENE-4624_79
IACX486
BS00066873_51
IACX5924
BS00063744_51
Ku_c16895_793
Kukri_c12901_706
RAC875_c48766_224
BS00080621_51
TA014068-0927-7B
TA022729-0587-7B
tplb0021f14_1700
Kukri_c31628_571
Tdurum_contig43995_370
TA001044-0451
TA001044-0451-7B
IAAV8521
BS00085556_51
GENE-4442_121
Ra_c21094_506
Tdurum_contig12525_769
TA007304-0705-7B
TA007304-1043-7B
BS00023023_51
BS00101364_51
Tdurum_contig15734_221
wsnp_Ex_c13064_20670748
wsnp_Ex_c5653_9937062
BobWhite_c12048_145
BS00023166_51
TA005284-0990
BobWhite_c26534_532
BS00110528_51
CAP12_c1587_142
TA008587-0479-7B
Tdurum_contig61884_836
BS00010819_51
IAAV4890
BobWhite_c12355_1548
Excalibur_rep_c110665_151
BobWhite_c25708_334
Excalibur_c33267_538
Ex_c69252_628
IAAV4636
wsnp_Ex_c12556_19991962
wsnp_Ex_c12556_19991969
Excalibur_rep_c110429_536
TA022165-0817-7B
Tdurum_contig30909_76
CAP12_c194_240
BS00111247_51
GENE-4848_559
BS00064933_51
IAAV4542
Kukri_c2348_2340
RAC875_c34939_86
RAC875_c48671_172
RAC875_c525_1372
RAC875_c525_1885
RAC875_c906_657
RAC875_rep_c105012_111
wsnp_RFL_Contig3269_3313084
7B
Dist
cM
Marker
Name
Vrn-B3
3.4
2.6
18.2
7.5
1.5
0.9
1.8
3.4
0.0
0.0
3.8
0.0
5.1
2.6
3.2
0.0
1.4
4.7
5.3
14.1
14.8
6.7
5.7
32.2
21.3
3.9
4.7
2.5
4.7
1.4
gwm0537
gwm0400
wmc0076
gwm1184
barc0255
barc0072
gwm0046
barc0267
wmc0471b
wmc0364
wmc0662
gwm0043
gwm0297
gwm0333
gwm0897
gwm0983
gwm0808
wmc0396
barc0176
barc0278
gwm4059
gwm4249a
wmc0517
gwm0767
gwm1144
gwm0783
gwm0577
gwm3132
barc0340
barc0182
wmc0276

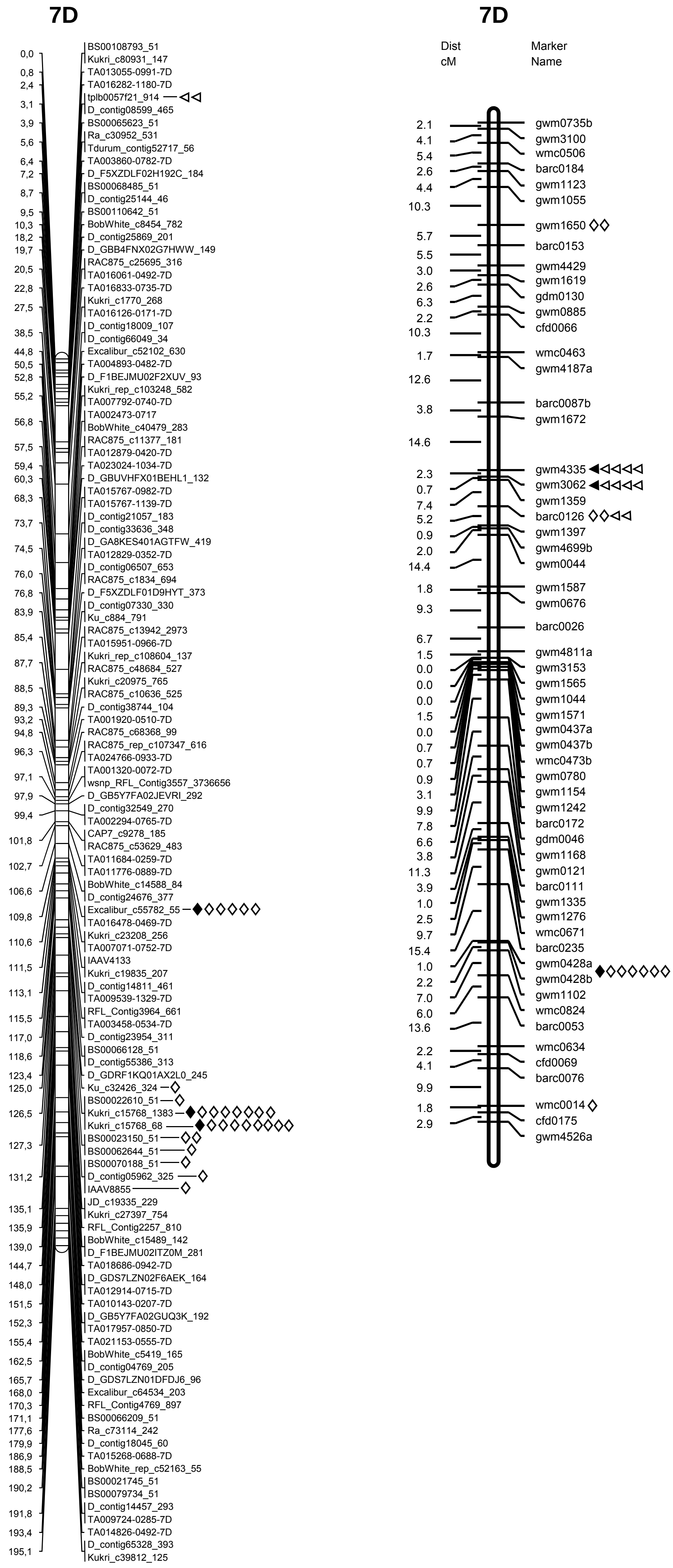
7D
0,0
0,8
2,4
3,1
3,9
5,6
6,4
7,2
8,7
9,5
10,3
18,2
19,7
20,5
22,8
27,5
38,5
44,8
50,5
52,8
55,2
56,8
57,5
59,4
60,3
68,3
73,7
74,5
76,0
76,8
83,9
85,4
87,7
88,5
89,3
93,2
94,8
96,3
97,1
97,9
99,4
101,8
102,7
106,6
109,8
110,6
111,5
113,1
115,5
117,0
118,6
123,4
125,0
126,5
127,3
131,2
135,1
135,9
139,0
144,7
148,0
151,5
152,3
155,4
162,5
165,7
168,0
170,3
171,1
177,6
179,9
186,9
188,5
190,2
191,8
193,4
195,1
BS00108793_51
Kukri_c80931_147
TA013055-0991-7D
TA016282-1180-7D
tplb0057f21_914
D_contig08599_465
BS00065623_51
Ra_c30952_531
Tdurum_contig52717_56
TA003860-0782-7D
D_F5XZDLF02H192C_184
BS00068485_51
D_contig25144_46
BS00110642_51
BobWhite_c8454_782
D_contig25869_201
D_GBB4FNX02G7HWW_149
RAC875_c25695_316
TA016061-0492-7D
TA016833-0735-7D
Kukri_c1770_268
TA016126-0171-7D
D_contig18009_107
D_contig66049_34
Excalibur_c52102_630
TA004893-0482-7D
D_F1BEJMU02F2XUV_93
Kukri_rep_c103248_582
TA007792-0740-7D
TA002473-0717
BobWhite_c40479_283
RAC875_c11377_181
TA012879-0420-7D
TA023024-1034-7D
D_GBUVHFX01BEHL1_132
TA015767-0982-7D
TA015767-1139-7D
D_contig21057_183
D_contig33636_348
D_GA8KES401AGTFW_419
TA012829-0352-7D
D_contig06507_653
RAC875_c1834_694
D_F5XZDLF01D9HYT_373
D_contig07330_330
Ku_c884_791
RAC875_c13942_2973
TA015951-0966-7D
Kukri_rep_c108604_137
RAC875_c48684_527
Kukri_c20975_765
RAC875_c10636_525
D_contig38744_104
TA001920-0510-7D
RAC875_c68368_99
RAC875_rep_c107347_616
TA024766-0933-7D
TA001320-0072-7D
wsnp_RFL_Contig3557_3736656
D_GB5Y7FA02JEVRI_292
D_contig32549_270
TA002294-0765-7D
CAP7_c9278_185
RAC875_c53629_483
TA011684-0259-7D
TA011776-0889-7D
BobWhite_c14588_84
D_contig24676_377
Excalibur_c55782_55
TA016478-0469-7D
Kukri_c23208_256
TA007071-0752-7D
IAAV4133
Kukri_c19835_207
D_contig14811_461
TA009539-1329-7D
RFL_Contig3964_661
TA003458-0534-7D
D_contig23954_311
BS00066128_51
D_contig55386_313
D_GDRF1KQ01AX2L0_245
Ku_c32426_324
BS00022610_51
Kukri_c15768_1383
Kukri_c15768_68
BS00023150_51
BS00062644_51
BS00070188_51
D_contig05962_325
IAAV8855
JD_c19335_229
Kukri_c27397_754
RFL_Contig2257_810
BobWhite_c15489_142
D_F1BEJMU02ITZ0M_281
TA018686-0942-7D
D_GDS7LZN02F6AEK_164
TA012914-0715-7D
TA010143-0207-7D
D_GB5Y7FA02GUQ3K_192
TA017957-0850-7D
TA021153-0555-7D
BobWhite_c5419_165
D_contig04769_205
D_GDS7LZN01DFDJ6_96
Excalibur_c64534_203
RFL_Contig4769_897
BS00066209_51
Ra_c73114_242
D_contig18045_60
TA015268-0688-7D
BobWhite_rep_c52163_55
BS00021745_51
BS00079734_51
D_contig14457_293
TA009724-0285-7D
TA014826-0492-7D
D_contig65328_393
Kukri_c39812_125
7D
Dist cM
Marker Name
2.1 gwm0735b
4.1 gwm3100
5.4 wmc0506
2.6 barc0184
4.4 gwm1123
10.3 gwm1055
5.7 gwm1650
5.5 barc0153
3.0 gwm4429
2.6 gwm1619
6.3 gdm0130
2.2 gwm0885
10.3 cfd0066
1.7 wmc0463
12.6 gwm4187a
3.8 barc0087b
14.6 gwm1672
2.3 gwm4335
0.7 gwm3062
7.4 gwm1359
5.2 barc0126
0.9 gwm1397
2.0 gwm4699b
14.4 gwm0044
1.8 gwm1587
9.3 gwm0676
6.7 barc0026
1.5 gwm4811a
0.0 gwm3153
0.0 gwm1565
0.0 gwm1044
1.5 gwm1571
0.0 gwm0437a
0.7 gwm0437b
0.7 wmc0473b
0.9 gwm0780
3.1 gwm1154
9.9 gwm1242
7.8 barc0172
6.6 gdm0046
3.8 gwm1168
11.3 gwm0121
3.9 barc0111
1.0 gwm1335
2.5 gwm1276
9.7 wmc0671
15.4 barc0235
1.0 gwm0428a
2.2 gwm0428b
7.0 gwm1102
6.0 wmc0824
13.6 barc0053
2.2 wmc0634
4.1 cfd0069
9.9 barc0076
1.8 wmc0014
2.9 cfd0175
gwm4526a
