# Supplementary material for: Genetic architecture of main effect QTL for heading date in European winter wheat
Source: Front Plant Sci. 2014 May 20;5:217. doi: 10.3389/fpls.2014.00217 (PMC4033046; doi:10.3389/fpls.2014.00217)
Supplement: Supplementary file 9 [file DataSheet9.PDF]

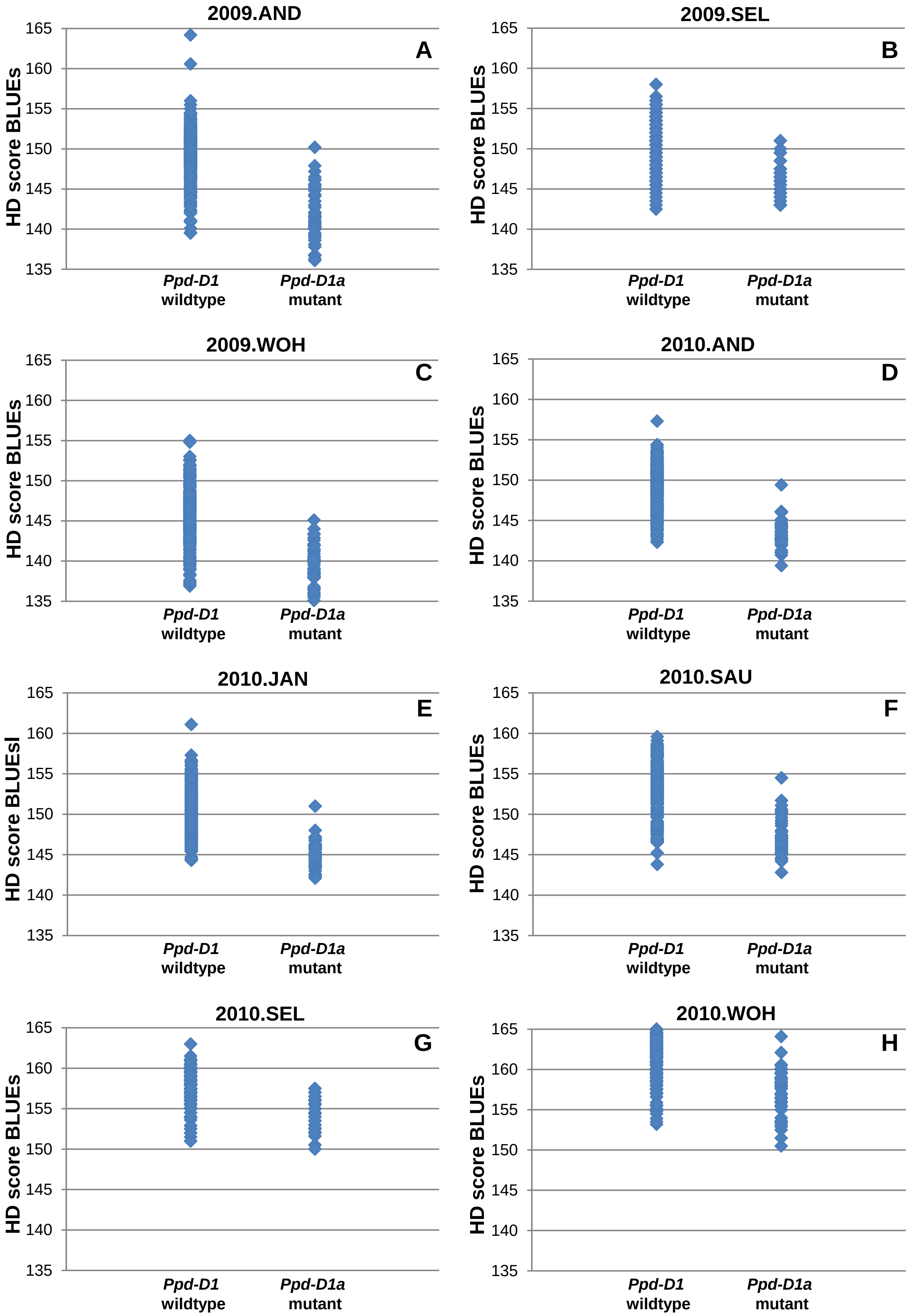

Supplemental file S9: Allelic effects for *Ppd-D1* in a population of 372 European wheat varieties.

Varieties carrying the mutant allele *Ppd-D1a* showed a decreased HD score in eight different environments resulting in an earlier heading.
