# Supplementary material for: Genetic architecture of main effect QTL for heading date in European winter wheat
Source: Front Plant Sci. 2014 May 20;5:217. doi: 10.3389/fpls.2014.00217 (PMC4033046; doi:10.3389/fpls.2014.00217)
Supplement: Supplementary file 11 [file DataSheet11.PDF]

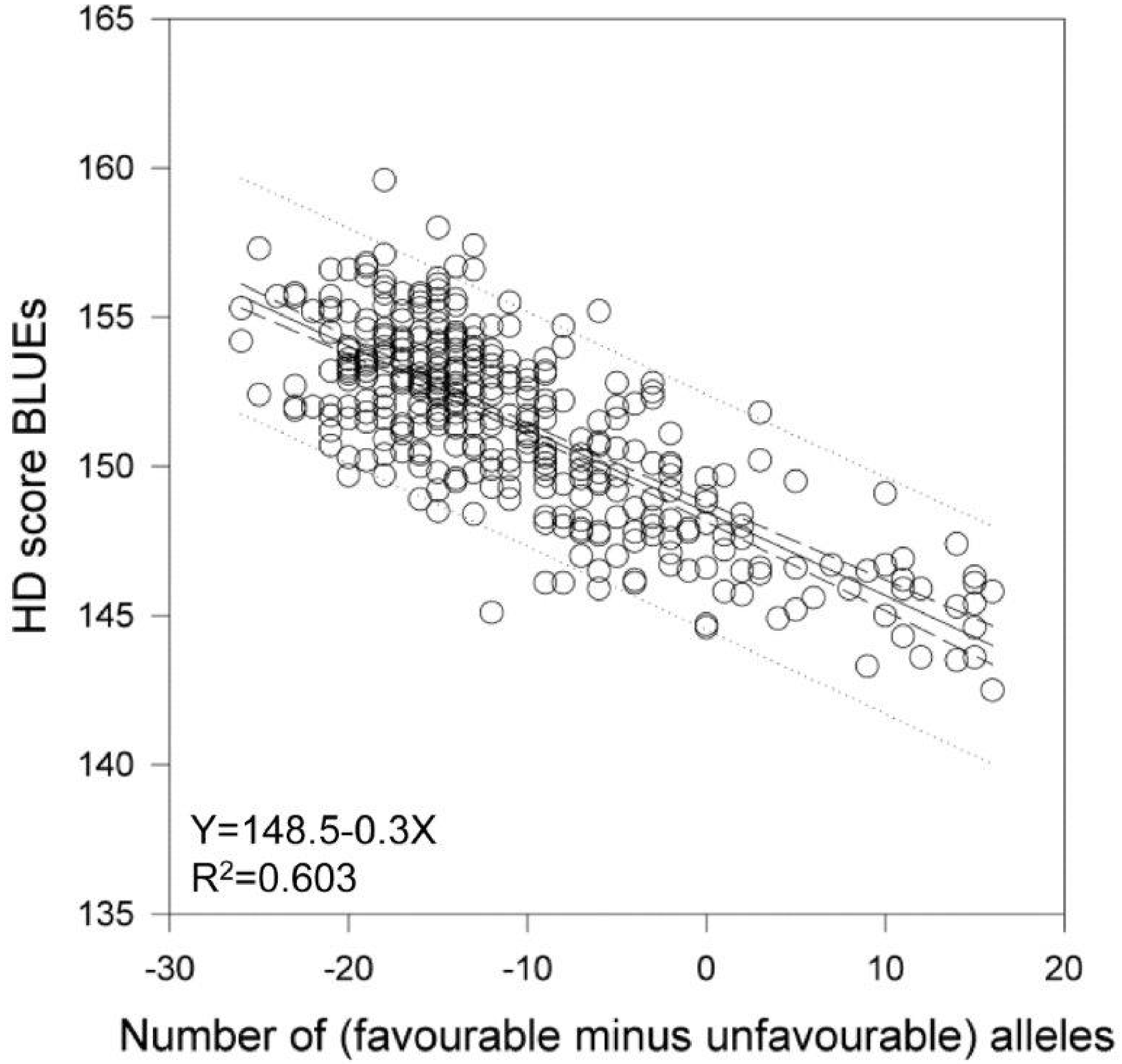

Supplement S11: Linear regression of HD score BLUEs with the number of favourable minus unfavourable alleles.
