# Supplementary material for: Genetic architecture of main effect QTL for heading date in European winter wheat
Source: Front Plant Sci. 2014 May 20;5:217. doi: 10.3389/fpls.2014.00217 (PMC4033046; doi:10.3389/fpls.2014.00217)
Supplement: Supplementary file 14 [file DataSheet14.PDF]

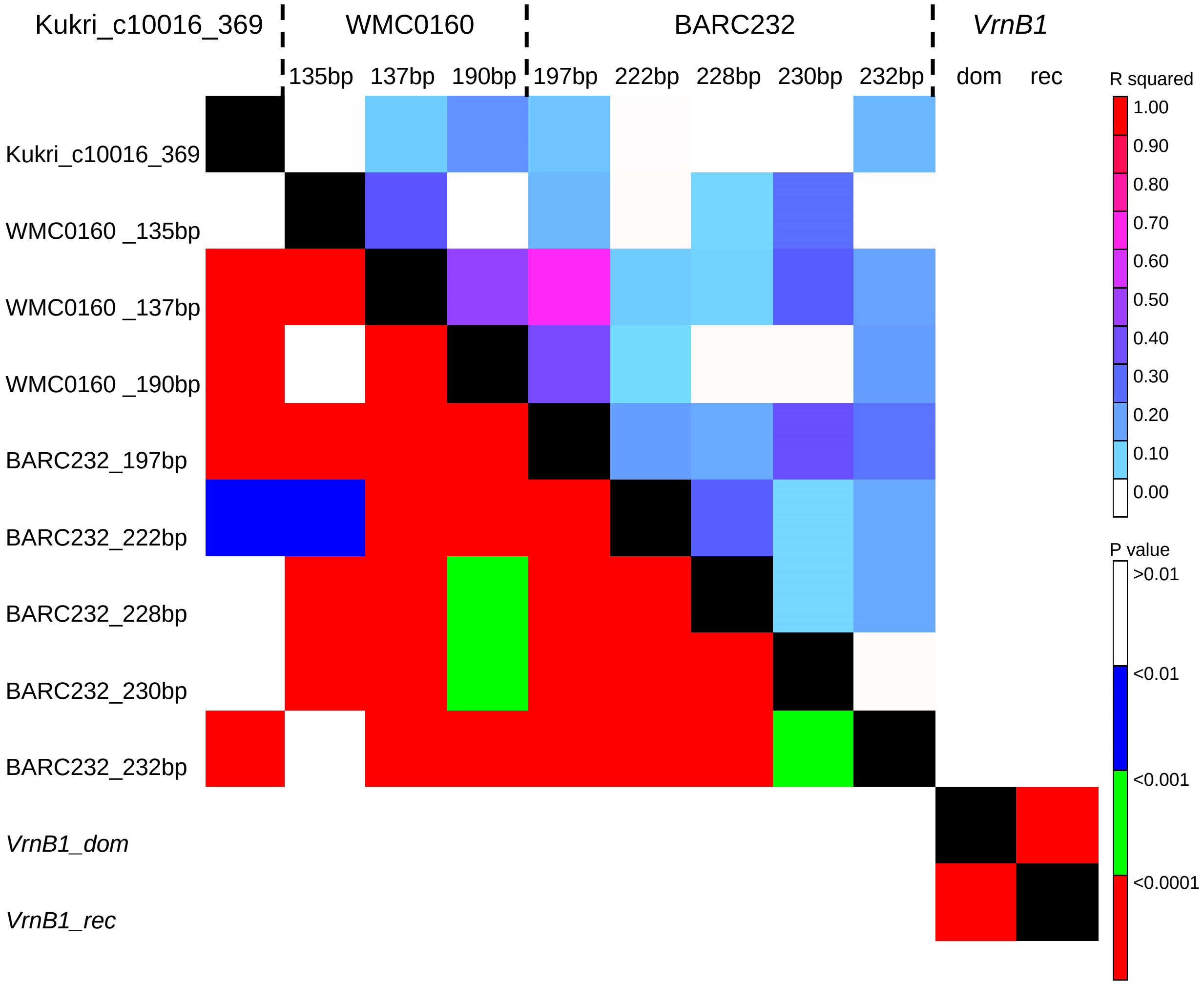

Supplemental file S14: Linkage disequilibrium was detected between SNP-marker Kukri_c10016_369 with homology to rice photoperiodism gene *Hd6* and SSR markers WMC160 and BARC232, while no homology was found to candidate gene *VrnB1*.
