# Supplementary material for: Genetic architecture of main effect QTL for heading date in European winter wheat
Source: Front Plant Sci. 2014 May 20;5:217. doi: 10.3389/fpls.2014.00217 (PMC4033046; doi:10.3389/fpls.2014.00217)
Supplement: Supplementary file 15 [file DataSheet15.PDF]

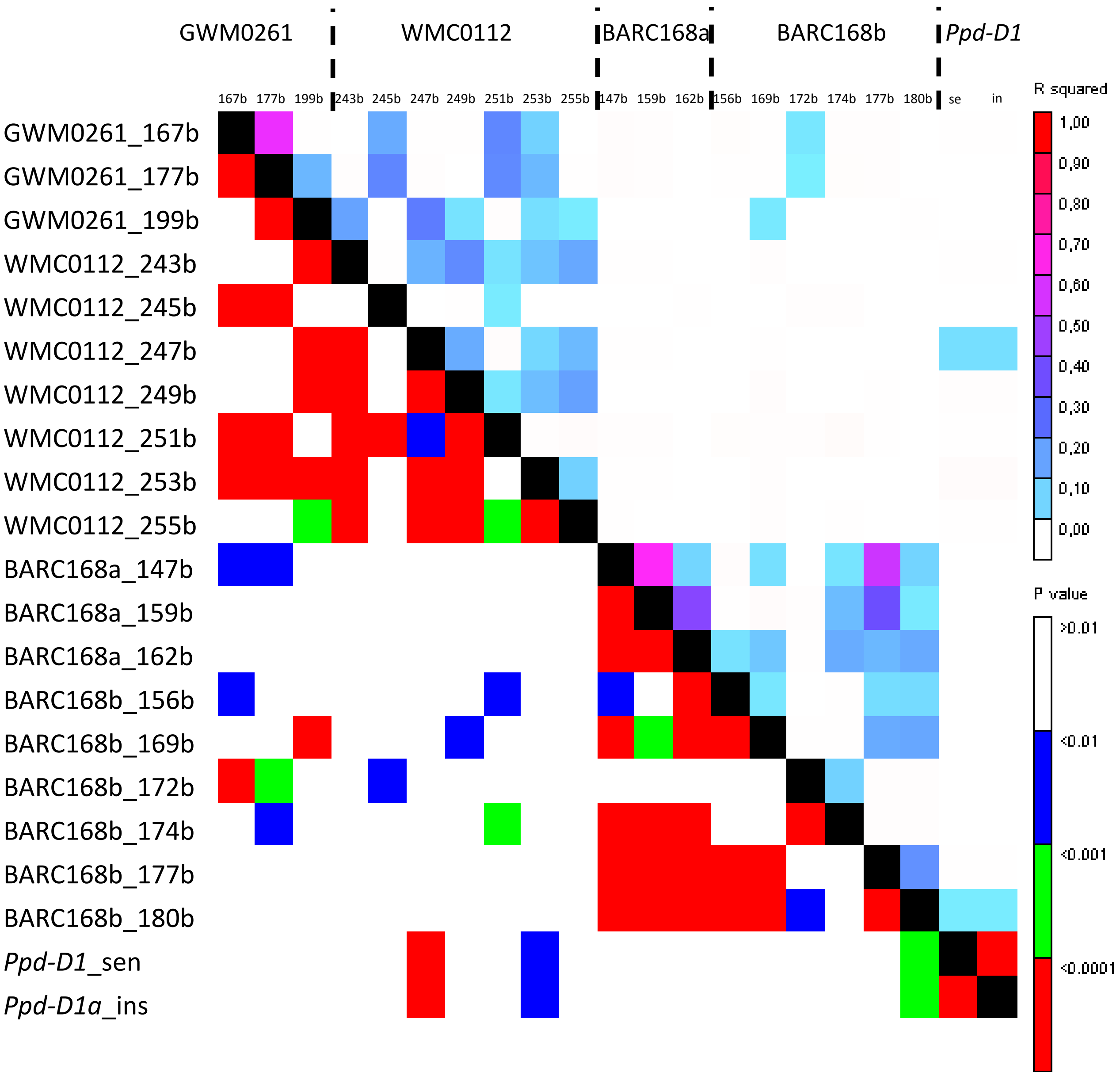

Supplemental file S15: The Linkage disequilibrium plot showed no LD with r^2 >0.1 between the alleles of markers GWM261,WMC112 or BARC168 and the *Ppd-D1* candidate gene.
